# Organocatalytic cascade aza-Michael/hemiacetal reaction between disubstituted hydrazines and α,β-unsaturated aldehydes: Highly diastereo- and enantioselective synthesis of pyrazolidine derivatives

**DOI:** 10.3762/bjoc.8.195

**Published:** 2012-10-09

**Authors:** Zhi-Cong Geng, Jian Chen, Ning Li, Xiao-Fei Huang, Yong Zhang, Ya-Wen Zhang, Xing-Wang Wang

**Affiliations:** 1 Key Laboratory of Organic Synthesis of Jiangsu Province, College of Chemistry, Chemical Engineering and Materials Science, Soochow University, Suzhou 215123, P. R. China; Fax: +86 (0)512 65880378; Tel: +86 (0)512 65880378

**Keywords:** aza-Michael, domino, hydrazine, organocatalysis, pyrazolidine

## Abstract

The catalytic synthesis of nitrogen-containing heterocycles is of great importance to medicinal and synthetic chemists, and also a challenge for modern chemical methodology. In this paper, we report the synthesis of pyrazolidine derivatives through a domino aza-Michael/hemiacetal sequence with chiral or achiral secondary amines as organocatalysts. Thus, a series of achiral pyrazolidine derivatives were obtained with good yields (up to 90%) and high diastereoselectivities (>20:1) with pyrrolidine as an organocatalyst, and enantioenriched pyrazolidines are also achieved with good results (up to 86% yield, >10/1 regioselectivity, >20:1 dr, 99% ee) in the presence of (*S*)-diphenylprolinol trimethylsilyl ether catalyst.

## Introduction

**Figure 1:**
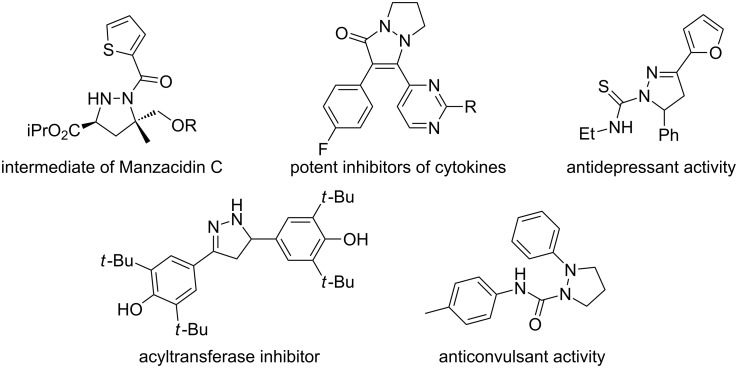
Important heterocycles containing pyrazolidine or pyrazoline structures.

Pyrazolidines are privileged and valuable heterocyclic compounds, which are of great importance in biological and medicinal chemistry (Figure 1) [1–5]. Besides, pyrazolidines are also important synthetic intermediates in organic chemistry. For instance, the N–N bond of pyrazolidines can be cleaved under reductive conditions to afford useful 1,3-diamines [6–7], and moreover, pyrazolidines can also be oxidized to afford pyrazolines [8–12] and pyrazoles [13–15]. The pyrazolidine structural unit is commonly constructed by [3 + 2] cycloaddition reactions using hydrazones [16–23] or azomethine amines [24–27] as dipoles. Recently, the Ma group and Toste et al. have reported efficient methods for the synthesis of pyrazolidine derivatives by metal-catalyzed aminations of allenes [28–32]. Meanwhile, Lewis acid catalyzed carboamination reactions have also been reported as efficient methods for the synthesis of pyrazolidine derivatives by Wolfe et al. [33].

In the past decade, the research area of organocatalysis has grown rapidly and become a third brand of catalysis besides the well-established biocatalysis and metal catalysis [34–44]. Particularly, organocatalytic domino/cascade reactions have come into focus and become a powerful synthetic approach that allows the construction of structurally diverse and complex molecules, minimizes the number of manual operations, and saves time, effort, and production costs [45–47]. Thus, many nitrogen-containing heterocyclic compounds have been efficiently generated by means of organocatalytic domino reactions [48–69].

In 2009 and 2010, List et al. and the Brière group both reported, separately, the enantioselective synthesis of 2-pyrazolines starting from α,β-unsaturated ketones and phenylhydrazine or *N*-*tert*-butyloxycarbonylhydrazine in the presence of a chiral Brønsted acid or a phase-transfer catalyst [70–71]. Compared with monosubstituted hydrazines in organocatalytic asymmetric synthesis, disubstituted hydrazines were also explored by several groups [72–73]. In 2007, Jørgensen et al. reported that the organocatalyzed asymmetric aza-Michael addition of hydrazones to cyclic enones had been achieved in good yield and stereoselectivity [74]. In 2011, the Deng group developed a highly enantioselective organocatalytic synthesis of 2-pyrazolines using disubstituted hydrazines through an asymmetric conjugate addition followed by a deprotection–cyclization sequence [75]. Due to the importance of pyrazolidine derivatives in both organic and medicinal chemistry, we have become interested in developing an efficient stereoselective cascade reaction for the synthesis of the pyrazolidine compounds through organocatalysis. In this paper, we present a convenient access to racemic and enantioenriched 5-hydroxypyrazolidines through a domino aza-Michael/hemiacetal organocatalytic sequence of disubstituted hydrazines to α,β-unsaturated aldehydes. While proceeding with the submission of our results, we noticed that a related excellent work has been reported by Vicario and co-workers [76]. When comparing Vicario’s work with this manuscript, both works are complementary in scope since Vicario’s work makes use of unsaturated aldehydes containing only linear alkyl chains, whereas our work provides better results when unsaturated aldehydes bearing an aromatic moiety are employed.

## Results and Discussion

First, the cascade aza-Michael/hemiacetal reactions between disubstituted hydrazines **2a**–**c** and 4-nitrocinnamaldehyde (**3a**) were investigated in the presence of several common secondary amines **1a**–**f** as organocatalysts in chloroform. Pyrrolidine (**1c**) turned out to be an effective catalyst and di-*tert*-butyl hydrazine-1,2-dicarboxylate (**2c**) was a potent donor (see Table S1 in the Supporting Information File 1). When di-*tert*-butyl hydrazine-1,2-dicarboxylate (**2c**) was used as the donor and 20 mol % pyrrolidine (**1c**) as the catalyst, 5-hydroxypyrazolidine **4a** could be obtained in 68% yield with over 20:1 dr after five days (Table 1, entry 1). Thus, the tandem aza-Michael/hemiacetal reaction between di-*tert*-butyl hydrazine-1,2-dicarboxylate (**2c**) and 4-nitrocinnamaldehyde (**3a**) was chosen as the model reaction to further optimize the reaction conditions. The catalytic results were summarized in Table 1. In order to improve the yield, a variety of common solvents were screened (Table 1, entries 2–6). Dichloromethane was finally found to be the best medium for this reaction (74% yield with excellent diastereoselectivity was achieved, Table 1, entry 2). Subsequently, the effects of some basic/and acidic additives were examined. Inorganic bases seemed to be ineffective for the further improvement of the yields (Table 1, entries 7–10). Soluble organic bases were also tested and failed to increase the yields (Table 1, entries 11 and 12). When benzoic acid (**5a**) was used as an additive, the yield was slightly improved to 77% (Table 1, entry 13). With the increase of the acidity, the yield was noticeably decreased (Table 1, entry 13 versus entries 14 and 15). Finally, when increasing the amount of di-*tert*-butyl hydrazine-1,2-dicarboxylate (**2c**) to 2 equiv, the desired product **4a** was obtained in 81% yield without any additive (Table 1, entry 16).

**Table 1:** Secondary amine catalyzed cascade aza-Michael/hemiacetal reaction.

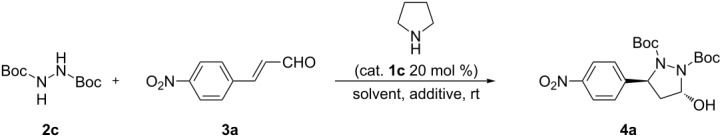				

Entry^a^	Solvent	Additive	dr^b^	Yield (%)^c^

1	CHCl_3_	–	>20:1	68
2	CH_2_Cl_2_	–	>20:1	74
3	MeOH	–	–	48
4	DMF	–	–	51
5	PhMe	–	–	42
6	THF	–	–	37
7	CH_2_Cl_2_	NaOAc	>20:1	68
8	CH_2_Cl_2_	NaHCO_3_	>20:1	63
9	CH_2_Cl_2_	Na_2_CO_3_	–	38
10	CH_2_Cl_2_	LiOAc	>20:1	56
11	CH_2_Cl_2_	Et_3_N	>20:1	42
12	CH_2_Cl_2_	DMAP	>20:1	50
13	CH_2_Cl_2_	benzoic acid (**5a**)	>20:1	77
14	CH_2_Cl_2_	4-nitrobenzoic acid (**5b**)	>20:1	67
15	CH_2_Cl_2_	3,5-dinitrobenzoic acid (**5c**)	–	56
16^d^	CH_2_Cl_2_	–	>20:1	81

^a^The reaction was run with **2c** (0.3 mmol), **3a** (0.25 mmol), **1c** (0.05 mmol) and the specified additive (0.05 mmol) in the given solvent (0.5 mL) at room temperature for 5 d. ^b^Determined by ^1^H NMR. ^c^Isolated yield. ^d^The molar ratio of **2c**/**3a** is 2.0:1.

**Figure 2:**
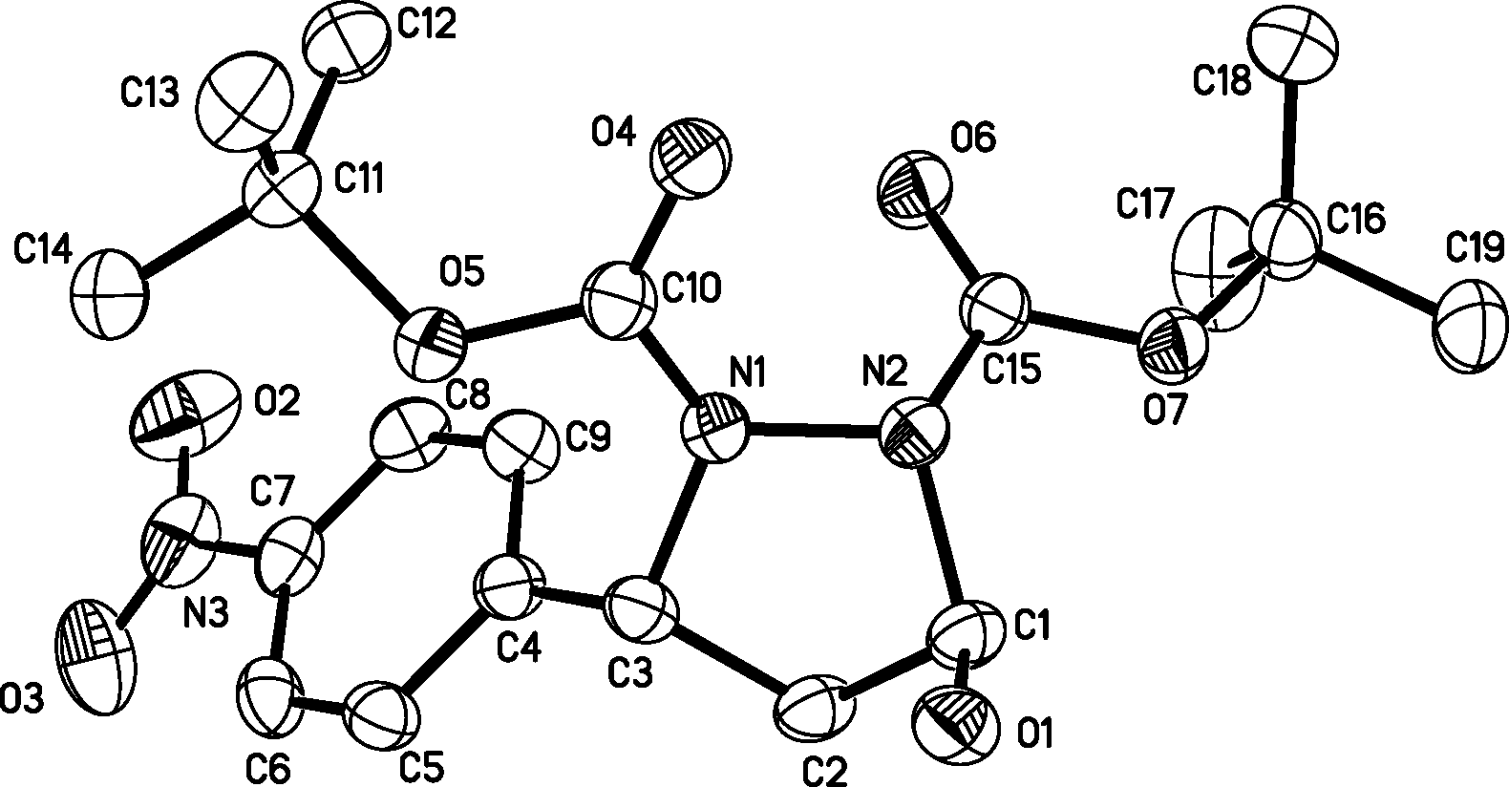
X-ray crystal structure of racemic **4a** (25% thermal ellipsoids).

**Figure 3:**
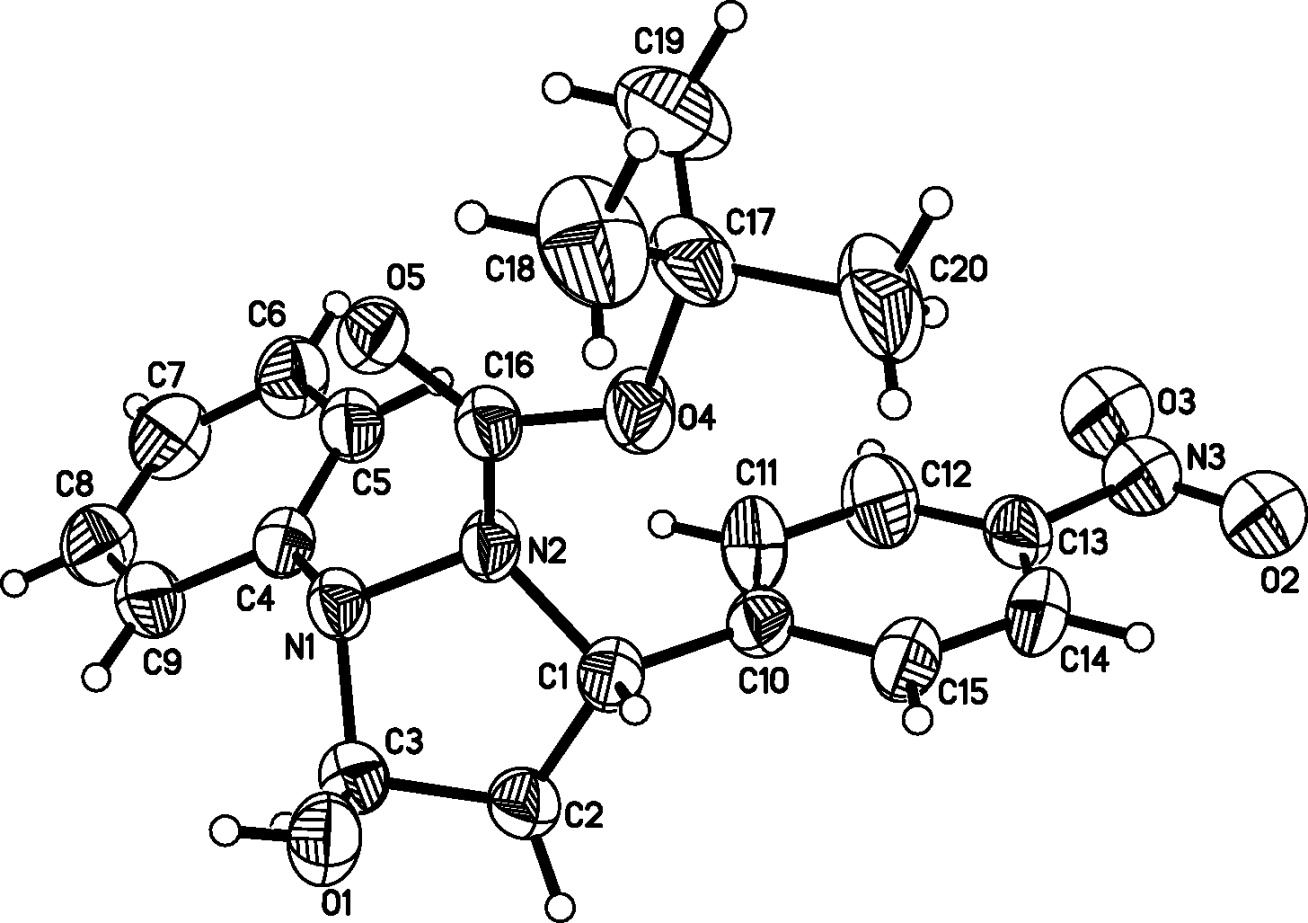
X-ray crystal structure of racemic **4n** (25% thermal ellipsoids).

Having established the optimized reaction conditions, we investigated the scope of substrates for the cascade aza-Michael/hemiacetal reaction with pyrrolidine (**1c**, 20 mol %) as the catalyst, without any additives, in methylene chloride (Table 2). We found that the nature of the substituents on the phenyl group of α,β-unsaturated aldehydes dramatically affected the reactivity. For example, with disubstituted hydrazine **2c** as the donor, the presence of a stronger electron-deﬁcient substituent (-NO_2_, -CN) on the phenyl ring of the α,β-unsaturated aldehydes **3a**, **3b** and **3c** promoted the cascade aza-Michael/hemiacetal reaction readily to provide the desired products in good yields (75–81%, Table 2, entries 1–3). The presence of less-electron-deﬁcient substituents (-Cl and -Br) on the phenyl ring of the α,β-unsaturated aldehydes, such as 4-chloro- or 4-bromocinnamaldehyde derivatives **3d** and **3e**, afforded the corresponding products in moderate yields (64 and 74%) even when 5 equiv of disubstituted hydrazine **2c** was used (Table 2, entries 4 and 5). When cinnamaldehyde derivatives **3f** and **3g**, bearing electron-donating substituents (-Me, -OMe) on phenyl rings, were used as the substrate, the reactions became very sluggish (Table 2, entries 6 and 7). On the other hand, more symmetric and asymmetric hydrazine derivatives **2d**–**h** were synthesized and investigated for the tandem aza-Michael/hemiacetal reaction. Generally, all the reactions between **2d**–**h** and α,β-unsaturated aldehydes **3a** and **3b** proceeded smoothly with sequential catalytic actions of **1c**, affording the corresponding desired products **4h**–**o** in moderate to good yields (Table 2, entries 8–11 and 13–16). Notably, the reaction between **2e** and 4-methoxycinnamaldehyde (**3f**) afforded the desired product in 52% yield (Table 2, entry 12). For the asymmetric disubstituted hydrazines **2g** and **2h** as substrates, regioselective results could be observed (Table 2, entry 14). However, with increasing catalyst loading from catalytic to stoichiometric amounts, the corresponding cascade reactions could provide the major products in 72 and 66% yields (Table 2, entries 15 and 16). In all the reactions, high diastereoselectivity could be obtained (>20:1 dr). Finally, we were able to obtain single crystals of compounds **4a** and **4n**, which allowed for an unambiguous assignment of the *trans* configuration of C3 and C5 by X-ray crystallographic analysis (Figure 2 and Figure 3).

**Table 2:** Substrate scope for the cascade aza-Michael/hemiacetal reactions between **2** and α,β-unsaturated aldehydes **3**.

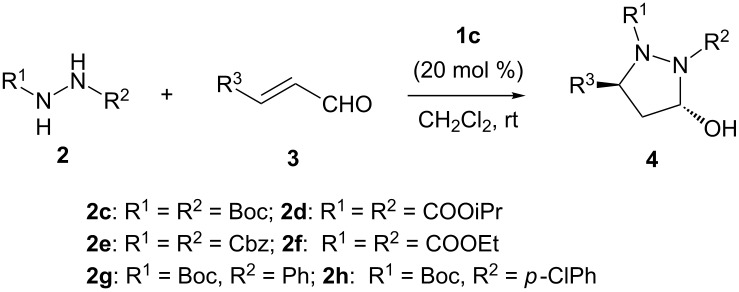				

Entry^a^	Donor	R^3^	dr^b^	Yield (%)^c^

1	**2c**	4-NO_2_C_6_H_4_ (**3a**)^d^	>20:1	**4a**/81
2	**2c**	3-NO_2_C_6_H_4_ (**3b**)^d^	>20:1	**4b**/78
3	**2c**	4-CNC_6_H_4_ (**3c**)^d^	>20:1	**4c**/75
4	**2c**	4-ClC_6_H_4_ (**3d**)^e^	>20:1	**4d**/64
5	**2c**	4-BrC_6_H_4_ (**3e**)^e^	>20:1	**4e**/74
6	**2c**	4-MeOC_6_H_4_ (**3f**)^d^	–	**4f/**<5
7	**2c**	4-MeC_6_H_4_ (**3g**)^d^	–	**4g/**<5
8	**2d**	4-NO_2_C_6_H_4_ (**3a**)^d^	>20:1	**4h**/86
9	**2d**	3-NO_2_C_6_H_4_ (**3b**)^d^	>20:1	**4i**/82
10	**2e**	4-NO_2_C_6_H_4_ (**3a**)^d^	>20:1	**4j**/90
11	**2e**	3-NO_2_C_6_H_4_ (**3b**)^d^	>20:1	**4k**/76
12	**2e**	4-MeOC_6_H_4_ (**3f**)^d^	>20:1	**4l**/52
13	**2f**	4-NO_2_C_6_H_4_ (**3a**)^d^	>20:1	**4m**/77
14	**2g**	4-NO_2_C_6_H_4_ (**3a**)^f^	>20:1	**4n**/20 (43)
15	**2g**	4-NO_2_C_6_H_4_ (**3a**)^f,g^	>20:1	**4n**/72 (<5)
16	**2h**	3-NO_2_C_6_H_4_ (**3b**)^f,g^	>20:1	**4o**/66 (<5)

^a^Reaction was conducted on 0.25 mmol scale in solvents (0.5 mL) at room temperature for five days. ^b^Determined by ^1^H NMR. ^c^Isolated yield (the data in parentheses is related to the isolated yield of the regioselective product). ^d^The ratio of **2**/**3** is 2.0:1. ^e^The ratio of **2**/**3** is 5.0:1. ^f^The ratio of **2**/**3** is 1.2:1. ^g^100 mol % of pyrrolidine was used at rt for 12 h.

Then, we turned our attention to the development of the asymmetric version of the cascade aza-Michael/hemiacetal reaction. Initially, a series of readily available chiral organocatalysts **1g**–**o** were chosen and investigated for the domino aza- Michael/hemiacetal reaction of disubstituted hydrazine **2c** and 4-nitrocinnamaldehyde (**3a**) under catalytic loading of 20 mol % in CH_2_Cl_2_ at room temperature. The screening results are summarized in Table 3. (*S*)-Proline derivatives **1g**–**h**, **1k**, **1l** were found to be ineffective for the reaction, because they afford only trace products after one day (Table 3, entries 1, 2, 5 and 6). Although a moderate yield was obtained with organocatalyst **1i** bearing a sulfone functional group, the stereochemical induction was very poor (Table 3, entry 3). MacMillan’s catalyst **1j** was proven to be inefﬁcient for this transformation as only 13% ee was obtained (Table 3, entry 4). Subsequently, three diarylprolinol silyl ether catalysts **1m**–**o** were investigated for this tandem reaction [77–79]. Gratifyingly, 82% ee was achieved when **1m** was used as the catalyst. For catalysts **1n** and **1o**, slightly higher yields could be obtained, but the enantioselectivities became lower (Table 3, entry 7 versus entries 8 and 9). Relatively speaking, (*S*)-diphenylprolinol trimethylsilyl ether **1m** turned out to be the optimal catalyst in terms of both enantioselectivity and reactivity.

**Table 3:** Chiral-amine-catalyzed cascade aza-Michael/hemiacetal reaction of **2c** with **3a**.

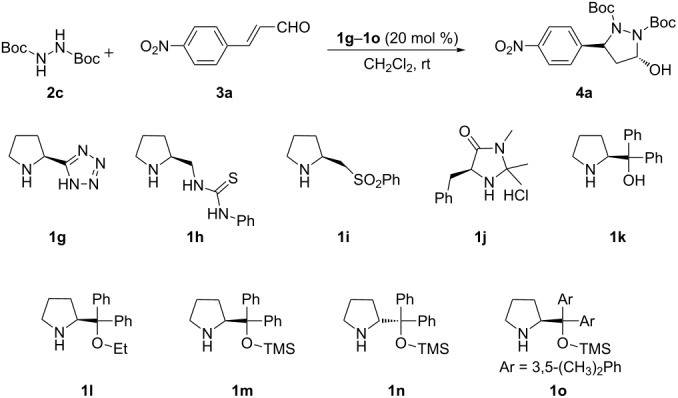				

Entry^a^	R	Time (d)	Yield (%)^b^	ee (%)^c^

1	**1g**	1	<10	n.d.
2	**1h**	1	<5	n.d.
3	**1i**	1	48	5
4	**1j**	1	21	13
5	**1k**	1	<10	n.d.
6	**1l**	1	<5	n.d.
7	**1m**	1	86	82
8	**1n**	1	89	(−)79^d^
9	**1o**	1	87	76

^a^The reaction was run with **2c** (0.5 mmol), **3a** (0.25 mmol), and **1** (0.05 mmol) in CH_2_Cl_2_ (0.5 mL) at room temperature. ^b^Isolated yield. ^c^Determined by HPLC analysis on a chiral stationary phase (Chiralcel OD-H), >20:1 dr. ^d^Opposite enantiomer of the product formed. n.d. = not determined.

Having identiﬁed the readily available catalyst **1m** as the optimal catalyst for the tandem aza-Michael/hemiacetal reaction of **2c** and **3a**, we summarize the results for the optimization of the other reaction parameters, including reaction solvents and additives, in Table 4. When the reaction was carried out in a protonic solvent, i.e., methanol, at room temperature for two days, the desired product was furnished in 56% yield with only 22% ee (Table 4, entry 1). After screening several aprotic solvents for this reaction, we were pleased to find that the enantioselectivity of the desired product was improved to 92% ee with toluene or THF as solvent after two days (Table 4, entries 4 and 5). Considering both yield and enantioselectivity, toluene was the optimal reaction medium (Table 4, entry 5). When the time was prolonged to four days, the yield of the product was increased to 80% and the enantioselectivity was retained (Table 4, entry 6). Thereafter, several Brønsted acids **5b**, **5d**–**j** were tested as additives for this transformation. Although enantioselectivity was somewhat improved from 92 to 95% ee, the reactivity dramatically decreased as evidenced by the prolonged reaction time and lower yields (Table 4, entry 6 versus entries 7–14). It seemed that the present catalytic system could be inhibited by acidic additives. Therefore, we considered whether the reaction could be accelerated by basic additives without loss of enantioselectivity and reactivity. Subsequently, several common inorganic and organic bases were investigated [80–83]. Unfortunately, the catalytic results showed that with LiOAc, DMAP, DABCO, Et_3_N, TMEDA as additives, the yield and enantioselectivity were only marginally influenced (Table 4, entries 15–19). When DBU was used as an additive, only 11% ee was obtained with moderate yield (Table 4, entry 20). Thus, **1m** (20 mol %) as the catalyst and toluene as the reaction medium without any additive at room temperature proved to be the optimal reaction conditions for the asymmetric cascade aza-Michael/hemiacetal reaction.

**Table 4:** Optimization of the reaction of **2c** and **3a** catalyzed by chiral amine **1m**.

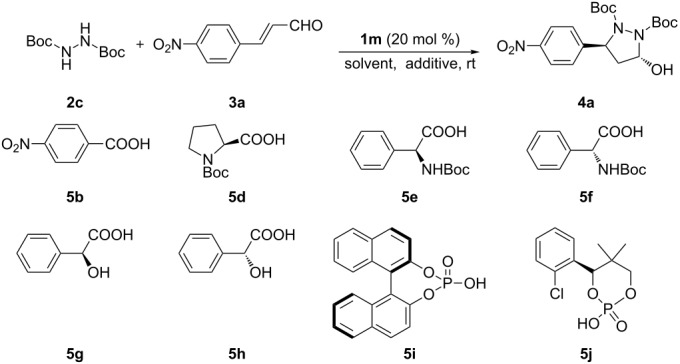					

Entry^a^	Additive	Solvent	Time (d)	Yield (%)^b^	ee (%)^c^

1	–	MeOH	2	56	22
2	–	CHCl_3_	1	84	75
3	–	CHCl_2_	1	86	82
4	–	THF	2	51	92
5	**–**	PhMe	2	72	92
6	–	PhMe	4	80	92
7	**5b**	PhMe	4	27	93
8	**5d**	PhMe	4	56	94
9	**5e**	PhMe	4	48	92
10	**5f**	PhMe	4	45	92
11	**5g**	PhMe	4	51	94
12	**5h**	PhMe	4	20	94
13	**5i**	PhMe	4	38	94
14	**5j**	PhMe	4	53	95
15	LiOAc	PhMe	4	79	93
16	DMAP	PhMe	4	83	87
17	DABCO	PhMe	4	79	89
18	Et_3_N	PhMe	4	83	87
19	TMEDA	PhMe	4	81	88
20	DBU	PhMe	4	58	11

^a^The reaction was run with **2c** (0.5 mmol), **3a** (0.25 mmol), **1m** (0.05 mmol) and the specified additive (0.05 mmol) in the given solvent (0.5 mL) at room temperature. ^b^Isolated yield. ^c^Determined by HPLC analysis on a chiral stationary phase (Chiralcel OD-H), >20:1 dr.

**Figure 4:**
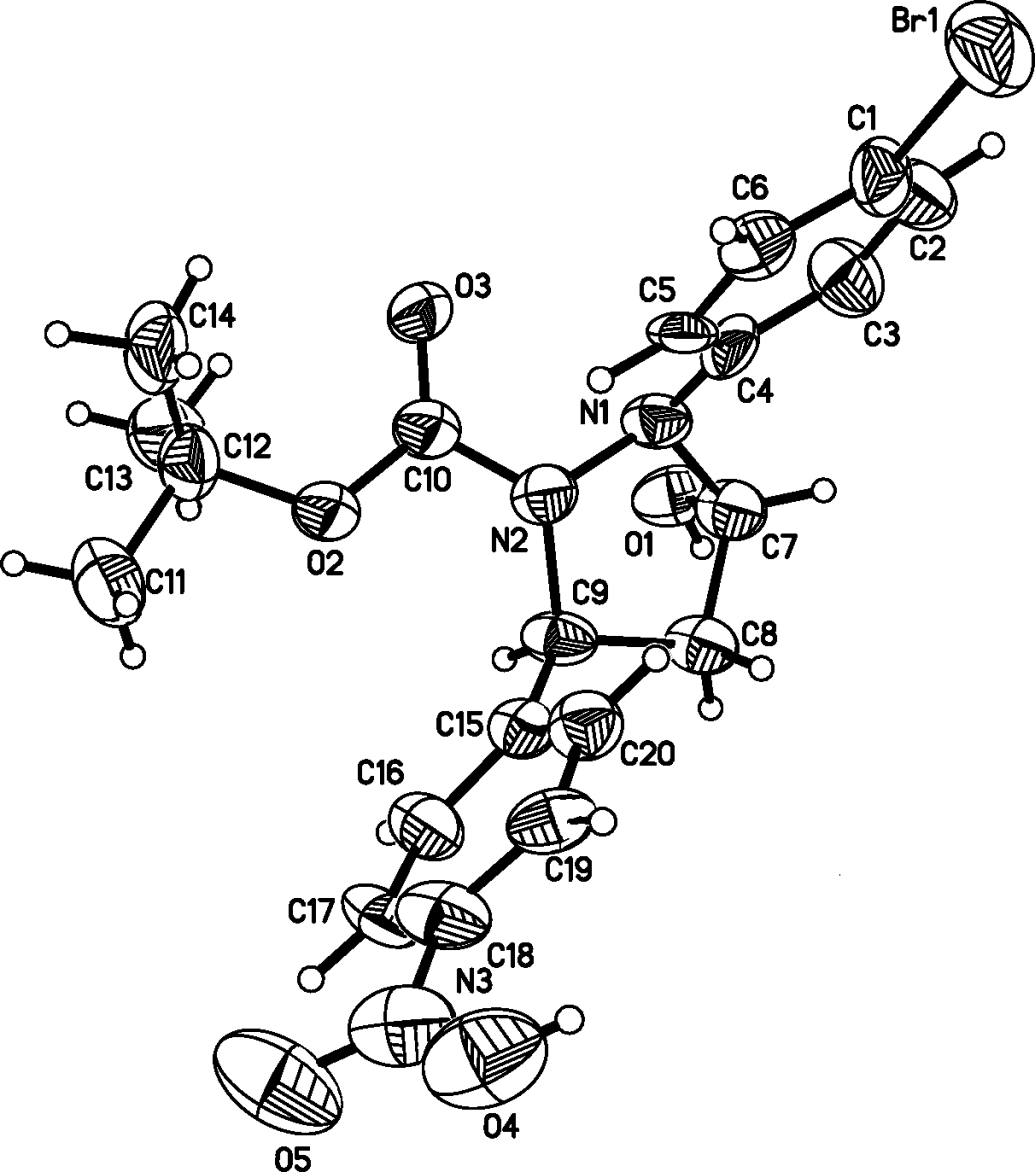
The X-ray crystal structure of chiral compound **4s** (40% thermal ellipsoids).

With the optimized reaction conditions in hand, the substrate scope of the organocatalyzed asymmetric domino aza-Michael/hemiacetal sequence was subsequently explored. Firstly, with symmetric di-*tert*-butyl hydrazine-1,2-dicarboxylate (**2c**) as nucleophilic reagent, aromatic α,β-unsaturated aldehydes **3a**–**g** were examined to study the effects of electronic properties and steric hindrance on both enantioselectivity and reactivity. For the substrates **3a**, **3b** and **3c**, bearing substituents of -NO_2_ and -CN at the *para*- or *meta*-position of the phenyl group, the reactions proceeded smoothly and led to the desired products **4a**, **4b** and **4c** in 80–86% yields with 89–92% ee’s (Table 5, entries 1–3). With **3d** and **3e** bearing -Cl or -Br substituents at the *para*-position of the phenyl group as substrates, the desired products **4d** and **4e** were obtained in 61 and 62% yields with 74 and 77% ee, respectively (Table 5, entries 4 and 5). For substrates **3f** and **3g** bearing electron-donating groups (-Me, -OMe) on the phenyl rings, only a trace amount of the desired products could be observed under otherwise identical reaction conditions (Table 5, entries 6 and 7). These experimental results indicated that chemical yields and enantioselectivities were dramatically affected by the electronic properties and steric hindrance of the aryl group on the α,β-unsaturated aldehydes. High yield and good enantioselectivity could be obtained with strong electron-withdrawing substituents on the phenyl ring of cinnamaldehydes. When diisopropyl hydrazine-1,2-dicarboxylate (**2d**) as nucleophilic reagent reacted with 4-nitro cinnamaldehyde (**3a**), the product **4h** was obtained in 60% yield and 72% ee (Table 5, entry 8). The result showed that the small-sized substituent on hydrazines was unfavorable on the reaction (Table 5, entry 8 versus entry 1). Subsequently, asymmetric disubstituted hydrazines **2g**–**j** were investigated for the domino aza-Michael/hemiacetal sequence. Due to nucleophilic competition of the two nitrogens in the asymmetric disubstituted hydrazines, regioselective results were observed for these reactions. For asymmetric disubstituted hydrazines **2g**, the reaction gave the 1.8:1 molar ratio of the regioselective products **4n** to **4n’**. The major product **4n** was obtained in 58% yield and 88% ee. The enantioselectivity of the minor product **4n’** was 55% (Table 5, entry 9). For asymmetric disubstituted hydrazines **2h**–**i**, the molar ratios of the regioselective products ranged from 3.2:1 to 6.4:1. The major products were obtained in moderate to good yields and good enantioselectivities (Table 5, entries 10–15). When disubstituted hydrazine **2j** with an electron-donating group on the aromatic ring was used as the nucleophilic donor, the major, reversely regioselective product **4u’** was obtained in 75% yield, but with very low enantioselectivity (11% ee, Table 5, entry 16). To our delight, (*E*)-but-2-enal (**3i**) was a suitable substrate for this transformation. The reactions of asymmetric disubstituted hydrazines **2h** and **2i** with (*E*)-but-2-enal (**3i**) proceeded smoothly and provided the desired products in 78 and 83% yields with 72 and 74% ee, respectively (Table 5, entries 17 and 18). However, when the cascade aza-Michael/hemiacetal reaction of (*E*)-pent-2-enal (**3j**) and **2h** was carried out, only a trace amount of the expected products could be detected (Table 5, entry 19). Fortunately, single crystals of compound **4s** were obtained by recrystallization from petroleum ether/acetyl acetate, and the absolute configuration was determined by X-ray analyses (Figure 4) [84].

**Table 5:** Substrate scope of **2** and **3** catalyzed by chiral amine **1m**.

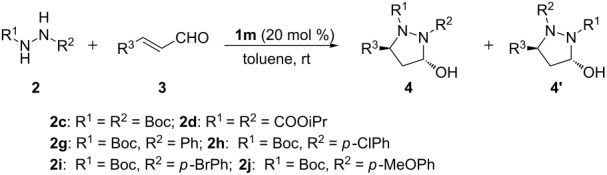						

Entry^a^	Donor	R^3^	Time (d)	Yield (%)^b^	Ratio^c^	ee (%)^d^

1	**2c**	4-NO_2_C_6_H_4_ (**3a**)	4	**4a**/80	–	92
2	**2c**	3-NO_2_C_6_H_4_ (**3b**)	2	**4b**/83	–	91
3	**2c**	4-CNC_6_H_4_ (**3c**)	4	**4c**/86	–	89
4	**2c**	4-ClC_6_H_4_ (**3d**)	4	**4d**/61	–	74
5	**2c**	4-BrC_6_H_4_ (**3e**)	4	**4e**/62	–	77
6	**2c**	4-MeOC_6_H_4_ (**3f**)	4	**4f**/<5	–	–
7	**2c**	4-MeC_6_H_4_ (**3g**)	4	**4g**/<5	–	–
8	**2d**	4-NO_2_C_6_H_4_ (**3a**)	2	**4h**/60	–	72
9	**2g**^e^	4-NO_2_C_6_H_4_ (**3a**)	4	**4n**/58 (**4n’**/32)	1.8:1	88/55
10	**2h**^e^	3-NO_2_C_6_H_4_ (**3b**)	4	**4o**/60	3.2:1	88
11	**2h**^e^	4-NO_2_C_6_H_4_ (**3a**)	4	**4p**/72	4.5:1	99
12	**2h**^e^	4-CNC_6_H_4_ (**3c**)	4	**4q**/65	4.7:1	93
13	**2h**^e^	3-CF_3_C_6_H_4_ (**3h**)	4	**4r**/53	–^g^	81
14	**2i**^e^	4-NO_2_C_6_H_4_ (**3a**)	4	**4s**/74	6.4:1	99
15	**2i**^e^	4-CNC_6_H_4_ (**3c**)	4	**4t**/72	4.0:1	90
16	**2j**^e^	4-NO_2_C_6_H_4_ (**3a**)	4	**4u'**/75	1:9.0	–/11
17	**2h**^f^	Me (**3i**)	2	**4v**/78	>10:1	72
18	**2i**^f^	Me (**3i**)	2	**4w**/83	>10:1	74
19	**2h**^f^	Et (**3j**)	3	**4x**/<10	–	–

^a^Unless noted, the reaction was run with **2** (0.5 mmol), **3** (0.25 mmol), and **1m** (0.05 mmol) in toluene (0.5 mL) at room temperature. ^b^Isolated yield of pure isomer **4** (the data in parentheses is related to the isolated yield of the **4’**). ^c^The ratio based on isolated yield of pure **4** and **4’**. ^d^Determined by HPLC analysis on a chiral stationary phase (Chiralcel OD-H, AD-H or AS-H), >20:1 dr. ^e^The ratio of **2**/**3** is 1.2:1. ^f^The reaction was run with **2** (0.25 mmol), **3** (0.38 mmol), and **1m** (0.05 mmol) in toluene (0.5 mL) at room temperature. ^g^Due to the difficulty of separation of the product **4r’** from starting material **2h**.

## Conclusion

In summary, we have developed an organocatalytic approach for the synthesis of pyrazolidine derivatives through the cascade aza-Michael/hemiacetal reaction between disubstituted hydrazines and α,β-unsaturated aldehydes. The asymmetric version of this one-pot cascade reaction has also been realized with (*S*)-diphenylprolinol trimethylsilyl ether **1m** as a secondary amine organocatalyst, and a series of enantiomerically enriched pyrazolidine derivatives were obtained in moderate to good chemical yields with moderate to excellent enantioselectivities. The application of the products and further investigation of the reaction are ongoing in our laboratory.

## Experimental

### Representative experimental procedure for the cascade aza-Michael/hemiacetal reaction of disubstituted hydrazines with α,β-unsaturated aldehydes

To a stirred solution of catalyst **1c** or **1m** (20 mol %) in CH_2_Cl_2_ or toluene (0.5 mL) was added α,β-unsaturated aldehyde **3** (1.0 equiv, 0.25 mmol) and di-substituted hydrazine **2** (1.2 equiv or 2.0 or 5.0 equiv and 0.3 mmol or 0.5 mmol or 1.25 mmol) at rt. The reaction was vigorously stirred for 2–5 days. Then, the reaction mixture was directly subjected to flash column chromatography on silica gel (petroleum ether/ethyl acetate) to afford the corresponding products **4**.

**(−)-Di-*tert*-butyl 3-hydroxy-5-(4-nitrophenyl)pyrazolidine-1,2-dicarboxylate (4a):** 80% yield, >20/1 dr, 92% ee. The enantiomeric ratio was determined by HPLC on Chiralpak OD-H column (10% 2-propanol/hexane, 1 mL/min), *t*_major_ = 7.219 min, *t*_minor_ = 6.013 min; [α]_D_^26^ −11.8 (*c* 0.38, acetone); ^1^H NMR (400 MHz, CDCl_3_) δ 8.20 (d, *J* = 8.6 Hz, 2H), 7.55 (d, *J* = 8.6 Hz, 2H), 5.92 (d, *J* = 4.7 Hz, 1H), 5.52–5.39 (m, 1H), 3.40 (s, 1H), 2.71 (dd, *J* = 13.2, 8.4 Hz, 1H), 2.13–2.03 (m, 1H), 1.53 (s, 9H), 1.43 (s, 9H); ^13^C NMR (100 MHz, CDCl_3_) δ 154.27, 149.72, 147.21, 126.75, 124.01, 82.62, 82.56, 82.19, 61.66, 43.43, 28.33, 28.19; IR (KBr) ν_max_: 3354.8, 2980.4, 2934.2, 2854.2, 1728.7, 1705.9, 1600.2, 1518.9, 1456.0, 1367.7, 1345.9, 1311.6, 1243.1, 1145.0, 990.9, 853.5, 758.4 cm^−1^; HRMS–ESI (*m*/*z*): [Na]^+^ calcd for C_19_H_27_N_3_O_7_, 432.1741; found, 432.1724.

## Supporting Information

File 1 General experimental procedures, ^1^H, ^13^C NMR spectra and HPLC chromatograms for all new compounds, crystal data and structure refinement for enantiopure **4**.
