# Supplementary material for: Organocatalytic cascade aza-Michael/hemiacetal reaction between disubstituted hydrazines and α,β-unsaturated aldehydes: Highly diastereo- and enantioselective synthesis of pyrazolidine derivatives
Source: Beilstein J Org Chem. 2012 Oct 9;8:1710–20. doi: 10.3762/bjoc.8.195 (PMC3511004; doi:10.3762/bjoc.8.195)
Supplement: File 1 — General experimental procedures, 1H, 13C NMR spectra and HPLC chromatograms for all new compounds, crystal data and structure refinement for enantiopure 4. [file Beilstein_J_Org_Chem-08-1710-s001.pdf]

# Supporting Information

# for

# Organocatalytic cascade aza-Michael/hemiacetal reaction between disubstituted hydrazines and α,β-unsaturated aldehydes: Highly diastereo- and enantioselective synthesis of pyrazolidine derivatives

Zhi-Cong Geng, Jian Chen, Ning Li, Xiao-Fei Huang, Yong Zhang, Ya-Wen Zhang* and Xing-Wang Wang*

Address: [1] Key Laboratory of Organic Synthesis of Jiangsu Province, College of Chemistry, Chemical Engineering and Materials Science, Soochow University, Suzhou 215123, P. R. China; Fax: +86 (0)512 65880378; Tel: +86 (0)512 65880378

Email: Xing-Wang Wang* - wangxw@suda.edu.cn

*Corresponding author

## General experimental procedures, $^{1}H$, $^{13}C$ NMR spectra and HPLC chromatograms for all new compounds, crystal data and structure refinement for enantiopure 4

1. General methods S2

2. Reaction optimization S2

3. Typical experimental procedure S3

4. X-ray data of racemic **4a**, **4n** and chiral **4s** S15

5. $^{1}H$ NMR and $^{13}C$ NMR spectra S21

6. HPLC spectra S43

## 1. General methods

All commercially available solvents and reagents were used as received. Purification of reaction products was carried out by flash chromatography using silica gel (0.03–0.04 mm). Analytical thin-layer chromatography was performed on silica-gel precoated glass plates (0.2 ± 0.03 mm thickness, GF-254, particle size 0.01–0.04 mm). $^{1}H$ and $^{13}C$ NMR spectra were recorded in $CDCl_3$ or DMSO-$d_6$ on a Varian Inova (400 MHz or 300 MHz and 100 MHz or 75 MHz, respectively) spectrometer. Chemical shifts (δ ppm) are relative to the resonance of the deuterated solvent as the internal standard ($CDCl_3$, δ 7.27 ppm, DMSO-$d_6$, δ 2.50 ppm for proton NMR; $CDCl_3$, δ 77.23 ppm, DMSO-$d_6$, δ 39.51 ppm for carbon NMR).

## 2. Reaction optimization

**Table S1:** Effect of catalysts and solvents.

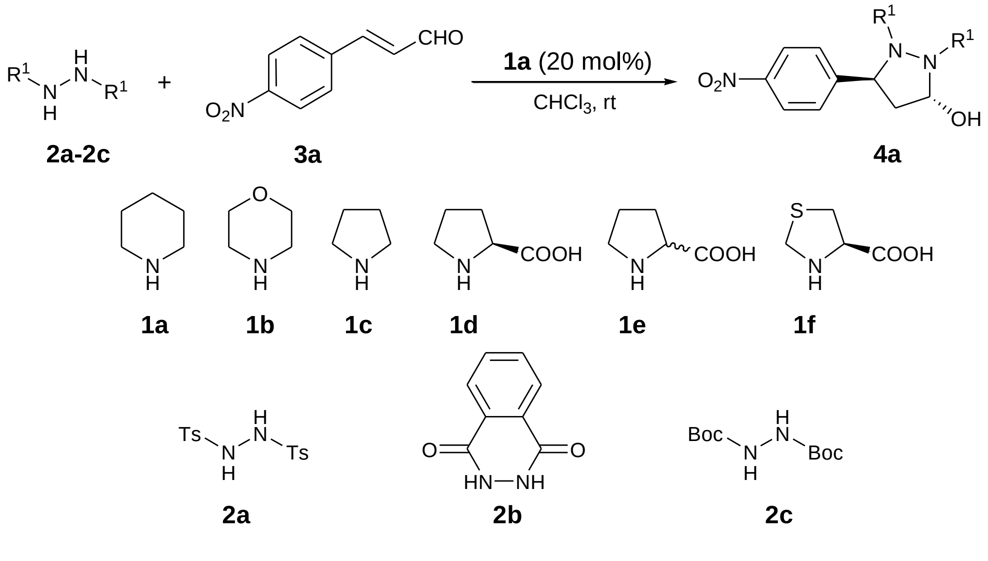

| Entry[a] | Catalyst | Donor | Additive | Solvent | dr[b] | Yield(%)[c] |
|---|---|---|---|---|---|---|
| 1 | **1a** | **2a** | – | $CHCl_3$ | – | – |
| **2** | **1a** | **2b** | **–** | **$CHCl_3$** | **–** | **–** |
| 3 | **1a** | **2c** | – | $CHCl_3$ | >20:1 | 40 |
| 4 | – | **2c** | – | $CHCl_3$ | – | – |
| 5 | **1b** | **2c** | – | $CHCl_3$ | – | <20 |
| 6 | **1c** | **2c** | – | $CHCl_3$ | >20:1 | 68 |
| 7 | **1d** | **2c** | – | $CHCl_3$ | – | <20 |
| 8 | **1e** | **2c** | – | $CHCl_3$ | – | <20 |
| 9 | **1f** | **2c** | – | $CHCl_3$ | – | <20 |

[a]Reaction was conducted on 0.25 mmol scale in solvents (0.5 mL) at room temperature for 5 d, and the ratio of **2**/**3** is 1.2:1. [b]Determined by $^1$H NMR. [c]Isolated yield.

## 3. Typical experimental procedure

Representative experimental procedure for the cascade aza-Michael/hemiacetal reaction of disubstituted hydrazines with α,β-unsaturated aldehydes: To a stirred solution of catalyst **1c** or **1m** (20 mol %) in $CH_2Cl_2$ or toluene (0.5 mL) was added α,β-unsaturated aldehyde **3** (1.0 equiv 0.25 mmol) and disubstituted hydrazine **2** (1.2 equiv or 2.0 or 5.0 equiv and 0.3 mmol or 0.5 mmol or 1.25 mmol) at rt. The reaction mixture was vigorously stirred for 2–5 days. Then, the reaction mixture was directly subjected to flash column chromatography on silica gel (petroleum ether/ethyl acetate) to afford the corresponding products **4**. Yields and spectral and analytical data for compounds **4a**–**x** are as follows:

**(−)-Di-*tert*-butyl 3-hydroxy-5-(4-nitrophenyl)pyrazolidine-1,2-dicarboxylate (4a):** 80% yield, >20/1 dr, 92% ee. The enantiomeric ratio was determined by HPLC on Chiralpak OD-H column (10% 2-propanol/hexane, 1 mL/min), $t_{major}$ = 7.219 min,

$t_{minor}$ = 6.013 min; $[\alpha]_D^{26}$ = −11.8 (*c* 0.38, acetone); $^1$H NMR (400 MHz, $CDCl_3$) δ 8.20 (d, *J* = 8.6 Hz, 2H), 7.55 (d, *J* = 8.6 Hz, 2H), 5.92 (d, *J* = 4.7 Hz, 1H), 5.52–5.39 (m, 1H), 3.40 (s, 1H), 2.71 (dd, *J* = 13.2, 8.4 Hz, 1H), 2.13–2.03 (m, 1H), 1.53 (s, 9H), 1.43 (s, 9H); $^{13}$C NMR (100 MHz, $CDCl_3$) δ 154.27, 149.72, 147.21, 126.75, 124.01, 82.62, 82.56, 82.19, 61.66, 43.43, 28.33, 28.19; IR (KBr) $\nu_{max}$: 3354.8, 2980.4, 2934.2, 2854.2, 1728.7, 1705.9, 1600.2, 1518.9, 1456.0, 1367.7, 1345.9, 1311.6, 1243.1, 1145.0, 990.9, 853.5, 758.4 cm$^{-1}$; HRMS (ESI): *m/z* = 432.1724 (calcd for $C_{19}H_{27}N_3O_7$+$Na^+$= 432.1741).

**(−)-Di-*tert*-butyl 3-hydroxy-5-(3-nitrophenyl)pyrazolidine-1,2-dicarboxylate (4b):** 83% yield, >20/1 dr, 91% ee. The enantiomeric ratio was determined by HPLC on Chiralpak OD-H column (10% 2-propanol/hexane, 1 mL/min), $t_{major}$ = 8.652 min, $t_{minor}$ = 5.858 min; $[\alpha]_D^{26}$ = −0.3 (*c* 0.61, acetone);$^1$H NMR (400 MHz, $CDCl_3$) δ 8.31 (s, 1H), 8.13 (d, *J* = 7.9 Hz, 1H), 7.66 (d, *J* = 7.7 Hz, 1H), 7.52 (t, *J* = 7.9 Hz, 1H), 5.94 (d, *J* = 5.1 Hz, 1H), 5.53–5.44 (m, 1H), 3.00 (s, 1H), 2.72 (dd, *J* = 13.3, 8.4 Hz, 1H), 2.19–2.09 (m, 1H), 1.56 (s, 9H), 1.45 (s, 9H).; $^{13}$C NMR (100 MHz, $CDCl_3$) δ 154.45, 148.70, 144.65, 132.31, 129.62, 122.36, 120.66, 82.79, 82.48, 82.09, 61.57, 43.62, 28.17; IR (KBr) $\nu_{max}$: 3432.4, 3077.2, 2979.6, 2934.8, 2873.1, 1711.0, 1681.4, 1535.4, 1479.5, 1455.1, 1392.7, 1367.5, 1252.3, 1170.0, 1303.9, 1278.7, 1144.2, 870.0, 807.8, 736.2 cm$^{-1}$; HRMS (ESI): *m/z* = 432.1742 (calcd for $C_{19}H_{27}N_3O_7$+$Na^+$= 432.1741).

**(−)-Di-*tert*-butyl 3-(4-cyanophenyl)-5-hydroxypyrazolidine-1,2-dicarboxylate (4c):** 86% yield, >20/1 dr, 89% ee. The enantiomeric ratio was determined by HPLC

on Chiralpak OD-H column (5% 2-propanol/hexane, 1 mL/min), $t_{major}$ = 12.326 min, $t_{minor}$ = 9.171 min; $[\alpha]_D^{26}$ = −8.3 (*c* 0.36, acetone); $^1$H NMR (400 MHz, $CDCl_3$) δ 7.63 (d, *J* = 8.0 Hz, 2H), 7.49 (d, *J* = 8.0 Hz, 2H), 5.90 (d, *J* = 4.4 Hz, 1H), 5.50–5.33 (m, 1H), 3.23 (s, 1H), 2.68 (dd, *J* = 13.0, 8.6 Hz, 1H), 2.20–1.95 (m, 1H), 1.51 (s, 9H), 1.43 (s, 9H); $^{13}$C NMR (100 MHz, $CDCl_3$) δ 154.37, 147.73, 132.56, 126.70, 118.87, 111.15, 82.66, 82.48, 82.08, 61.85, 43.41, 28.35, 28.22; IR (KBr) $\nu_{max}$: 3436.0, 2978.7, 2934.1, 2228.7, 1706.9, 1610.0, 1506.1, 1457.6, 1392.8, 1368.6, 1251.8, 1156.0, 1091.5, 850.2, 806.0, 760.4 $cm^{-1}$; HRMS (ESI): *m*/*z* = 422.1255 (calcd for $C_{20}H_{27}N_3O_5$+$Na^+$= 422.1242).

**(−)-Di-*tert*-butyl 3-(4-chlorophenyl)-5-hydroxypyrazolidine-1,2-dicarboxylate (4d):** 61% yield, >20/1 dr, 74% ee. The enantiomeric ratio was determined by HPLC on Chiralpak OD-H column (10% 2-propanol/hexane, 1 mL/min), $t_{major}$ = 5.676 min, $t_{minor}$ = 4.273 min; $[\alpha]_D^{26}$ = −7.1 (*c* 0.56, acetone); $^1$H NMR (400 MHz, $CDCl_3$) δ 7.32–7.28 (m, 4H), 5.89 (d, *J* = 5.2 Hz, 1H), 5.41–5.29 (m, 1H), 3.24 (s, 1H), 2.62 (dd, *J* = 13.2, 8.3 Hz, 1H), 2.19–2.07 (m, 1H), 1.50 (s, 9H), 1.43 (s, 9H); $^{13}$C NMR (100 MHz, $CDCl_3$) δ 154.47, 140.62, 132.80, 128.59, 127.28, 82.59, 82.05, 81.64, 61.46, 43.43, 28.23, 28.15; IR (KBr) $\nu_{max}$: 3406.6, 2980.1, 2935.6, 1720.5, 1591.3, 1494.0, 1454.8, 1367.3, 1332.9, 1245.4, 1184.6, 1088.6, 855.5, 823.7 $cm^{-1}$; HRMS (EI): *m*/*z* = 421.1500 (calcd for $C_{19}H_{27}ClN_2O_5$+$Na^+$ = 421.1501).

**(−)-Di-*tert*-butyl 3-(4-bromophenyl)-5-hydroxypyrazolidine-1,2-dicarboxylate (4e):** 62% yield, >20/1 dr, 77% ee. The enantiomeric ratio was determined by HPLC on Chiralpak OD-H column (10% 2-propanol/hexane, 1 mL/min), $t_{major}$ = 5.605 min,

$t_{minor}$ = 4.373min; $[\alpha]_D^{26}$ = −5.0 (*c* 0.54, acetone); $^1$H NMR (400 MHz, $CDCl_3$) δ 7.45 (d, *J* = 8.2 Hz, 2H), 7.24 (d, *J* = 8.1 Hz, 2H), 5.89 (d, *J* = 4.7 Hz, 1H), 5.39–5.27 (m, 1H), 3.33 (s, 1H), 2.62 (dd, *J* = 13.2, 8.3 Hz, 1H), 2.19–2.04 (m, 1H), 1.50 (s, 9H), 1.43 (s, 9H); $^{13}$C NMR (100 MHz, $CDCl_3$) δ 154.41, 141.13, 131.64, 127.69, 121.01, 82.68, 82.16, 81.75, 61.57, 43.36, 28.32, 28.24; IR (KBr) $\nu_{max}$: 3406.4, 2978.9, 2933.1, 1720.2, 1590.6, 1491.0, 1454.0, 1244.2, 1141.9, 1010.3, 855.3 cm$^{-1}$; HRMS (EI): *m*/*z* = 465.0994 (calcd for $C_{19}H_{27}BrN_2O_5+Na^+$ = 465.0996).

**(−)-Diisopropyl 3-hydroxy-5-(4-nitrophenyl)pyrazolidine-1,2-dicarboxylate (4h):** 60% yield, >20/1 dr, 72% ee. The enantiomeric ratio was determined by HPLC on Chiralpak OD-H column (10% 2-propanol/hexane, 1 mL/min), $t_{major}$ = 8.499 min, $t_{minor}$ = 9.345 min; $[\alpha]_D^{26}$ = −10.0 (*c* 0.36, acetone); $^1$H NMR (400 MHz, $CDCl_3$) δ 8.20 (d, *J* = 8.6 Hz, 2H), 7.55 (d, *J* = 8.6 Hz, 2H), 5.98 (d, *J* = 4.6 Hz, 1H), 5.56–5.48 (m, 1H), 5.10–4.93 (m, 2H), 3.33 (s, 1H), 2.75 (dd, *J* = 13.2, 8.5 Hz, 1H), 2.18–2.07 (m, 1H), 1.32 (d, *J* = 6.2 Hz, 6H), 1.27 (d, *J* = 6.2 Hz, 3H), 1.21 (d, *J* = 6.0 Hz, 3H); $^{13}$C NMR (100 MHz, $CDCl_3$) δ 154.93, 149.16, 147.22, 126.76, 123.92, 82.83, 70.88, 70.82, 61.80, 43.25, 22.06, 21.98, 21.96, 21.87; IR (KBr) $\nu_{max}$: 3364.8, 2990.1, 2930.4, 1745.6, 1699.4, 1597.9, 1518.8, 1345.9, 1170.1, 1000.2, 853.5 cm$^{-1}$; HRMS (EI): *m*/*z* = 404.1428 (calcd for $C_{19}H_{27}BrN_2O_5+Na^+$ = 404.1428).

**(±)-Diisopropyl 3-hydroxy-5-(3-nitrophenyl)pyrazolidine-1,2-dicarboxylate (4i):** 82% yield, >20/1 dr. $^1$H NMR (400 MHz, $CDCl_3$) δ 8.27 (s, 1H), 8.14 (d, *J* = 8.1 Hz, 1H), 7.69 (d, *J* = 7.7 Hz, 1H), 7.52 (t, *J* = 7.9 Hz, 1H), 5.99 (d, *J* = 4.4 Hz, 1H), 5.59–5.48 (m, 1H), 5.09–4.94 (m, 2H), 3.52 (s, 1H), 2.76 (dd, *J* = 13.2, 8.4 Hz, 1H),

2.22–2.11 (m, 1H), 1.37–1.31 (m, 6H), 1.28 (d, *J* = 6.2 Hz, 3H), 1.22 (d, *J* = 5.8 Hz, 3H); $^{13}$C NMR (100 MHz, $CDCl_3$) δ 157.56, 155.14, 148.68, 144.08, 132.32, 129.74, 122.54, 120.89, 82.91, 71.19, 70.96, 61.77, 43.37, 22.01; IR (KBr) $\nu_{max}$: 3511.5, 3265.9, 3106.8, 2929.3, 1587.9, 1479.3, 1383.5, 1332.8, 1222.8, 1146.8, 994.3, 918.2, 815.5, 744.9, 699.3, 635.6 cm$^{-1}$; HRMS (EI): *m*/*z* = 404.1427 (calcd for $C_{17}H_{23}N_3O_7$+$Na^+$ = 404.1428).

**(±)-Dibenzyl 3-hydroxy-5-(4-nitrophenyl)pyrazolidine-1,2-dicarboxylate (4j):** 90% yield, >20/1 dr. $^1$H NMR (400 MHz, $CDCl_3$) δ 8.04 (d, *J* = 8.8 Hz, 2H), 7.46–7.18 (m, 12H), 6.00 (d, *J* = 4.8 Hz, 1H), 5.52 (t, *J* = 7.8 Hz, 1H), 5.32 (d, *J* = 12.0 Hz, 1H), 5.18 (s, 2H), 5.10 (d, *J* = 12.0 Hz, 1H), 3.51(s, 1H), 2.72 (dd, *J* = 13.0, 9.0 Hz, 1H), 2.20-2.04 (m, 1H); $^{13}$C NMR (100 MHz, $CDCl_3$) 157.64, 155.22, 148.40, 147.24, 135.61, 135.34, 128.76, 128.56, 128.44, 128.29, 127.60, 126.81, 124.00, 83.16, 68.62, 68.50, 62.12, 43.15. IR (film) $\nu_{max}$: 3437.2, 3112.6, 3066.6, 3033.5, 2954.1, 1712.2, 1601.7, 1521.9, 1455.8, 1395.9, 1306.4, 1215.2, 1156.6, 1079.7, 851.7 cm$^{-1,}$ HRMS (EI): *m*/*z* = 478.1609 (calcd for $C_{25}H_{24}N_3O_7$+$Na^+$ = 478.1609).

**(±)-Dibenzyl 3-hydroxy-5-(3-nitrophenyl)pyrazolidine-1,2-dicarboxylate (4k):** 76% yield, >20/1 dr. $^1$H NMR (400 MHz, $CDCl_3$) δ 8.21 (s, 1H), 8.09 (d, *J* = 8.1 Hz, 1H), 7.61 (d, *J* = 7.5 Hz, 1H), 7.39 (t, *J* = 8.0 Hz, 1H), 7.36–7.19 (m, 10H), 6.01 (d, *J* = 4.2 Hz, 1H), 5.55 (t, *J* = 7.6 Hz, 1H), 5.29–5.11 (m, 4H), 3.20 (s, 1H), 2.74 (dd, *J* = 13.2, 8.3 Hz, 1H), 2.22–2.12 (m, 1H); $^{13}$C NMR (100 MHz, $CDCl_3$) δ 157.76, 155.31, 148.59, 143.36, 135.63, 135.45, 132.19, 129.88, 128.73, 128.62, 128.60, 128.37, 128.33, 127.69, 122.64, 121.17, 83.24, 68.72, 68.61, 62.09, 43.21.; IR (film)

$\nu_{max}$: 3436.9, 2989.7, 2949.3, 1693.4, 1584.5, 1529.2, 1455.7, 1343.8, 1303.8, 1097.0, 865.0, 696.0 cm$^{-1}$; HRMS (EI): *m*/*z* = 478.1611 (calcd for $C_{25}H_{24}N_3O_7$+Na$^+$ = 478.1609).

**(±)-Dibenzyl 3-hydroxy-5-(4-methoxyphenyl)pyrazolidine-1,2-dicarboxylate (4l):** 52% yield, >20/1 dr. $^1$H NMR (400 MHz, $CDCl_3$) δ 7.36–7.20 (m, 10H), 7.16 (d, *J* = 8.5 Hz, 2H), 6.75 (d, *J* = 8.5 Hz, 2H), 6.01–5.93 (m, 1H), 5.43(t, *J* = 7.2 Hz, 1H), 5.26 (d, *J* = 12.3 Hz, 1H), 5.17 (s, 2H), 5.09 (d, *J* = 12.2 Hz, 1H), 3.76 (s, 3H), 3.66 (s, 1H), 2.57 (dd, *J* = 13.1, 8.3 Hz, 1H), 2.28–2.17 (m, 1H); $^{13}$C NMR (100 MHz, $CDCl_3$) δ 158.96, 157.88, 155.44, 136.04, 135.75, 132.88, 128.64, 128.51, 128.37, 128.23, 128.07, 127.56, 127.28, 114.08, 83.33, 68.26, 68.15, 62.21, 55.38, 43.08; IR (film) $\nu_{max}$: 3440.6, 3033.0, 2955.6, 2836.8, 1711.9, 1612.2, 1514.2, 1455.6, 1396.2, 1304.3, 1247.6, 1029.2, 829.4 cm$^{-1}$; HRMS (EI): *m*/*z* = 485.1683 (calcd for $C_{26}H_{26}N_2O_6$+Na$^+$ = 485.1683).

**(±)-Diethyl 3-hydroxy-5-(4-nitrophenyl)pyrazolidine-1,2-dicarboxylate (4m):** 77% yield, >20/1 dr. $^1$H NMR (400 MHz, $CDCl_3$) δ 8.20 (d, *J* = 8.7 Hz, 2H), 7.55 (d, *J* = 8.6 Hz, 2H), 5.98 (d, *J* = 5.2 Hz, 1H), 5.53 (t, *J* = 7.9 Hz, 1H), 4.34–4.16 (m, 4H), 3.01 (s, 1H), 2.76 (ddd, *J* = 13.3, 8.4, 1.1 Hz, 1H), 2.20–2.10 (m, 1H), 1.33 (t, *J* = 7.1 Hz, 3H), 1.25 (t, *J* = 7.1 Hz, 3H); $^{13}$C NMR (100 MHz, $CDCl_3$) δ 157.94, 155.30, 148.93, 147.37, 126.84, 124.13, 82.97, 63.24, 63.12, 62.08, 43.39, 14.63, 14.49; IR (film) $\nu_{max}$: 3434.7, 2983.1, 2937.4, 1712.1, 1601.6, 1522.1, 1468.1, 1347.4, 1224.9, 1173.4, 1013.1, 853.4 cm$^{-1,}$ HRMS (EI): *m*/*z* = 376.1116 (calcd for $C_{15}H_{19}N_3O_7$+Na$^+$ = 376.1115).

**(+)-*tert*-Butyl 3-hydroxy-5-(4-nitrophenyl)-2-phenylpyrazolidine-1-carboxylate (4n):** 58% yield, >20/1 dr, 88% ee. The enantiomeric ratio was determined by HPLC on Chiralpak OD-H column (10% 2-propanol/hexane, 1 mL/min), $t_{major}$ = 6.570 min, $t_{minor}$ = 5.722 min; $[\alpha]_D^{26}$ = +40.6 (*c* 0.48, acetone); $^1$H NMR (400 MHz, $CDCl_3$) δ 8.18 (d, *J* = 8.4 Hz, 2H), 7.49 (d, *J* = 8.4 Hz, 2H), 7.31 (t, *J* = 7.8 Hz, 2H), 6.98–7.07 (m, 3H), 5.69 (d, *J* = 3.6 Hz, 1H), 5.35 (dd, *J* = 10.0, 7.6 Hz, 1H), 3.51 (s, 1H), 2.68 (dd, *J* = 13.0, 7.4 Hz, 1H), 1.97–2.12 (m, 1H), 1.40 (s, 9H); $^{13}$C NMR (75 MHz, $CDCl_3$) δ 156.24, 150.23, 147.77, 146.54, 128.84, 128.07, 123.49, 120.63, 114.99, 87.81, 80.44, 61.38, 41.98, 27.78; IR (KBr) $\nu_{max}$: 3402.3, 2976.6, 1703.1, 1598.6, 1524.9, 1489.7, 1428.4, 1347.0, 1228.2, 1142.2, 989.4, 848.1, 752.1, 695.5 cm$^{-1}$; HRMS (EI): *m*/*z* = 408.1543 (calcd for $C_{20}H_{23}N_3O_5$+$Na^+$ = 408.1530).

**(−)-*tert*-Butyl 5-hydroxy-3-(4-nitrophenyl)-2-phenylpyrazolidine-1-carboxylate (4n’):** 32% yield, >20/1 dr, 55% ee. The enantiomeric ratio was determined by HPLC on Chiralpak OD-H column (10% 2-propanol/hexane, 1 mL/min), $t_{major}$ = 7.745 min, $t_{minor}$ = 8.266 min; $[\alpha]_D^{26}$ = −3.7 (*c* 0.41, acetone); $^1$H NMR (400 MHz, $CDCl_3$) δ 8.25 (d, *J* = 8.8 Hz, 2H), 7.71 (d, *J* = 8.4 Hz, 2H), 7.25–7.20 (m, 2H), 6.95 (t, *J* = 7.6 Hz, 1H), 6.89 (d, *J* = 8.0 Hz, 2H), 5.92 (m, 1H), 5.07 (t, *J* = 7.0 Hz, 1H), 2.86 (s, 1H), 2.74 (m, 1H), 2.28 (m, 1H), 1.49 (s, 9H); $^{13}$C NMR (100 MHz, DMSO-$d_6$) δ 160.29, 151.24, 151.00, 146.75, 128.46, 127.08, 123.96, 119.75, 113.50, 82.23, 80.71, 66.49, 44.13, 27.91; IR (KBr) $\nu_{max}$: 3416.4, 3070.8, 2979.1, 2941.0, 1686.2, 1595.9, 1520.7, 1490.9, 1454.3, 1435.7, 1370.0, 1261.3, 1147.5, 1079.9, 1011.7, 826.2, 763.6, 715.7 cm$^{-1}$; HRMS (EI): *m*/*z* = 386.1718 (calcd for $C_{20}H_{23}N_3O_5$+$H^+$ = 386.1710).

**(+)-*tert*-Butyl 2-(4-chlorophenyl)-3-hydroxy-5-(3-nitrophenyl)pyrazolidine-1-carboxylate (4o):** 60% yield, >20/1 dr, 88% ee. The enantiomeric ratio was determined by HPLC on Chiralpak AD-H column (5% 2-propanol/hexane, 1 mL/min), $t_{major}$ = 11.474 min, $t_{minor}$ = 18.868 min; $[\alpha]_D^{26}$ = +4.1 (*c* 0.27, acetone); $^1$H NMR (400 MHz, $CDCl_3$) δ 8.24 (s, 1H), 8.15 (d, *J* = 8.0 Hz, 2H), 7.63 (d, *J* = 7.6 Hz, 1H), 7.51 (t, *J* = 8.0 Hz, 2H), 7.24 (d, *J* = 8.8 Hz, 2H), 6.98 (d, *J* = 8.8, 1H), 5.65 (d, *J* = 7.6, 1H), 5.36 (dd, *J* = 9.8, 7.8 Hz, 1H), 4.19 (s, 1H), 2.68 (dd, *J* = 12.8, 7.6 Hz, 1H), 2.16–1.99 (m, 1H), 1.45 (s, 9H); $^{13}$C NMR (75 MHz, $CDCl_3$) δ 157.98, 149.29, 148.50, 145.90, 143.89, 133.36, 129.70, 129.42, 127.53, 122.77, 117.26, 89.13, 82.84, 62.41, 41.83, 28.43; IR (KBr) $\nu_{max}$: 3415.4, 2980.4, 1698.2, 1646.7, 1527.7, 1489.2, 1448.3, 1348.1, 1259.7, 1147.6, 1014.9, 904.1, 826.7, 781.6 cm$^{-1}$; HRMS (EI): *m*/*z* = 442.1148 (calcd for $C_{20}H_{22}ClN_3O_5$+$Na^+$ = 442.1140).

**(+)-*tert*-Butyl 2-(4-chlorophenyl)-3-hydroxy-5-(4-nitrophenyl)pyrazolidine-1-carboxylate (4p):** 72% yield, >20/1 dr, 99% ee. The enantiomeric ratio was determined by HPLC on Chiralpak AD-H column (5% 2-propanol/hexane, 0.8 mL/min), $t_{major}$ = 13.126 min, $t_{minor}$ = 15.819 min; $[\alpha]_D^{26}$ = +21.1 (*c* 0.18, acetone); $^1$H NMR (400 MHz, DMSO-$d_6$) δ 8.19 (d, *J* = 7.6 Hz, 2H), 7.60 (d, *J* = 7.6 Hz, 2H), 7.31 (d, *J* = 7.6 Hz, 2H), 7.01 (d, *J* = 8.0 Hz, 2H), 6.53 (s, 1H), 5.58 (s, 1H), 5.30 (t, *J* = 8.0 Hz, 1H), 3.30–3.23 (m, 1H), 2.08–1.84 (m, 1H), 1.30 (s, 9H); $^{13}$C NMR (75 MHz, DMSO-$d_6$) δ 156.05, 149.99, 146.65, 128.64, 128.13, 124.17, 123.57, 116.50, 113.16, 87.75, 80.71, 61.42, 41.99, 27.77; IR (KBr) $\nu_{max}$: 3405.9, 2983.5, 2938.5, 1698.3, 1598.2, 1523.9, 1487.8, 1428.1, 1350.3, 1367.5, 1235.6, 1147.8,

1090.9, 999.4, 832.8 $cm^{-1}$; HRMS (EI): $m/z$ = 442.1150 (calcd for $C_{20}H_{22}ClN_3O_5+Na^+$ = 442.1140).

**(+)-*tert*-Butyl 2-(4-chlorophenyl)-5-(4-cyanophenyl)-3-hydroxypyrazolidine-1-carboxylate (4q):** 65% yield, >20/1 dr, 93% ee. The enantiomeric ratio was determined by HPLC on Chiralpak AS-H column (5% 2-propanol/hexane, 1 mL/min), $t_{major}$ = 13.462 min, $t_{minor}$ = 16.982 min; $[\alpha]_D^{26}$ = +23.3 ($c$ 0.18, acetone); $^1H$ NMR (400 MHz, $CDCl_3$) δ 7.62 (d, $J$ = 6.8 Hz, 2H), 7.42 (d, $J$ = 7.2 Hz, 2H), 7.26–7.17 (m, 2H), 6.81 (d, $J$ = 7.6 Hz, 2H), 5.63 (s, 1H), 5.29 (t, $J$ = 8.8, 1H), 3.72 (s, 1H), 2.64 (dd, $J$ = 12.6, 7.4 Hz, 1H), 2.15-1.89 (m, 1H) 1.41 (s, 9H); $^{13}C$ NMR (100 MHz, DMSO-$d_6$) δ 156.25, 147.85, 146.65, 132.33, 128.59, 127.80, 124.12, 118.75, 116.44, 109.92, 89.13, 82.84, 62.41, 41.83, 28.43; IR (KBr) $\nu_{max}$: 3372.8, 2980.5, 2229.4, 1693.5, 1609.9, 1487.9, 1445.5, 1376.8., 1350.9, 1146.0, 1090.2, 824.2 $cm^{-1}$; HRMS (EI): $m/z$ = 422.1255 (calcd for $C_{21}H_{22}ClN_3O_3+Na^+$ = 422.1242).

**(−)-*tert*-Butyl 2-(4-chlorophenyl)-3-hydroxy-5-(3-(trifluoromethyl)phenyl) pyrazolidine-1-carboxylate (4r):** 53% yield, >20/1 dr, 81% ee. The enantiomeric ratio was determined by HPLC on Chiralpak AD-H column (10% 2-propanol/hexane, 1 mL/min), $t_{major}$ = 4.422 min, $t_{minor}$ = 5.016 min; $[\alpha]_D^{26}$ = −42.9 ($c$ 0.07, acetone); $^1H$ NMR (400 MHz, $CDCl_3$) δ 7.60 (s, 1H), 7.54 (d, $J$ = 6.8 Hz, 1H), 7.50–7.39 (m, 2H), 7.23 (d, $J$ = 8.4 Hz, 2H), 6.98 (d, $J$ = 8.4 Hz, 2H), 5.72–5.56 (m, 1H), 5.39–5.25 (m, 1H), 4.05 (s, 1H), 2.65 (dd, $J$ = 12.8, 7.6 Hz, 1H), 2.15–1.99 (m, 1H), 1.42 (s, 9H); $^{13}C$ NMR (100 MHz, DMSO-$d_6$) δ 155.96, 146.76, 143.61, 130.79, 129.38, 129.01, 128.53, 125.51, 124.30, 123.89, 122.81, 116.52, 87.86, 80.49, 61.59, 42.20,

27.69; IR (KBr) $\nu_{max}$: 3421.5, 2999.8, 1697.8, 1639.5, 1486.4, 1446.5, 1327.5, 1212.0, 1158.3, 1117.6, 991.4, 902.5, 823.3, 798.3, 780.4, 700.0 $cm^{-1}$; HRMS (EI): *m*/*z* = 465.1172 (calcd for $C_{21}H_{22}ClF_3N_2O_3+Na^+$ = 465.1183).

**(+)-*tert*-Butyl 2-(4-bromophenyl)-3-hydroxy-5-(4-nitrophenyl)pyrazolidine-1-carboxylate (4s):** 74% yield, >20/1 dr, 99% ee. The enantiomeric ratio was determined by HPLC on Chiralpak AD-H column (5% 2-propanol/hexane, 1 mL/min), $t_{major}$ = 12.291 min, $t_{minor}$ = 14.194 min; $[\alpha]_D^{26}$ = +29.2 (*c* 0.24, acetone); $^1$H NMR (400 MHz, DMSO-$d_6$) δ 8.19 (d, *J* = 8.0 Hz, 2H), 7.60 (d, *J* = 8.4 Hz, 2H), 7.43 (d, *J* = 8.0 Hz, 2H), 6.96 (d, *J* = 8.0 Hz, 2H), 6.54 (s, 1H), 5.58 (s, 1H), 5.30 (t, *J* = 8.6 Hz, 1H), 3.30–3.23 (m, 1H), 2.06–1.79 (m, 1H), 1.30 (s, 9H); $^{13}$C NMR (75 MHz, DMSO-$d_6$) δ 149.97, 147.04, 146.60, 131.50, 128.13, 123.58, 116.96, 111.92, 87.70, 80.73, 61.39, 42.01, 27.77; IR (KBr) $\nu_{max}$: 3408.1, 2982.4, 2936.0, 1697.3, 1598.3, 1523.6, 1485.2, 1426.7, 1349.3, 1236.9, 1146.7, 1092.0, 1000.8, 847.5 $cm^{-1}$; HRMS (EI): *m*/*z* = 486.0643 (calcd for $C_{20}H_{22}BrN_3O_5+Na^+$ = 486.0635).

**(+)-*tert*-Butyl 2-(4-bromophenyl)-5-(4-cyanophenyl)-3-hydroxypyrazolidine-1-carboxylate (4t):** 72% yield, >20/1 dr, 90% ee. The enantiomeric ratio was determined by HPLC on Chiralpak AD-H column (5% 2-propanol/hexane, 1 mL/min), $t_{major}$ = 13.878 min, $t_{minor}$ = 19.994 min; $[\alpha]_D^{26}$ = +12.2 (*c* 0.41, acetone); $^1$H NMR (400 MHz, $CDCl_3$) δ 7.62 (d, *J* = 7.6 Hz, 2H), 7.42 (d, *J* = 8.4 Hz, 2H), 7.38 (d, *J* = 8.4 Hz, 2H), 6.91 (d, *J* = 8.4 Hz, 2H), 5.68–5.57 (m, 1H), 5.40–5.19 (m, 1H), 3.69 (s, 1H), 2.64 (dd, *J* = 13.0, 7.4 Hz, 1H), 2.10–1.93 (m, 1H) 1.41 (s, 9H);

$^{13}C$ NMR (75 MHz, DMSO-$d_6$) δ 156.16, 147.87, 147.03, 132.35, 131.46, 127.81, 118.77, 116.90, 111.84, 109.90, 87.58, 80.63, 61.62, 42.00, 27.76; IR (KBr) $\nu_{max}$: 3416.6, 2982.0, 2228.1, 1693.8, 1609.0, 1586.1, 1485.2, 1420.0, 1381.1, 1353.4, 1145.3, 1094.8, 823.5 $cm^{-1}$; HRMS (EI): *m*/*z* = 466.0745 (calcd for $C_{21}H_{22}BrN_3O_3+Na^+$ = 466.0737).

**(−)-*tert*-Butyl 5-hydroxy-2-(4-methoxyphenyl)-3-(4-nitrophenyl)pyrazolidine-1-carboxylate (4u'):** 75% yield, >20/1 dr, 11% ee. The enantiomeric ratio was determined by HPLC on Chiralpak OD-H column (10% 2-propanol/hexane, 1 mL/min), $t_{major}$ = 9.927 min, $t_{minor}$ = 10.830 min; $[\alpha]_D^{26}$ = −6.8 (*c* 0.37, acetone); $^1H$ NMR (400 MHz, $CDCl_3$) δ 8.23 (d, *J* = 10.0 Hz, 2H), 7.70 (d, *J* = 9.6 Hz, 2H), 6.94 (d, *J* = 9.6 Hz, 2H), 6.81 (d, *J* = 9.2 Hz, 2H), 5.83 (s, 1H), 4.91 (s, 1H), 3.77 (s, 1H), 3.01 (s, 1H), 2.70 (m, 1H), 2.35 (m, 1H), 1.46 (s, 9H); $^{13}C$ NMR (100 MHz, $CDCl_3$) δ 155.17, 155.06, 149.87, 147.45, 145.08, 127.18, 124.15, 117.58, 114.28, 82.73, 82.04, 69.22, 55.65, 42.49, 28.43; IR (KBr) $\nu_{max}$: 3419.3, 3109.9, 2975.7, 2944.2, 1677.4, 1598.1, 1508.4, 1474.4, 1390.5, 1345.4, 1239.1, 1174.2, 1091.2, 998.8, 842.5 $cm^{-1}$; HRMS (EI): *m*/*z* = 416.1832 (calcd for $C_{21}H_{25}N_3O_6+H^+$ = 416.1816).

**(+)-*tert*-Butyl 2-(4-chlorophenyl)-3-hydroxy-5-methylpyrazolidine-1-carboxylate (4v):** 78% yield, >20/1 dr, 72% ee. The enantiomeric ratio was determined by HPLC on Chiralpak AD-H column (8% 2-propanol/hexane, 1 mL/min), $t_{major}$ = 7.352 min, $t_{minor}$ = 5.753 min; $[\alpha]_D^{26}$ = +27.8 (*c* 1.07, acetone); $^1H$ NMR (400 MHz, $CDCl_3$) δ 7.20 (d, *J* = 8.8 Hz, 2H), 6.98 (d, *J* = 8.8 Hz, 2H), 5.92 (dd, *J* = 8.0, 5.6 Hz, 1H),

4.02–3.82 (m, 1H), 3.15 (s, 1H), 2.25–2.12 (m, 1H), 2.12–1.94 (m, 1H), 1.44 (s, 9H), 1.28 (d, $J$ = 6.4 Hz, 1H); $^{13}$C NMR (100 MHz, $CDCl_3$) δ 155.26, 150.22, 128.78, 126.78, 118.20, 83.09, 81.74, 62.44, 40.18, 28.44, 20.65. IR (KBr) $\nu_{max}$: 3453.5, 2975.7, 1679.4, 1480, 1402.2, 1087.3, 952.2, 841.5 cm$^{-1}$; HRMS (EI): $m/z$ = 335.1134 (calcd for $C_{15}H_{21}ClN_2O_3$+$Na^+$ = 335.1133).

**(+)-*tert*-Butyl 2-(4-bromophenyl)-3-hydroxy-5-methylpyrazolidine-1-carboxylate (4w):** 83% yield, >20/1 dr, 74% ee. The enantiomeric ratio was determined by HPLC on Chiralpak AD-H column (8% 2-propanol/hexane, 1 mL/min), $t_{major}$ = 8.184 min, $t_{minor}$ = 6.063 min; $[\alpha]_D^{26}$ = +32.9 ($c$ 1.19, acetone); $^1$H NMR (400 MHz, $CDCl_3$) δ 7.34 (d, $J$ = 8.8 Hz, 2H), 6.92 (d, $J$ = 8.8 Hz, 2H), 6.00–5.84 (m, 1H), 4.10–3.81 (m, 1H), 3.13 (s, 1H), 2.30–2.10 (m, 1H), 2.10–1.95 (m, 1H), 1.44 (s, 9H), 1.28 (d, $J$ = 6.8 Hz, 1H) ; $^{13}$C NMR (100 MHz, $CDCl_3$) δ 155.35, 150.68, 131.61, 118.35, 113.99, 82.97, 81.69, 62.19, 40.35, 28.38, 20.69. IR (KBr) $\nu_{max}$: 3336.8, 2974.5, 1692.1, 1482.1, 1356.5, 1145.3, 1066.6, 828.8 cm$^{-1}$; HRMS (EI): $m/z$ = 357.0810 (calcd for $C_{15}H_{21}BrN_2O_3$+$H^+$ = 357.0808).

## 4. X-ray crystal structure of 4a, 4n, 4s

**4a**

| | | |
|---|---|---|
| Identification code | shelxl | |
| Empirical formula | $C_{19}H_{27}N_3O_7$ | |
| Formula weight | 409.44 | |
| Temperature | 223(2) K | |
| Wavelength | 0.71075 A | |
| Crystal system, space group | Monoclinic, C 2/c | |
| Unit cell dimensions | a = 27.633(9) A | alpha = 90 deg. |
| | b = 11.435(3) A | beta = 124.665(7) deg. |
| | c = 16.853(6) A | gamma = 90 deg. |
| Volume | 4380(2) A^3 | |
| Z, Calculated density | 8,1.242 Mg/m^3 | |
| Absorption coefficient | 0.095 mm^−1 | |
| F(000) | 1744 | |
| Crystal size | 0.76 x 0.75 x 0.42 mm | |
| Theta range for data collection | 3.08 to 25.50 deg. | |
| Limiting indices | −27<=h<=33, −13<=k<=13, −20<=l<=17 | |
| Reflections collected / unique | 10678 / 4039 [R(int) = 0.0391] | |
| Completeness to theta = 25.50 | 99.0% | |
| Absorption correction | Semi-empirical from equivalents | |
| Max. and min. transmission | 0.962 and 0.485 | |
| Refinement method | Full-matrix least-squares on F^2 | |
| Data / restraints / parameters | 4039 / 15 / 270 | |
| Goodness-of-fit on F^2 | 1.020 | |
| Final R indices [I>2sigma(I)] | R1 = 0.0606, wR2 = 0.1585 | |
| R indices (all data) | R1 = 0.0997, wR2 = 0.1867 | |
| Largest diff. peak and hole | 0.208 and −0.267 e.A^-3 | |

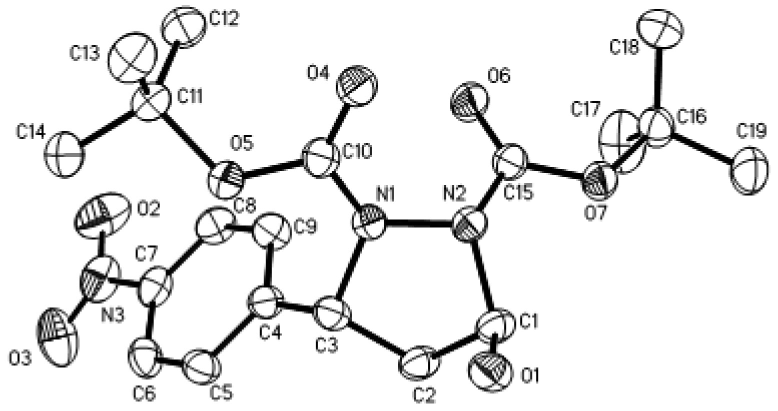


**Figure S1.** ORTEP drawing of racemic **4a** (25% thermal ellipsoids).

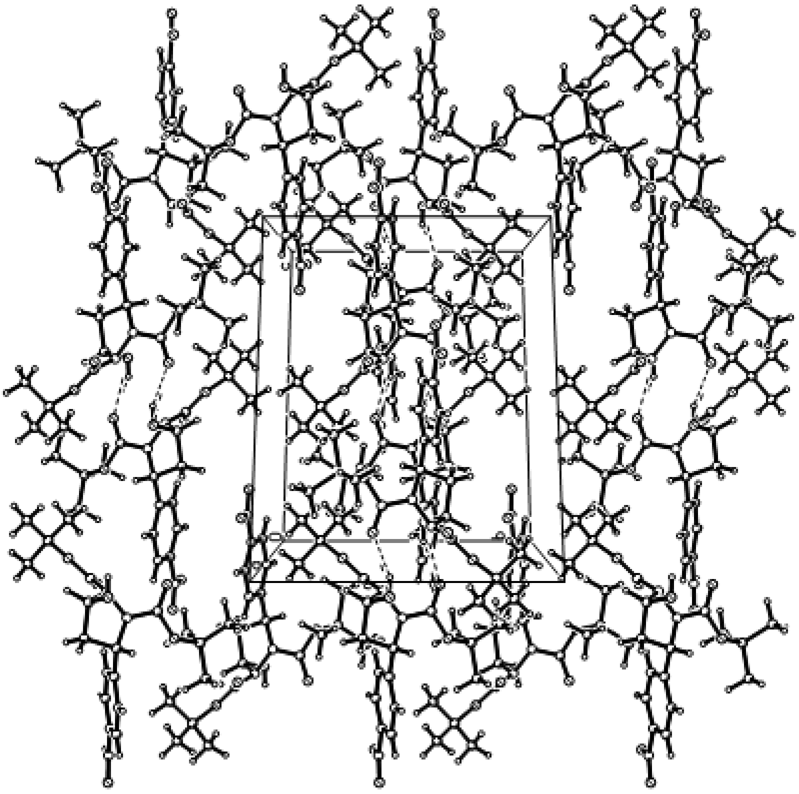

**Figure S2.** Packing of molecules in a unit cell for racemic **4a**.

**4n**

| | | |
|---|---|---|
| Identification code | shelxl | |
| Empirical formula | $C_{20}H_{23}N_3O_5$ | |
| Formula weight | 385.41 | |
| Temperature | 293(2) K | |
| Wavelength | 0.71070 A | |
| Crystal system, space group | Triclinic, P −1 | |
| Unit cell dimensions | a = 7.7399(14) A | alpha = 98.408(4) deg. |
| | b = 9.2877(17) A | beta = 103.948(4) deg. |
| | c = 14.911(3) A | gamma = 97.043(4) deg. |
| Volume | 1015.1(3) A^3 | |
| Z, Calculated density | 2, 1.261 Mg/m^3 | |
| Absorption coefficient | 0.092 mm^−1 | |
| F(000) | 408 | |
| Crystal size | 0.80 x 0.60 x 0.20 mm | |
| Theta range for data collection | 3.25 to 25.35 deg. | |
| Limiting indices | −9<=h<=7, −11<=k<=11, −17<=l<=17 | |
| Reflections collected / unique | 9794 / 3688 [R(int) = 0.0221] | |
| Completeness to theta = 25.35 | 99.1 % | |
| Absorption correction | Semi-empirical from equivalents | |
| Max. and min. transmission | 0.995 and 0.809 | |
| Refinement method | Full-matrix least-squares on F^2 | |
| Data / restraints / parameters | 3688 / 0 / 255 | |
| Goodness-of-fit on F^2 | 1.080 | |
| Final R indices [I>2sigma(I)] | R1 = 0.0758, wR2 = 0.1919 | |
| R indices (all data) | R1 = 0.0899, wR2 = 0.2037 | |
| Largest diff. peak and hole | 0.492 and −0.574 e.A^−3 | |

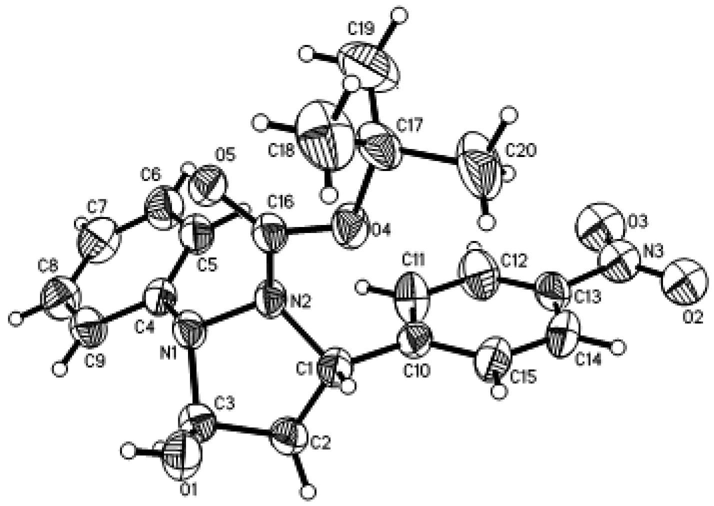


**Figure S3.** ORTEP drawing of racemic **4n** (25% thermal ellipsoids).

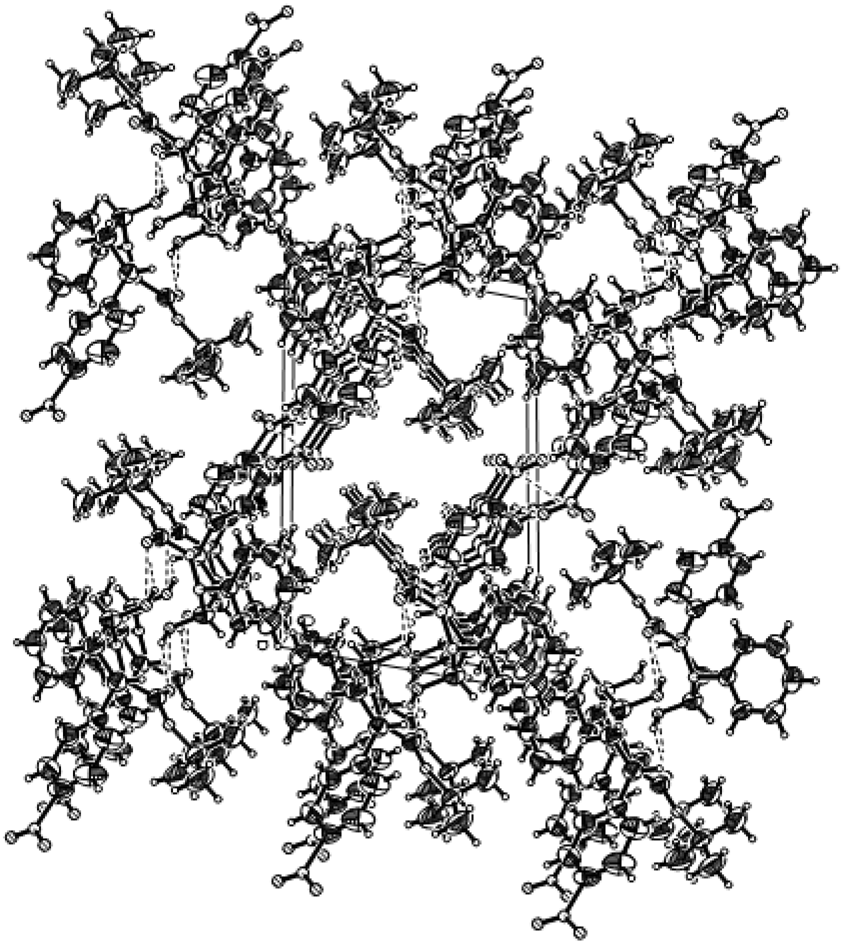

**Figure S4.** Packing of molecules in a unit cell for racemic **4n**.

**4s**

| | | |
|---|---|---|
| Identification code | shelxl | |
| Empirical formula | $C_{20}H_{22}BrN_3O_5$ | |
| Formula weight | 464.32 | |
| Temperature | 293(2) K | |
| Wavelength | 0.71070 A | |
| Crystal system, space group | Monoclinic, P 21 | |
| Unit cell dimensions | a = 8.140(3) A | alpha = 90 deg. |
| | b = 27.227(10) A | beta = 90 deg. |
| | c = 9.566(5) A | gamma = 90 deg. |
| Volume | 2120.1(16) A^3 | |
| Z, Calculated density | 4, 1.455 Mg/m^3 | |
| Absorption coefficient | 1.974 mm^−1 | |
| F(000) | 952 | |
| Crystal size | 0.50 x 0.27 x 0.20 mm | |
| Theta range for data collection | 3.09 to 25.49 deg. | |
| Limiting indices | −9<=h<=9, -32<=k<=32, −11<=l<=11 | |
| Reflections collected / unique | 15685 / 3740 [R(int) = 0.1609] | |
| Completeness to theta = 25.49 | 97.6 % | |
| Absorption correction | Semi-empirical from equivalents | |
| Max. and min. transmission | 0.673 and 0.331 | |
| Refinement method | Full-matrix least-squares on F^2 | |
| Data / restraints / parameters | 3740 / 77 / 261 | |
| Goodness-of-fit on F^2 | 1.115 | |
| Final R indices [I>2sigma(I)] | R1 = 0.1185, wR2 = 0.2012 | |
| R indices (all data) | R1 = 0.2120, wR2 = 0.2443 | |
| Absolute structure parameter | 0.02(3) | |
| Largest diff. peak and hole | 0.428 and −0.293 e.A^−3 | |

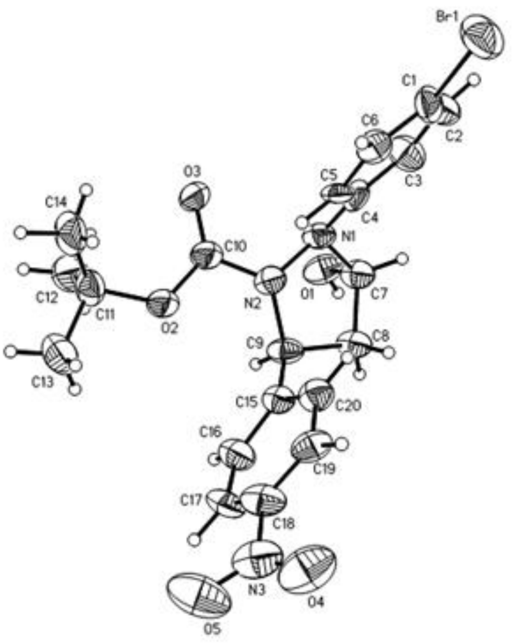


**Figure S5.** ORTEP drawing of chiral **4s** (40% thermal ellipsoids).

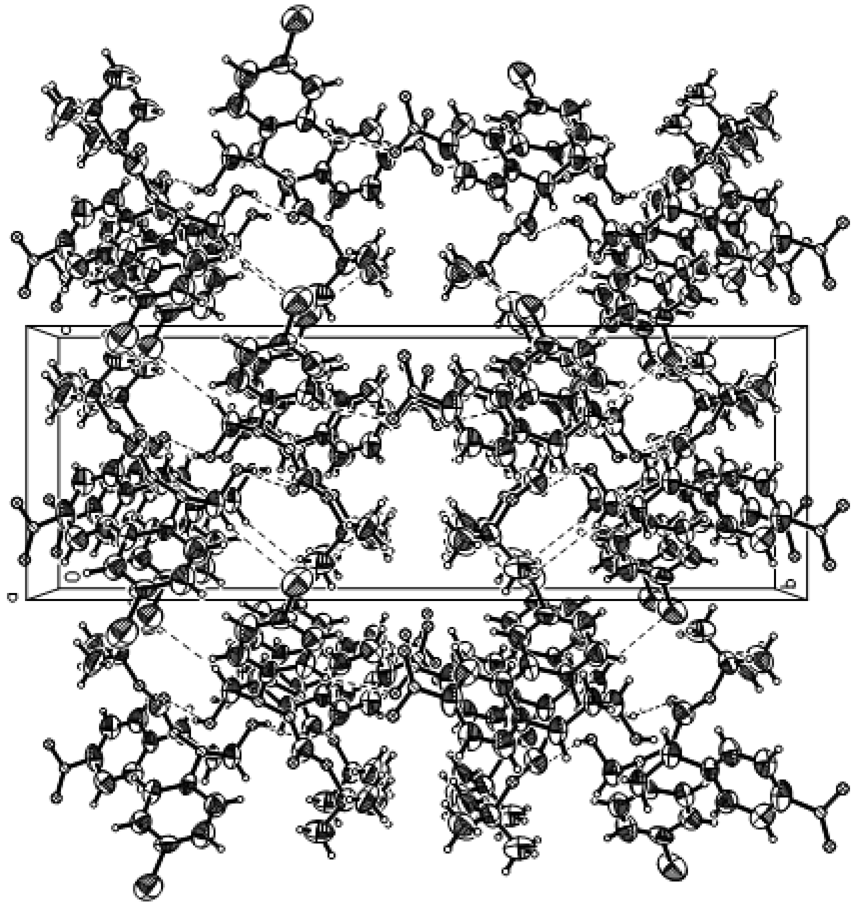

**Figure S6.** Packing of molecules in a unit cell for chiral **4s**.

## 5. $^1$H NMR and $^{13}$C NMR spectra

## 4a

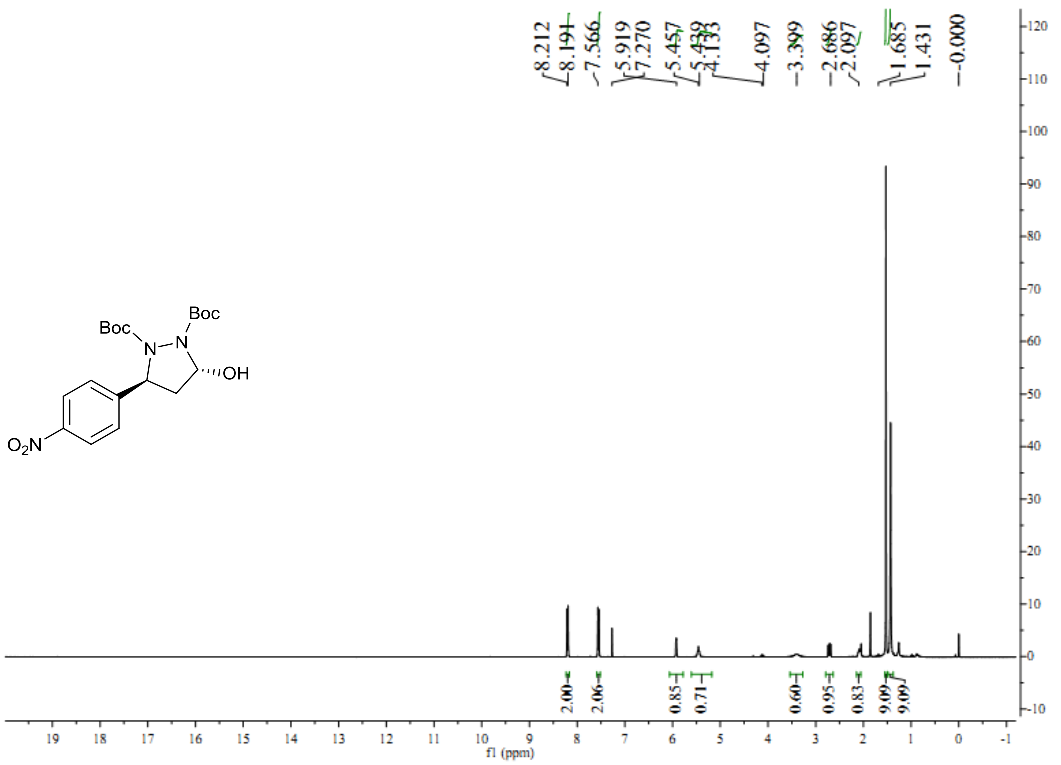

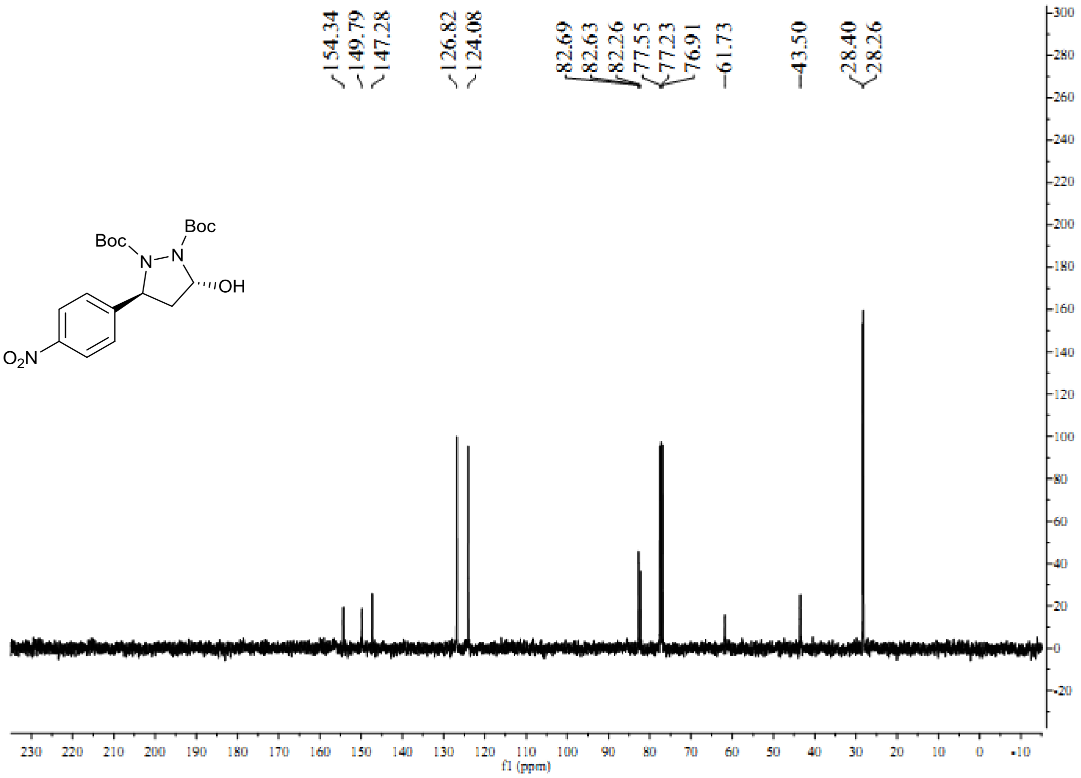

**4b**

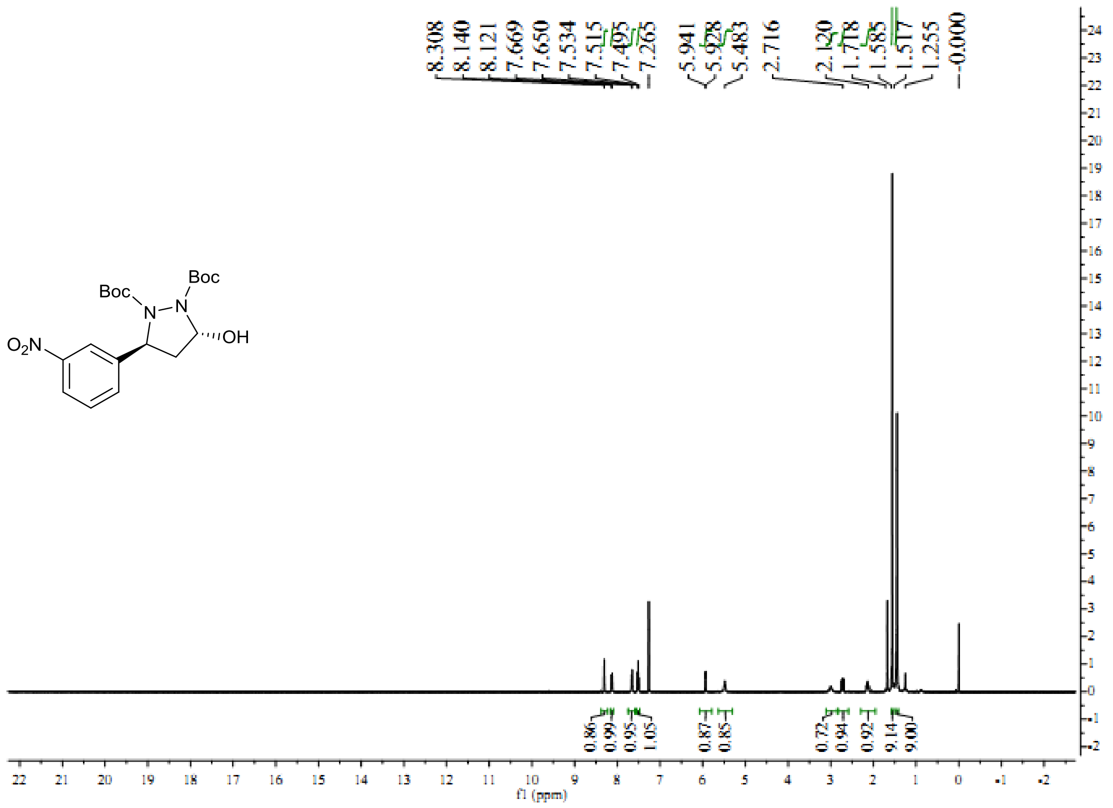

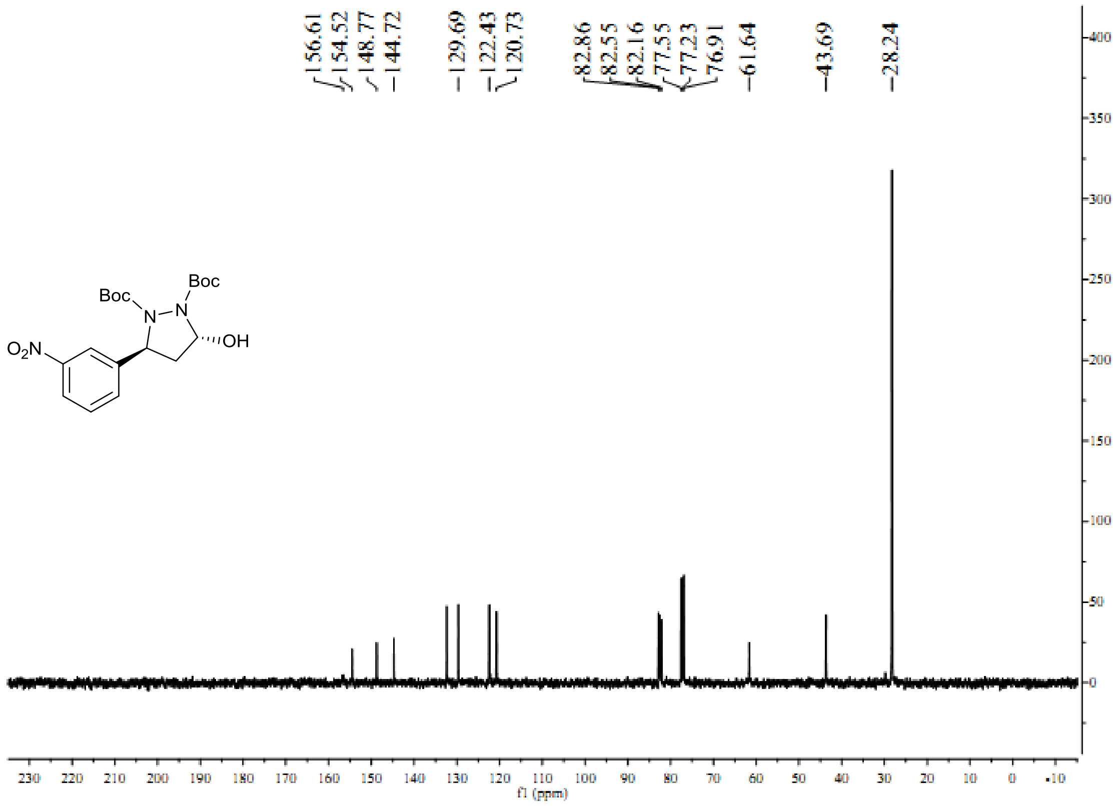

**4c**

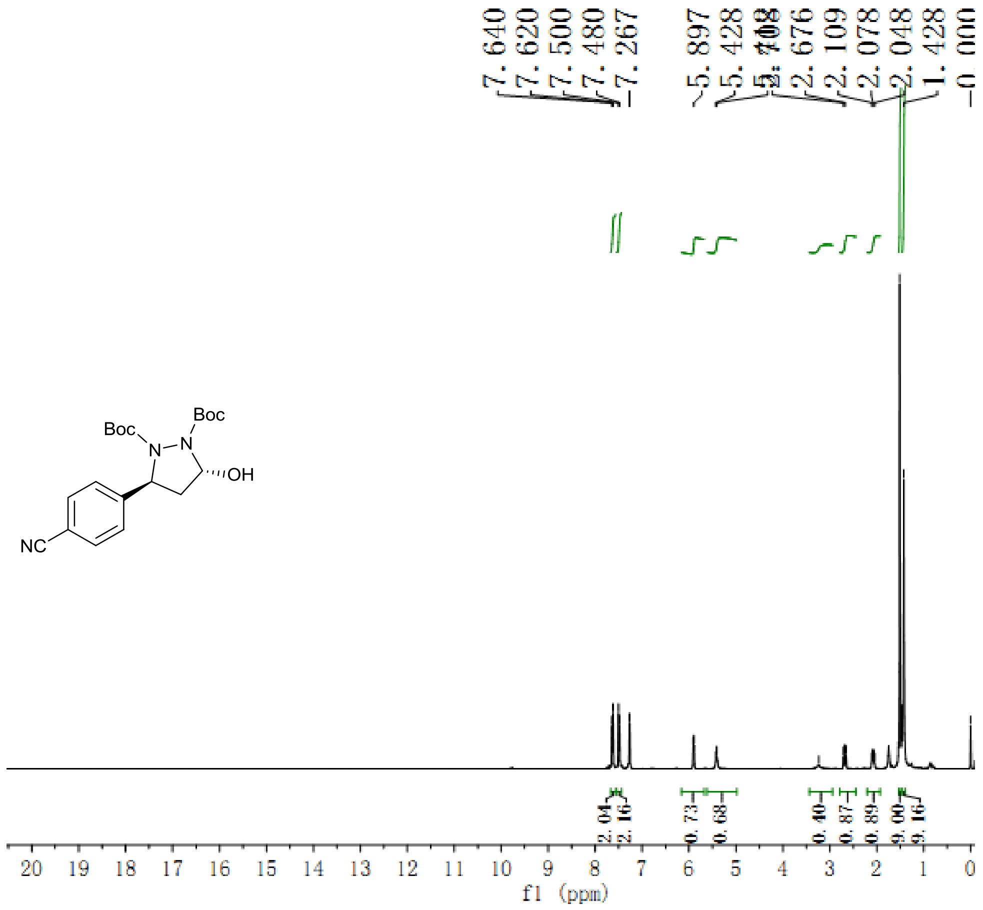

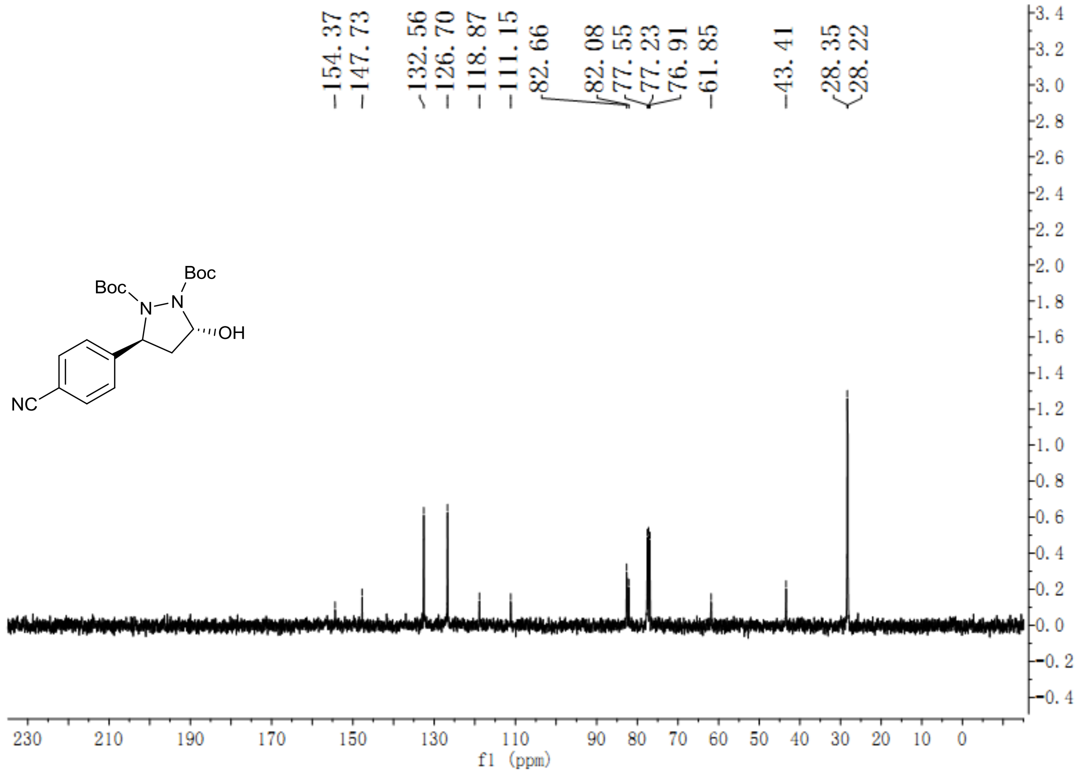

**4d**

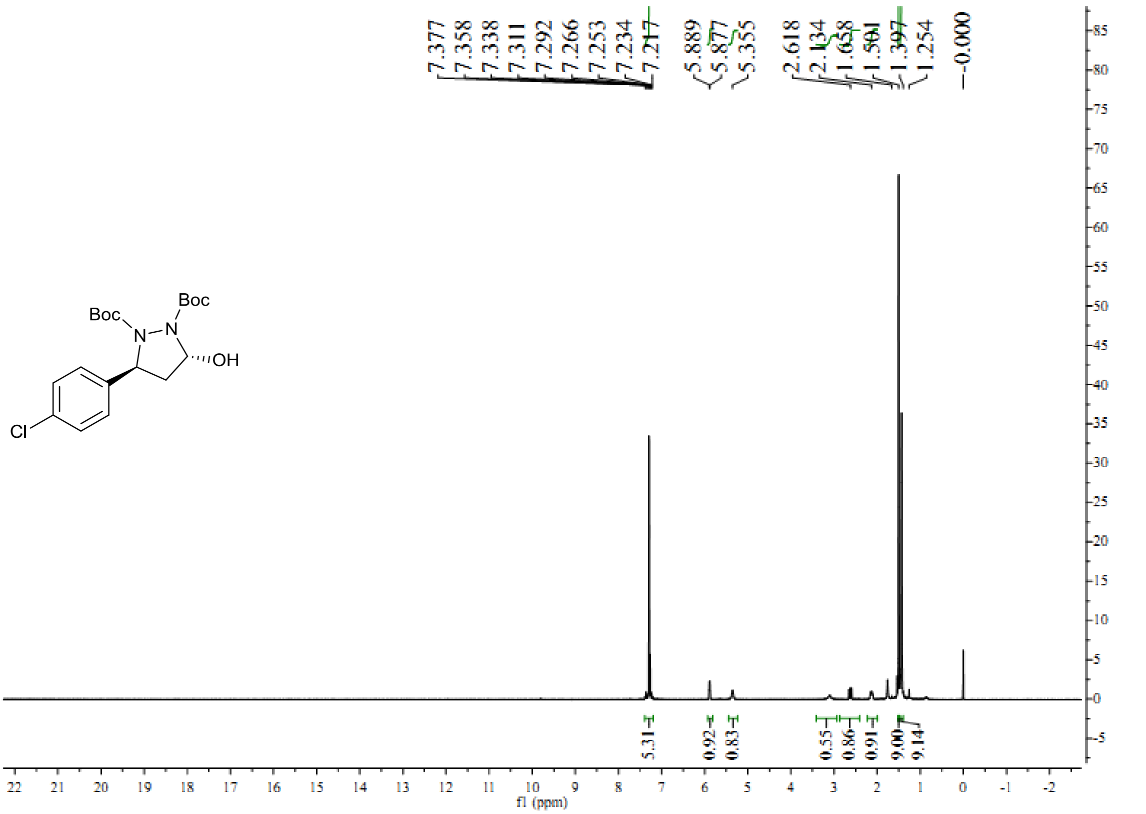

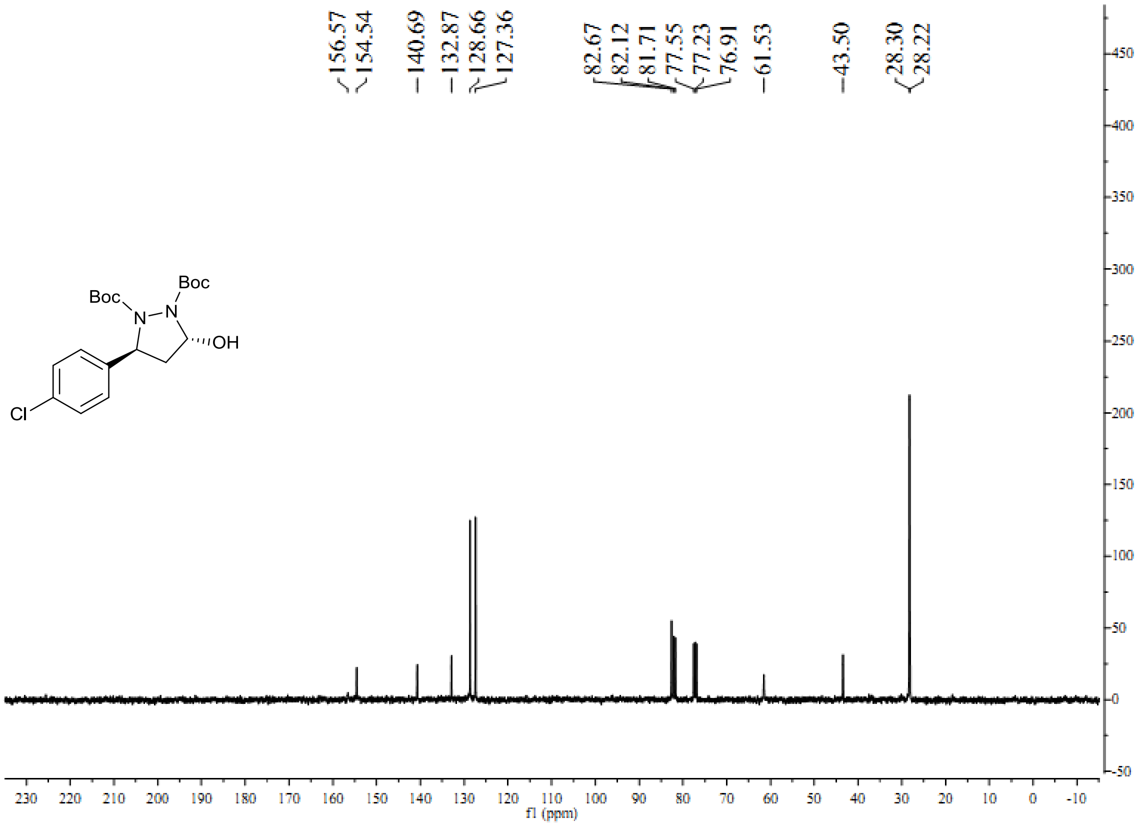

**4e**

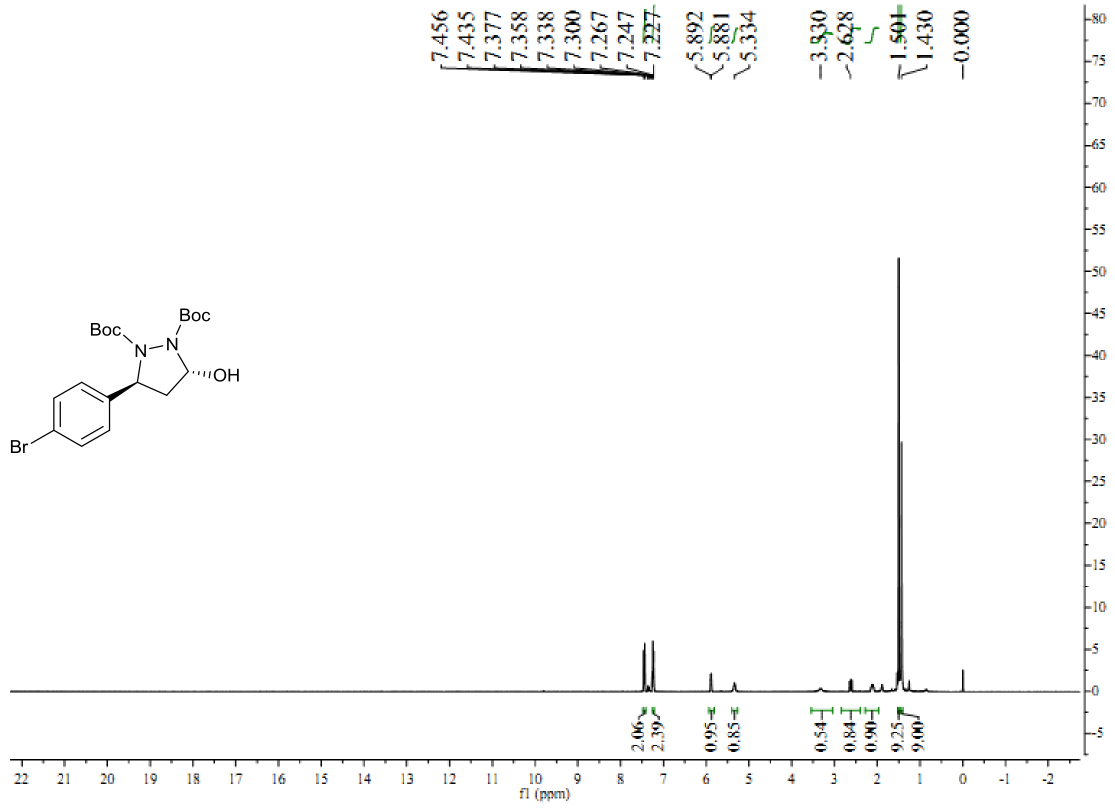

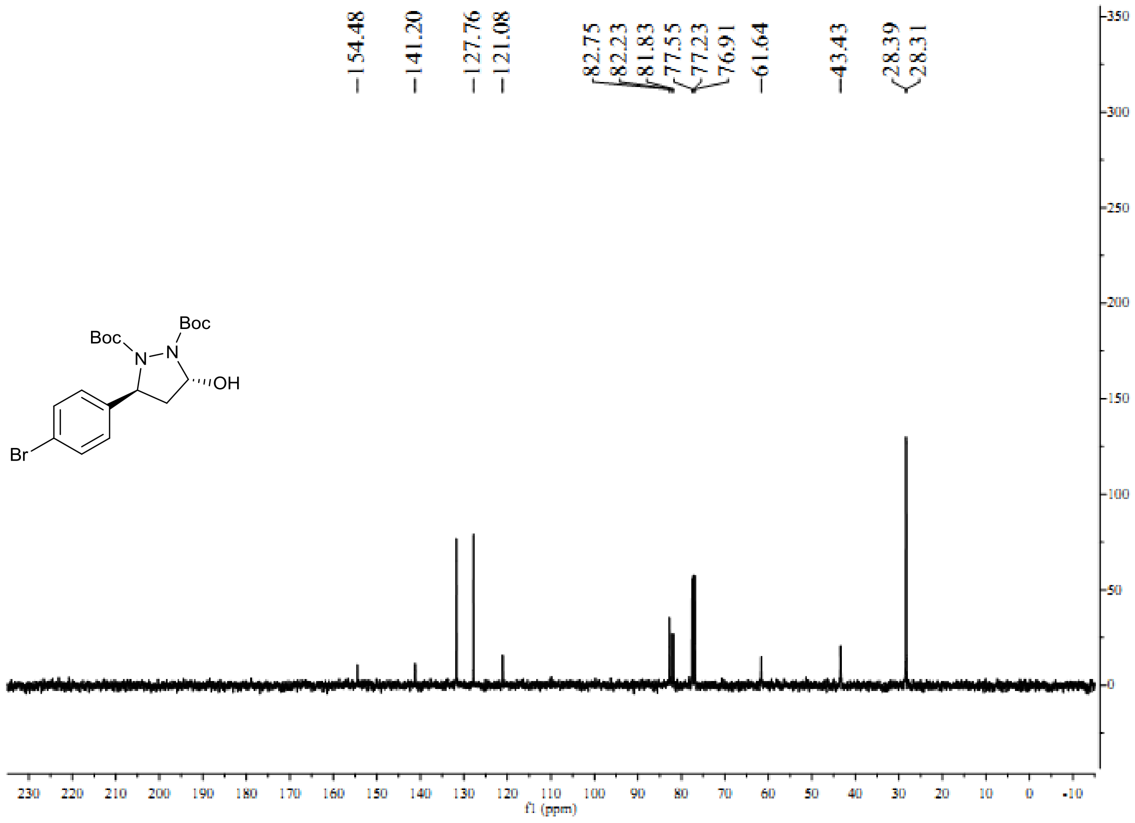

**4h**

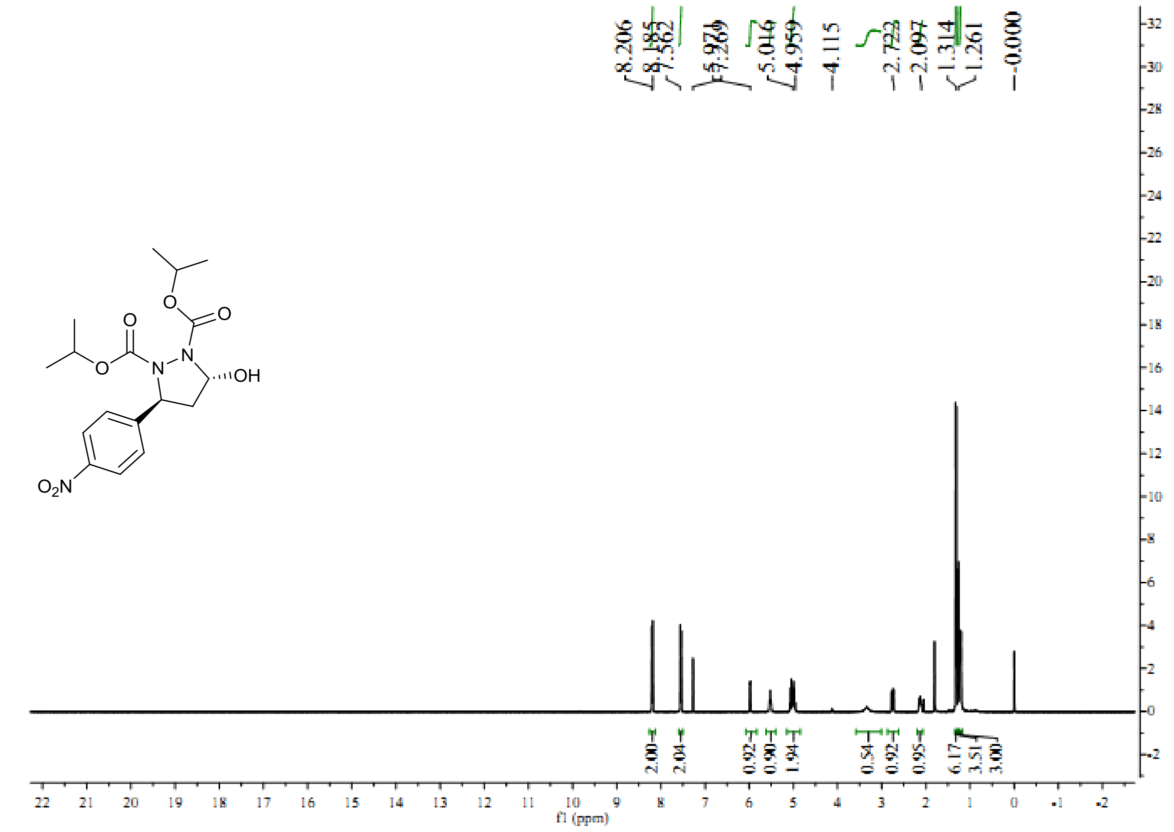
OH
$O_2N$
f1 (ppm)

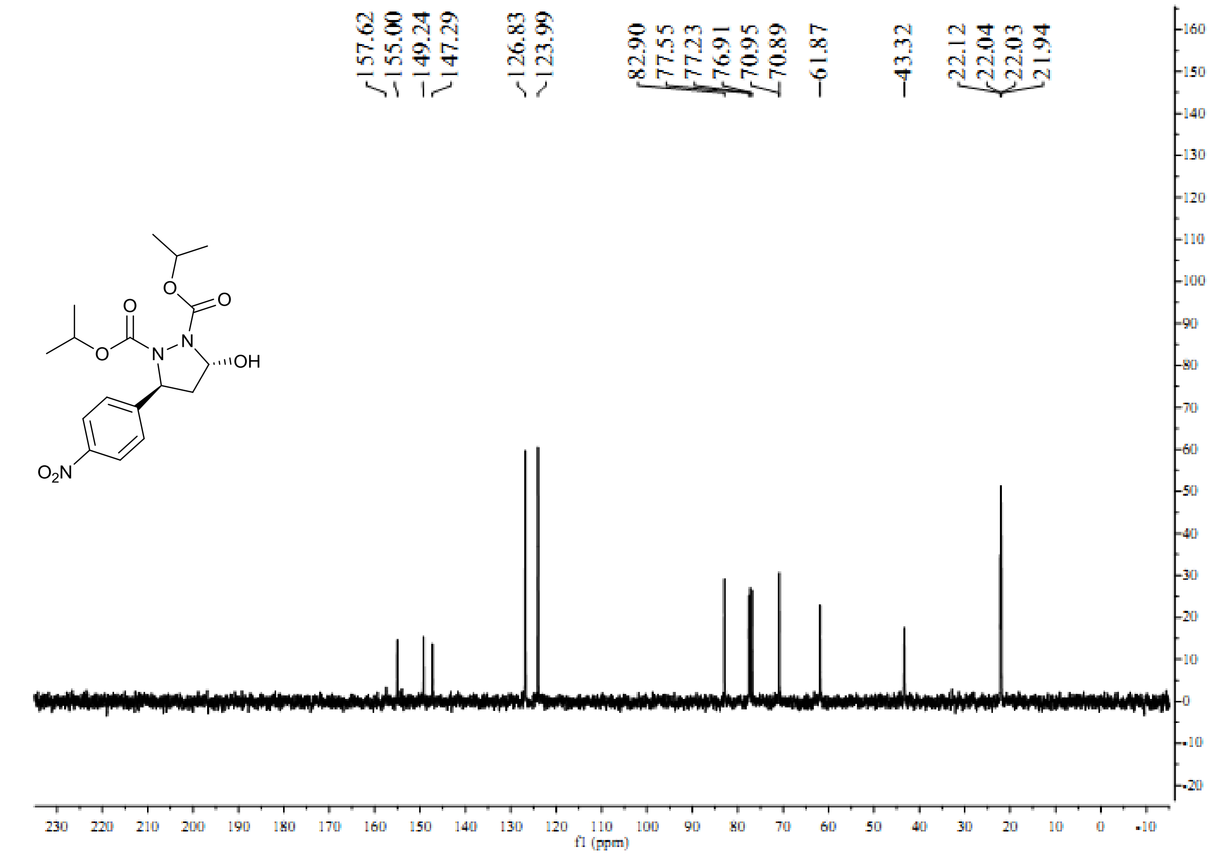
157.62
155.00
149.24
147.29
126.83
123.99
82.90
77.55
77.23
76.91
70.95
70.89
61.87
43.32
22.12
22.04
22.03
21.94
OH
$O_2N$
f1 (ppm)

**4i**

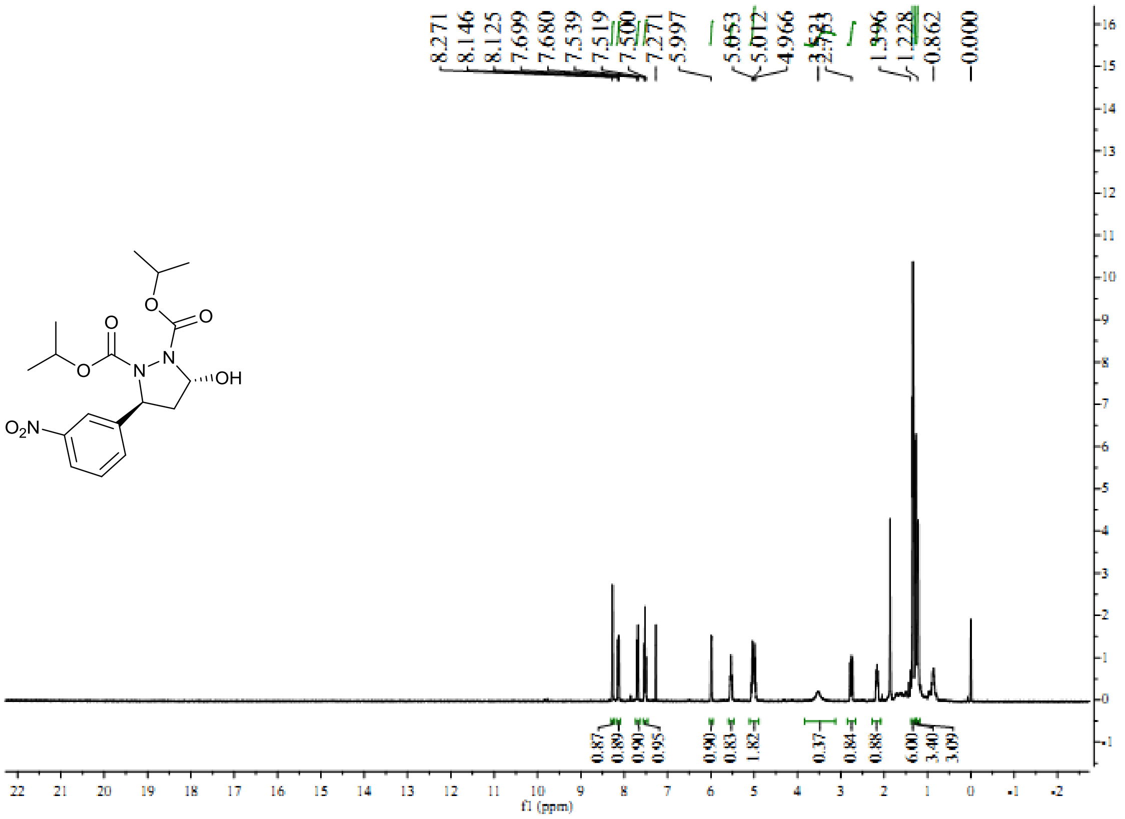

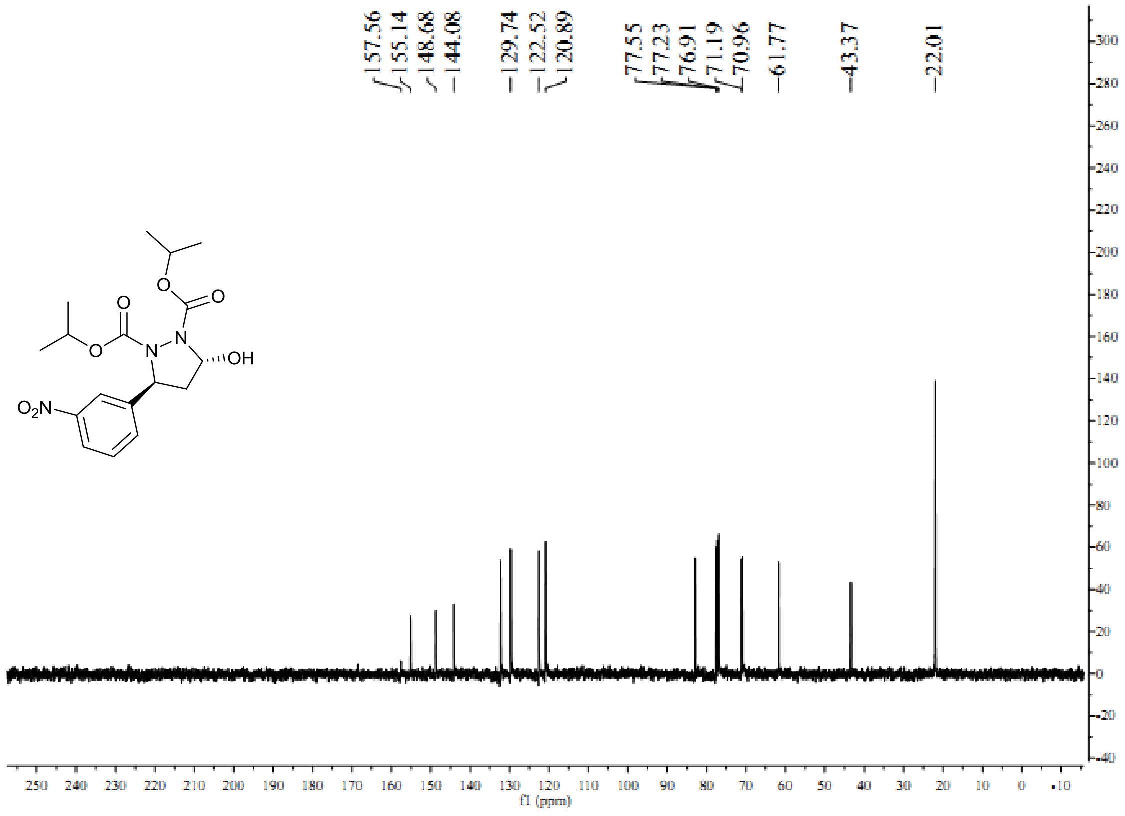

**4j**

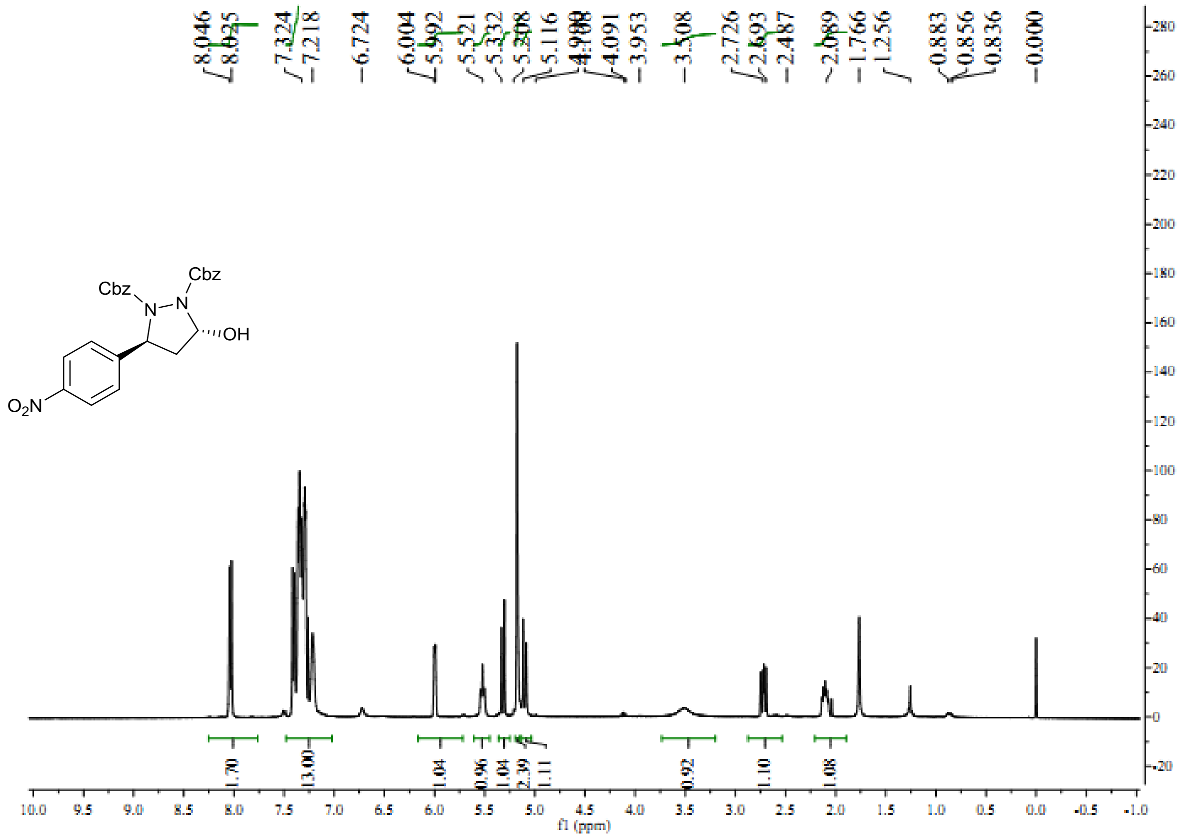

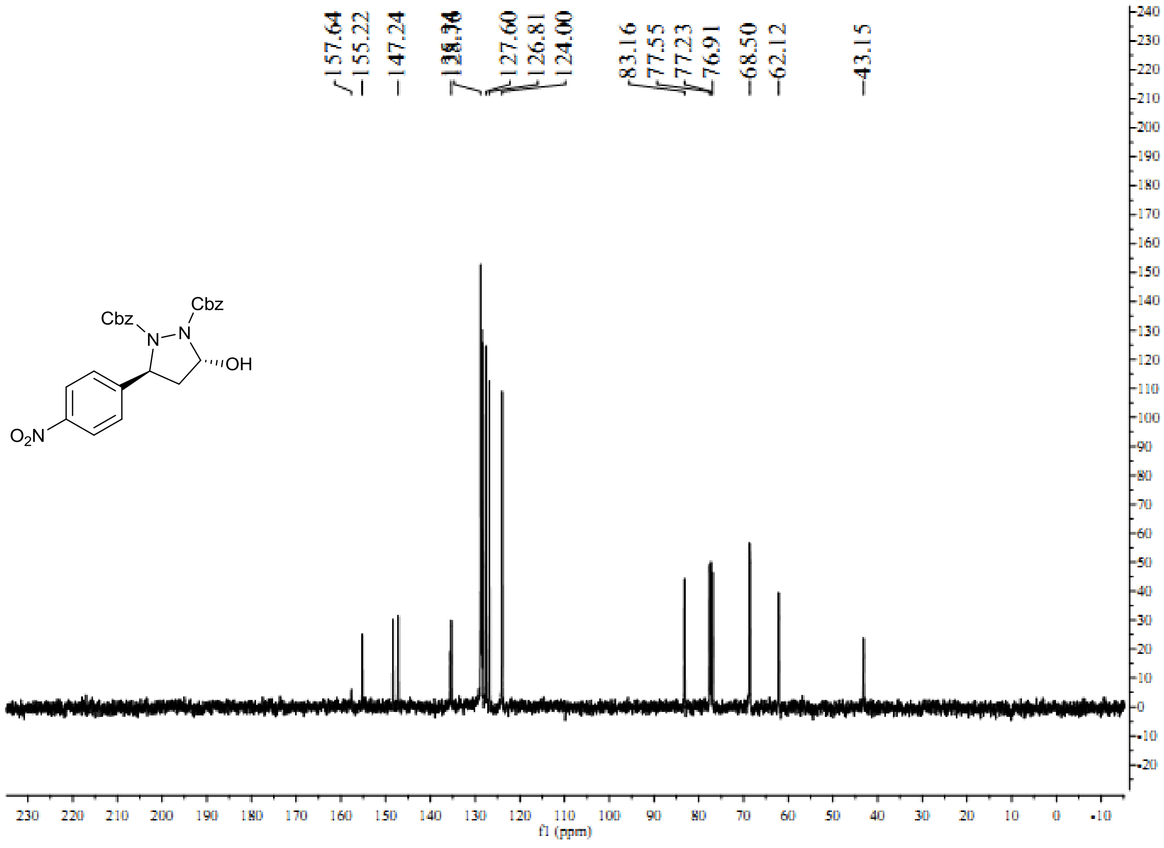

**4k**

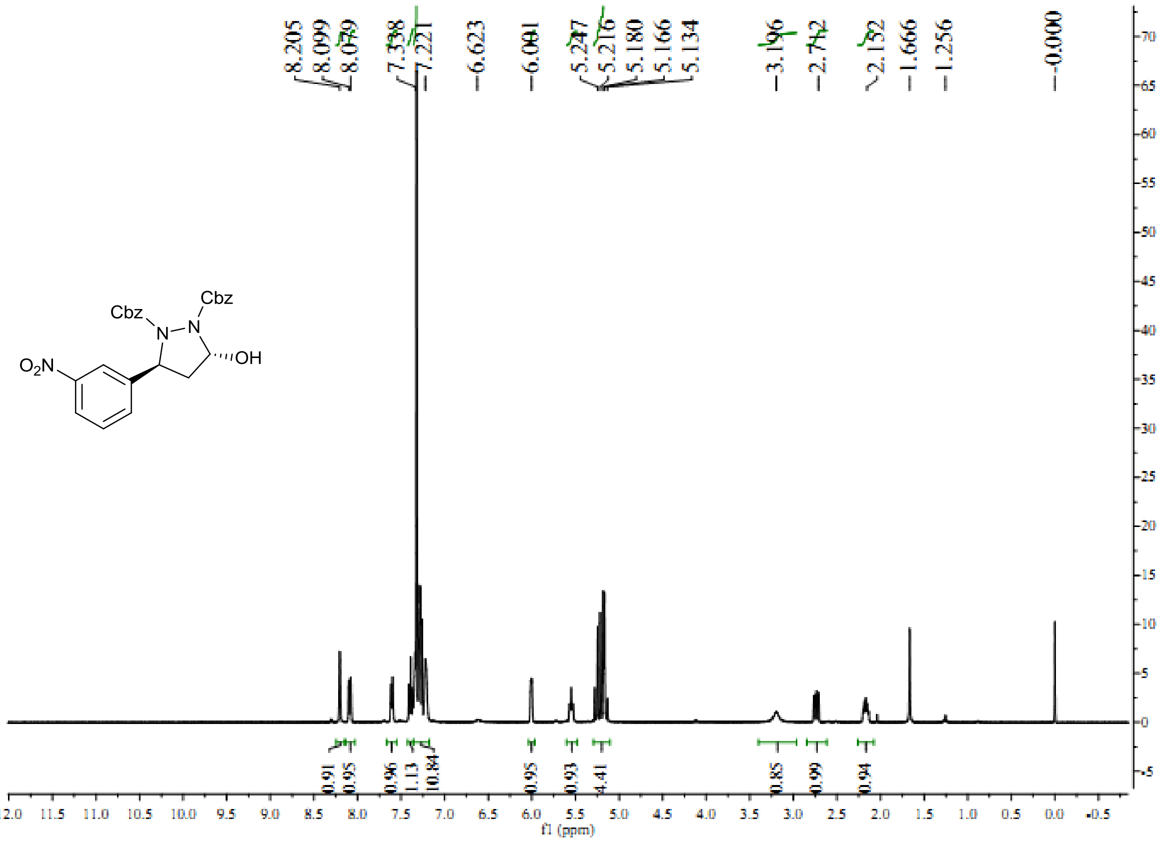

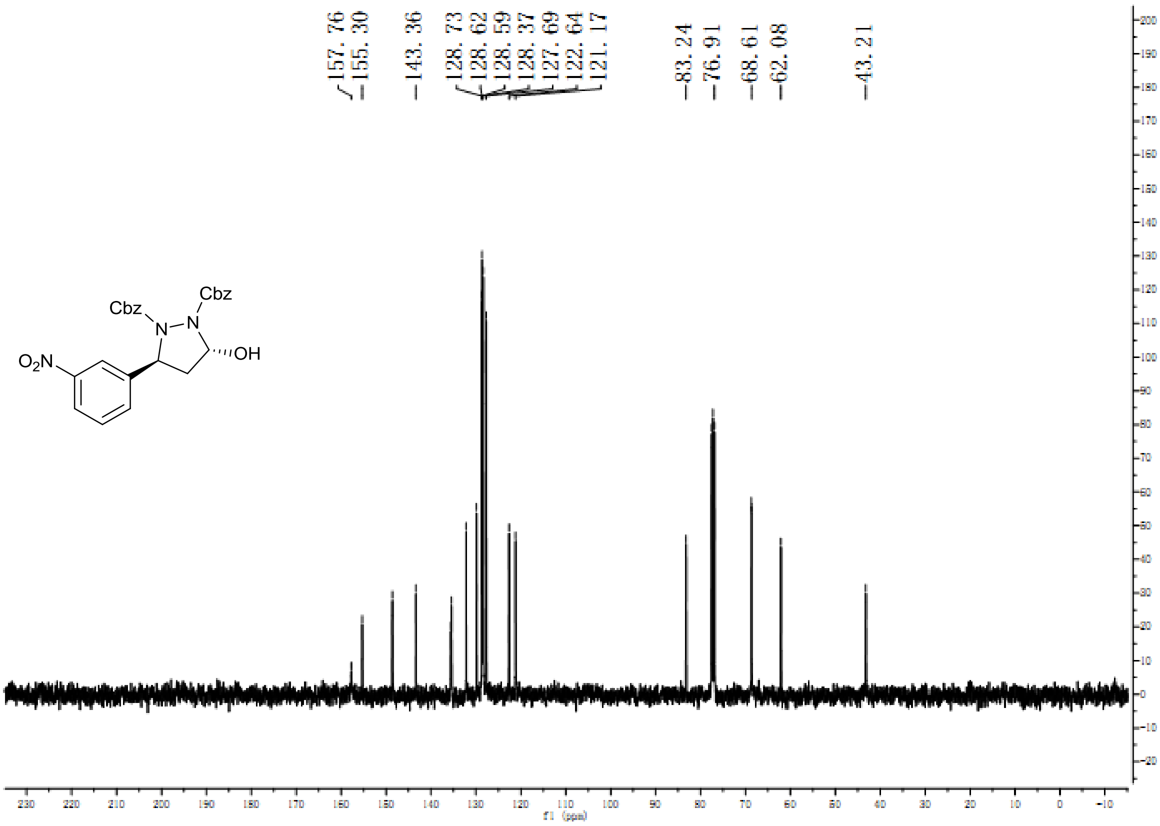

**4l**

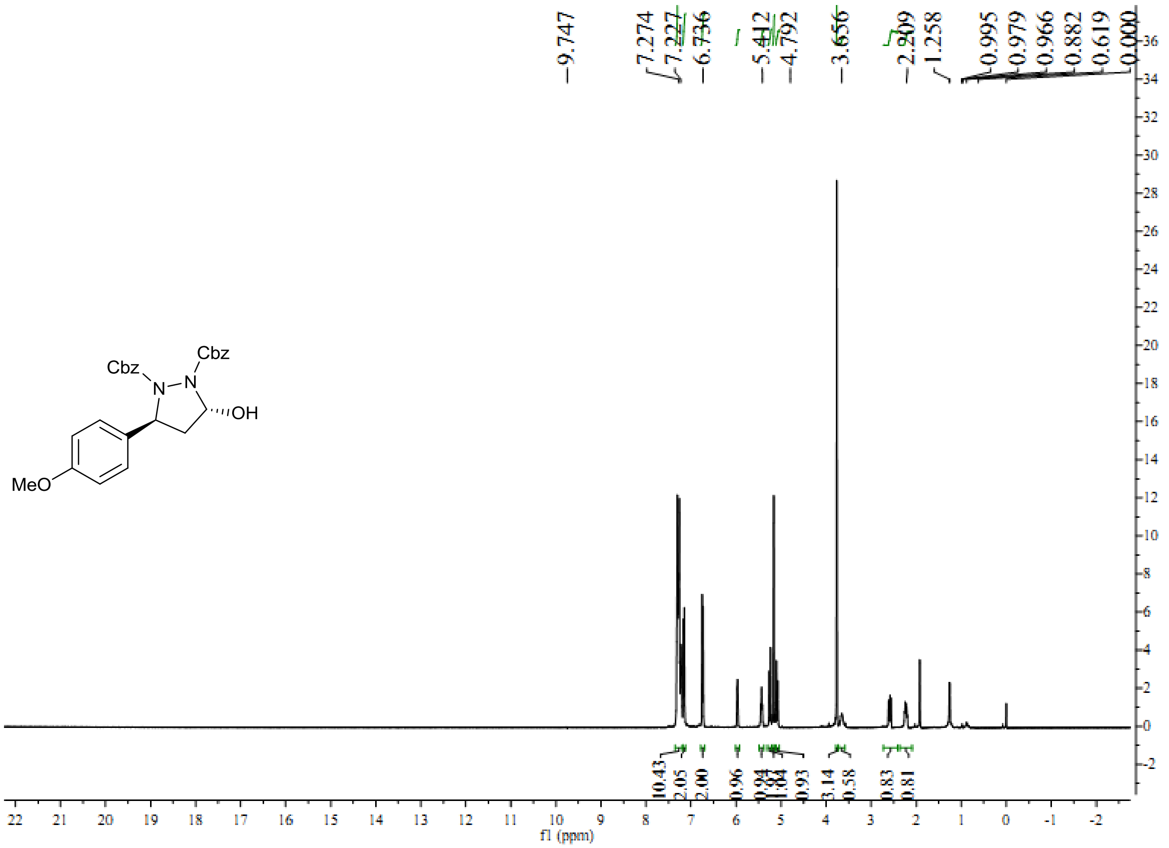

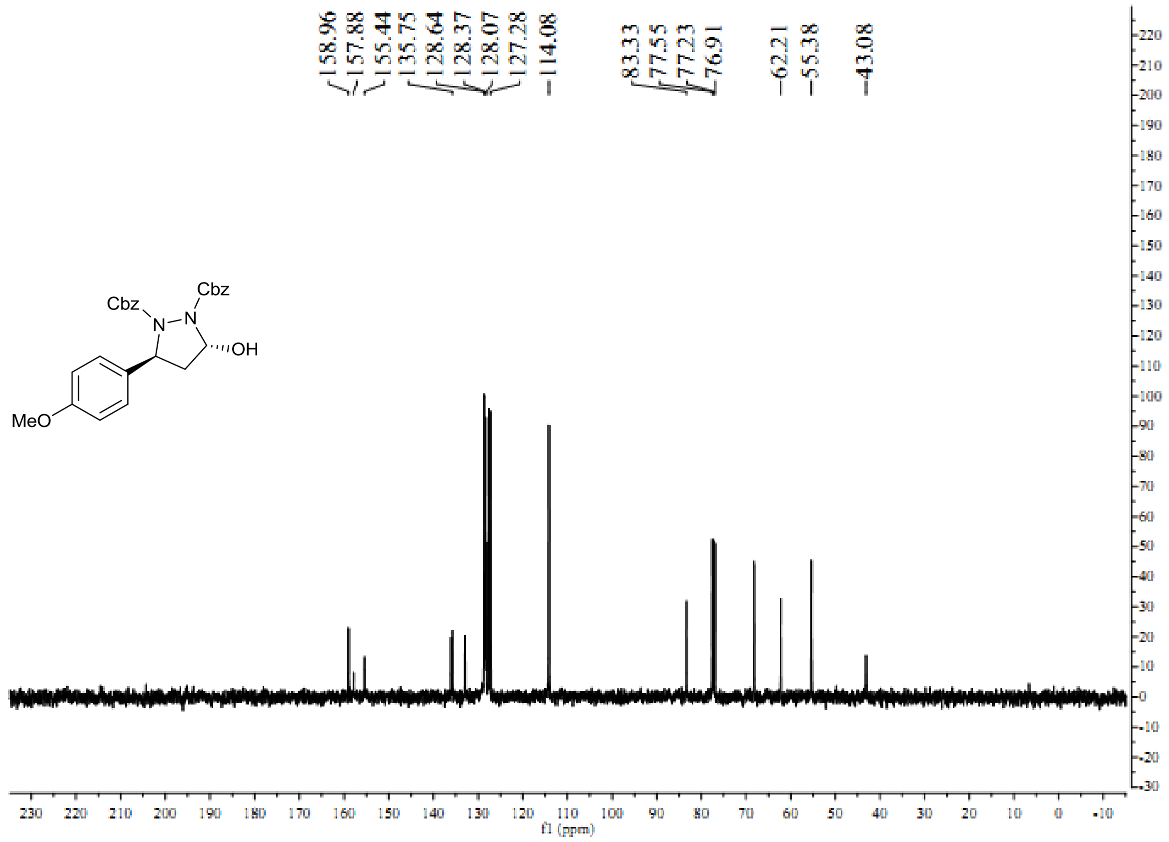

**4m**

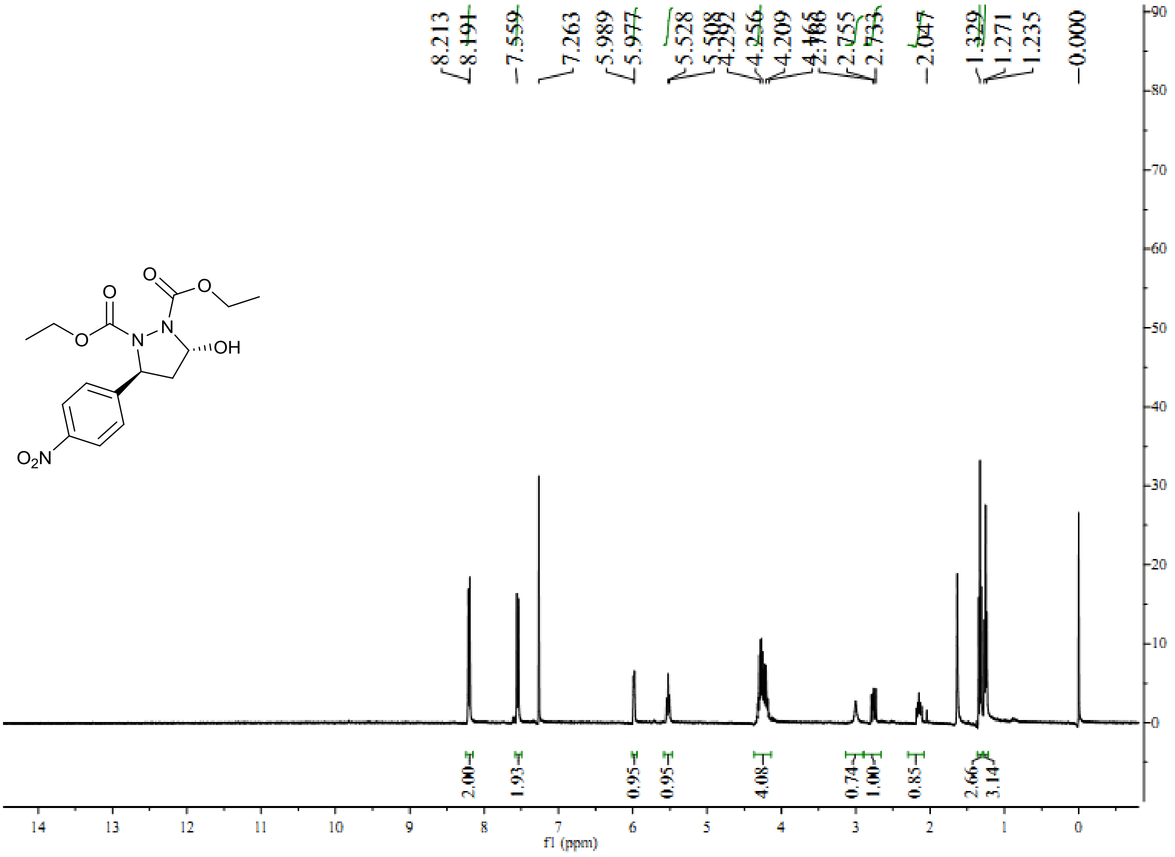

8.213
8.191
7.559
7.263
5.989
5.977
5.528
5.508
4.292
4.256
4.209
4.166
2.755
2.733
2.047
1.329
1.271
1.235
0.000
2.00
1.93
0.95
0.95
4.08
0.74
1.00
0.85
2.66
3.14
f1 (ppm)
O
O
N
N
OH
O
O
O2N


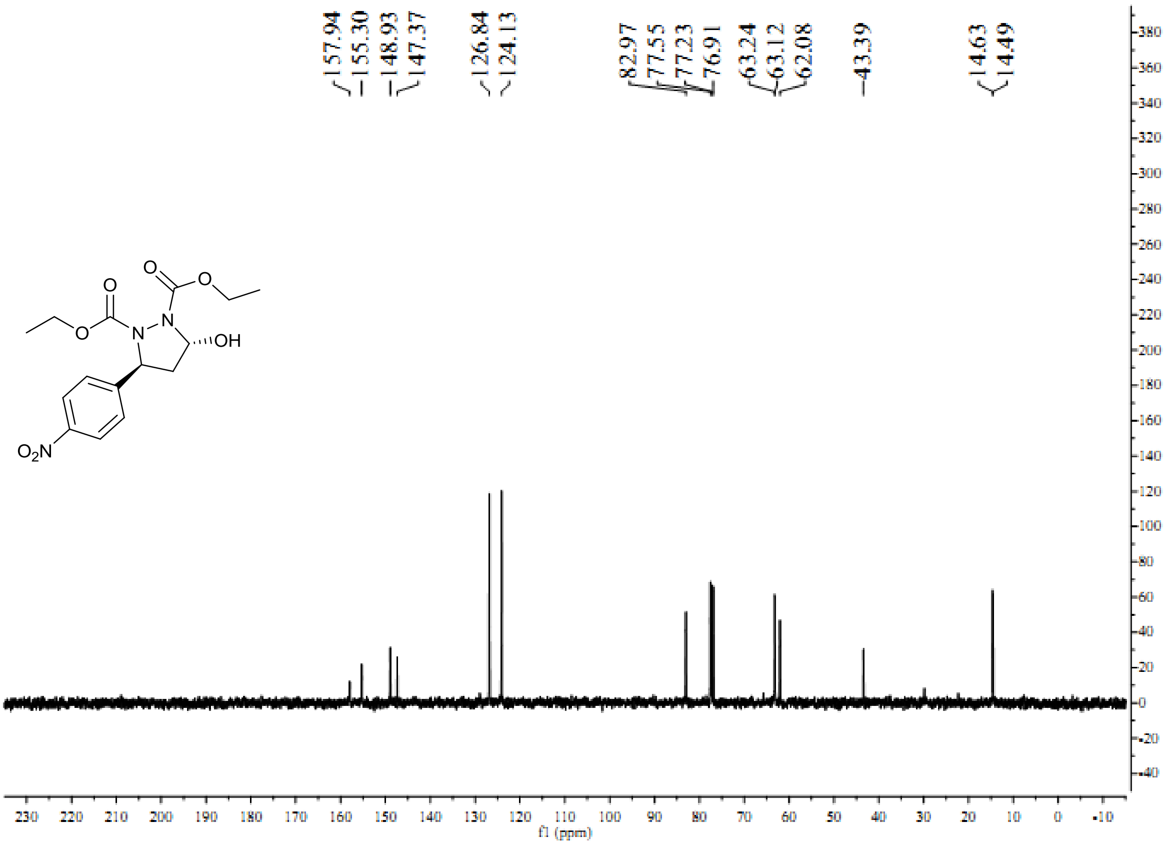

157.94
155.30
148.93
147.37
126.84
124.13
82.97
77.55
77.23
76.91
63.24
63.12
62.08
43.39
14.63
14.49
f1 (ppm)
O
O
N
N
OH
O
O
O2N

**4n**

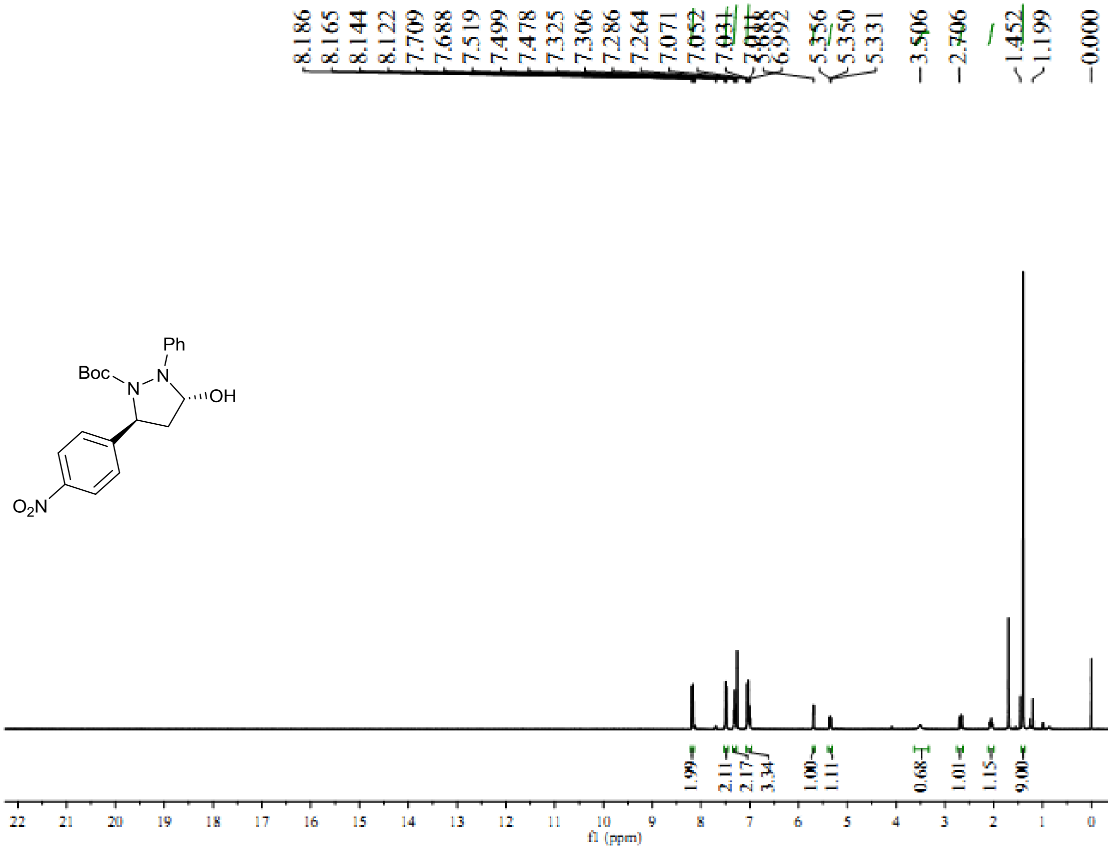

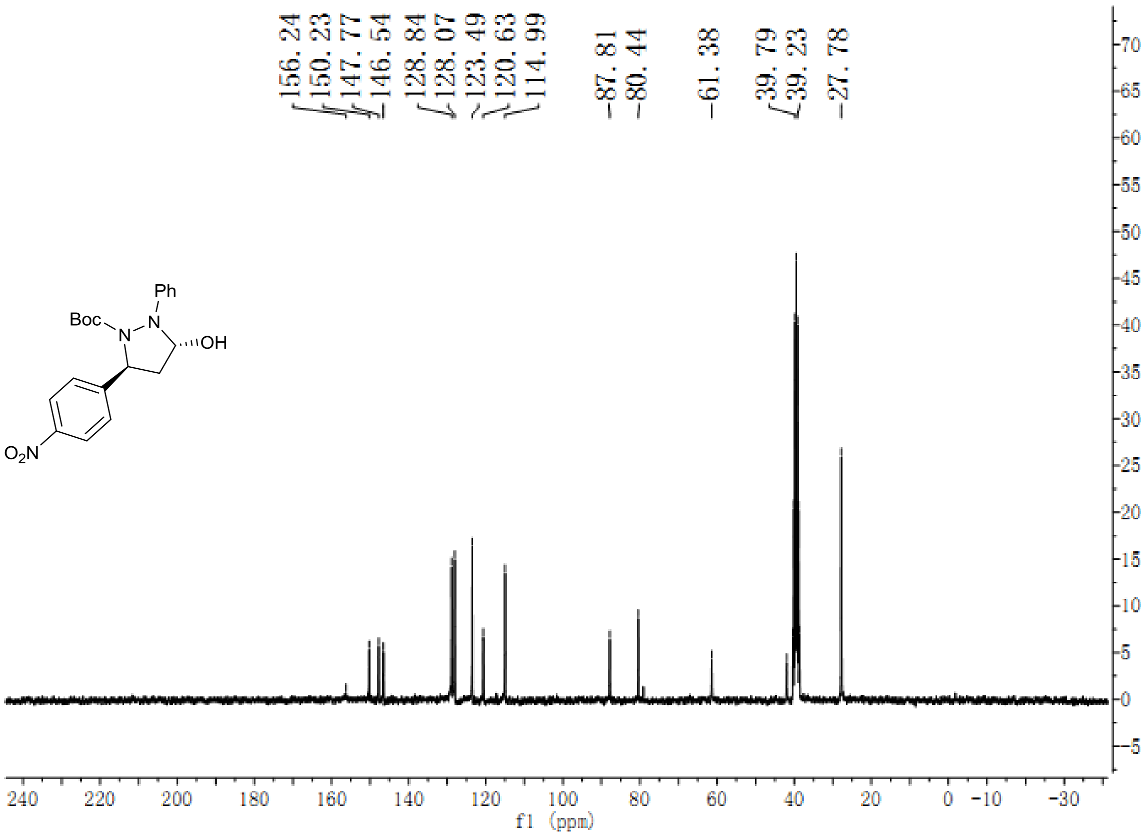

**4n'**

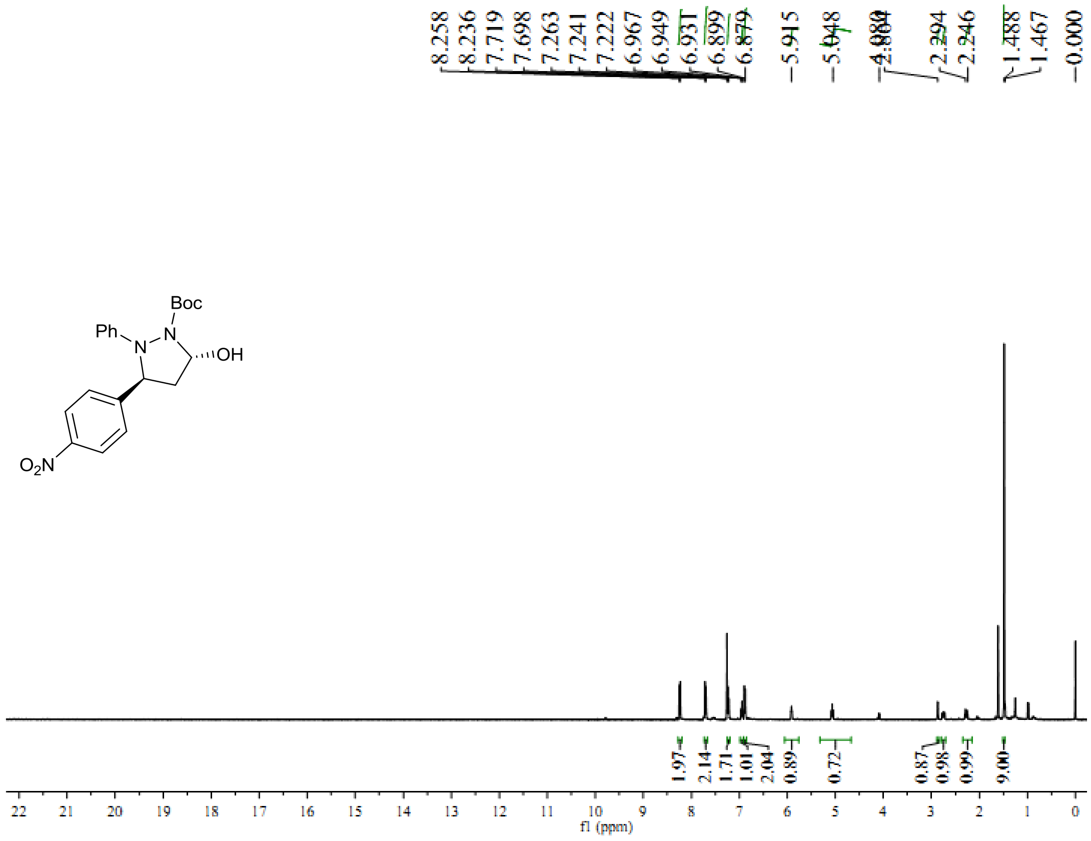

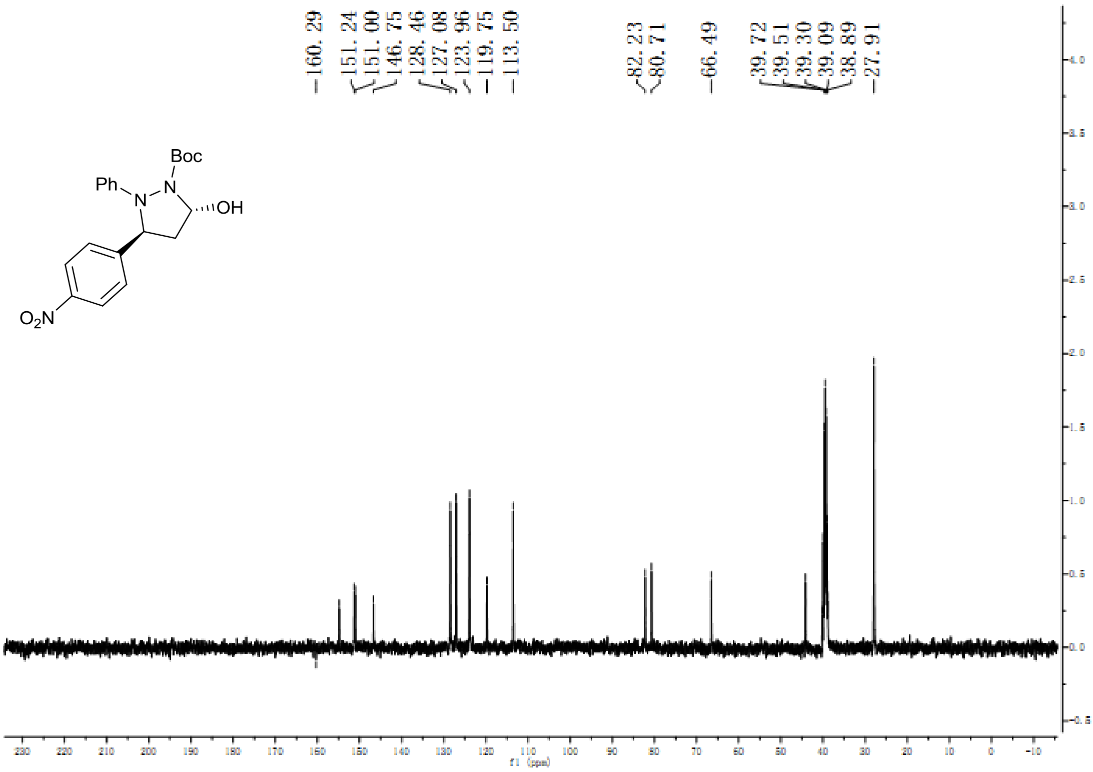

**4o**

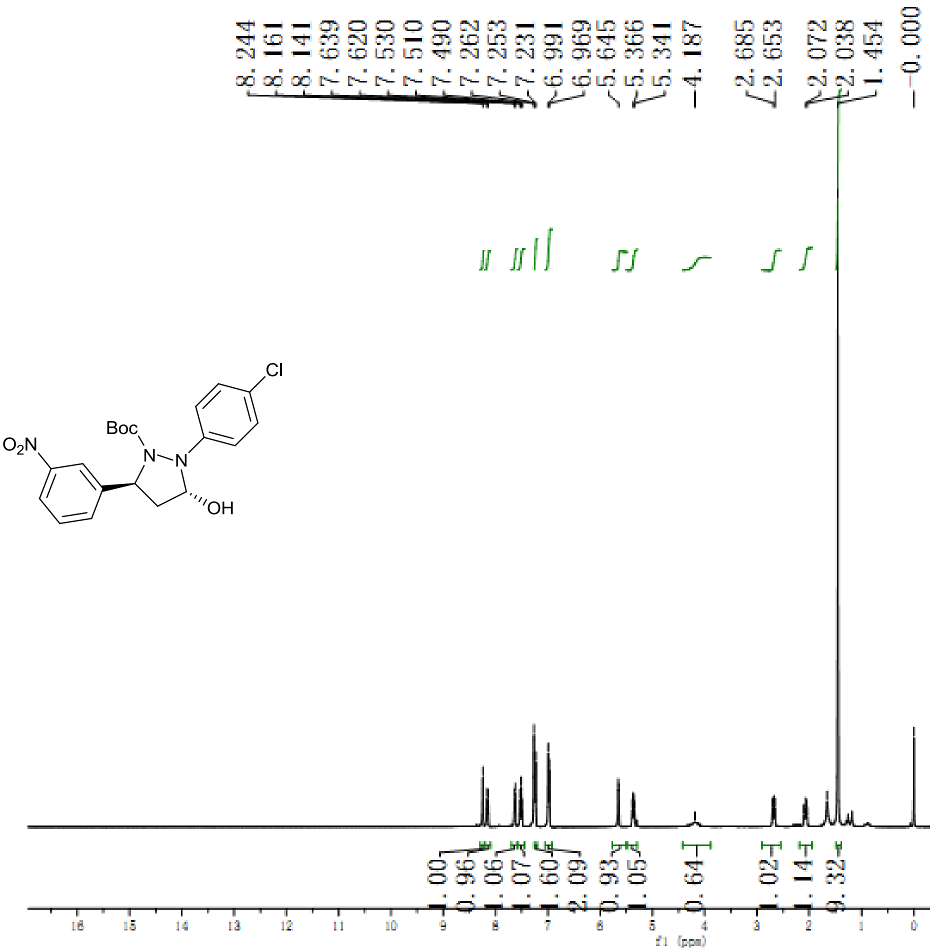


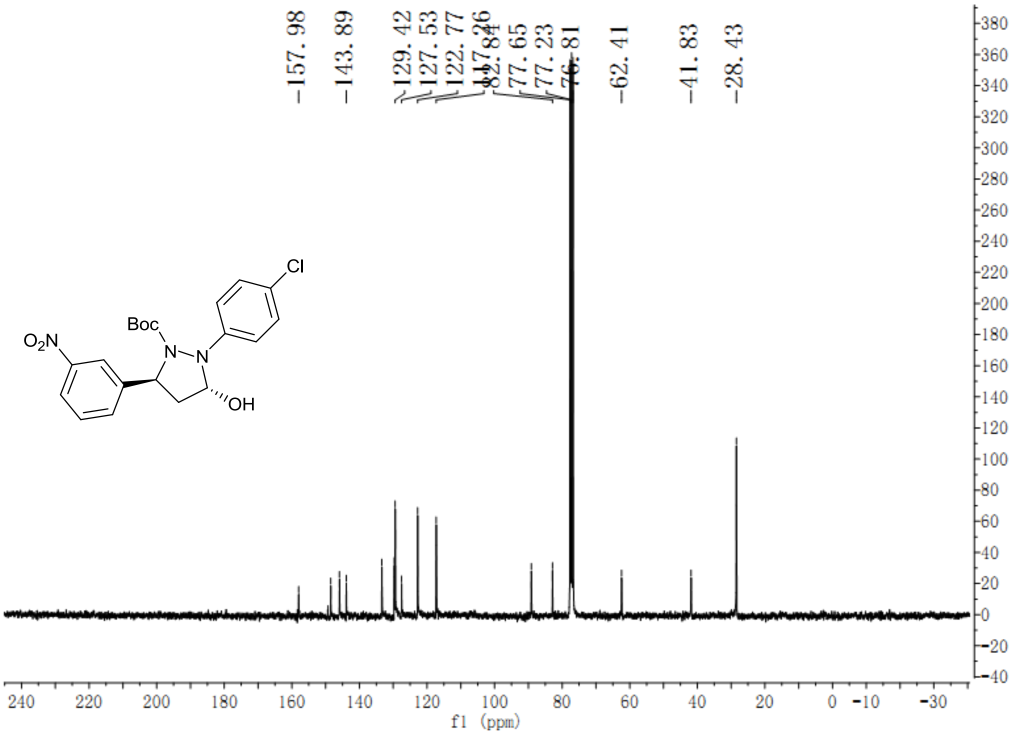

**4p**

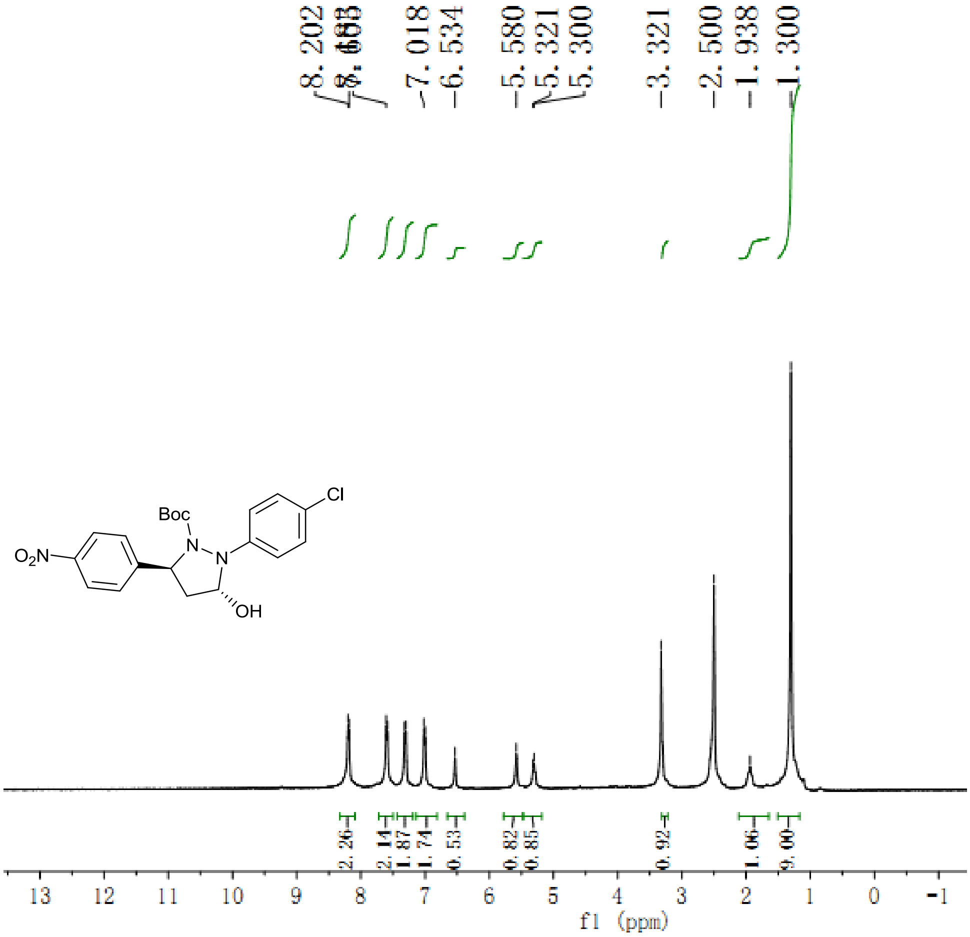

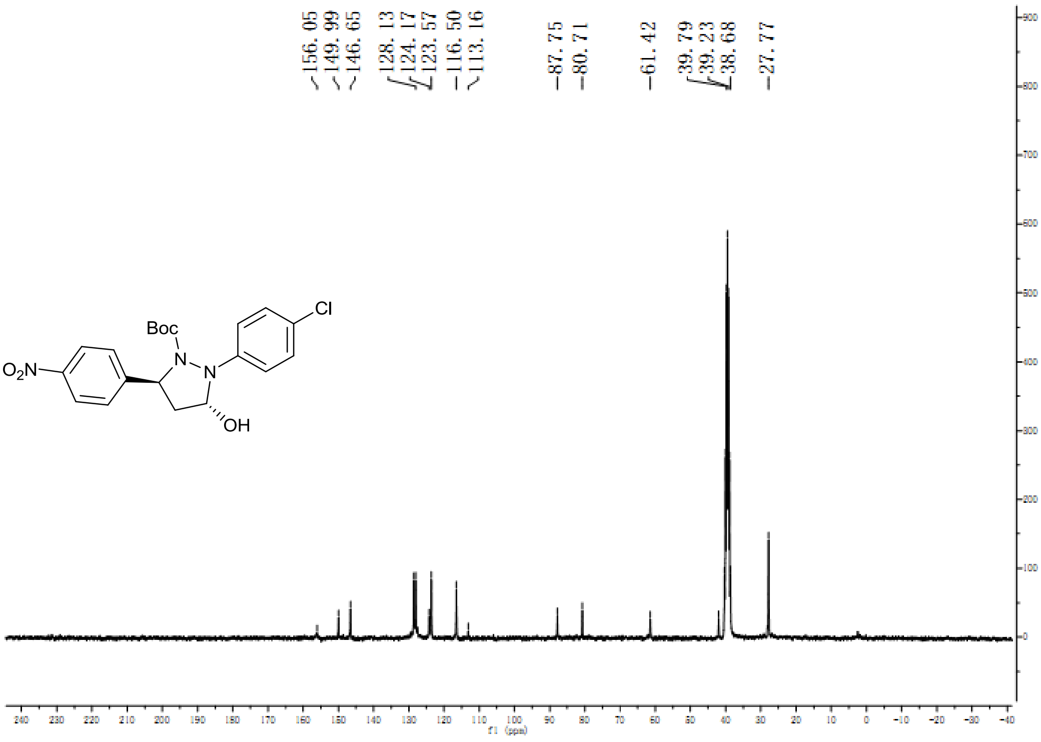

**4q**

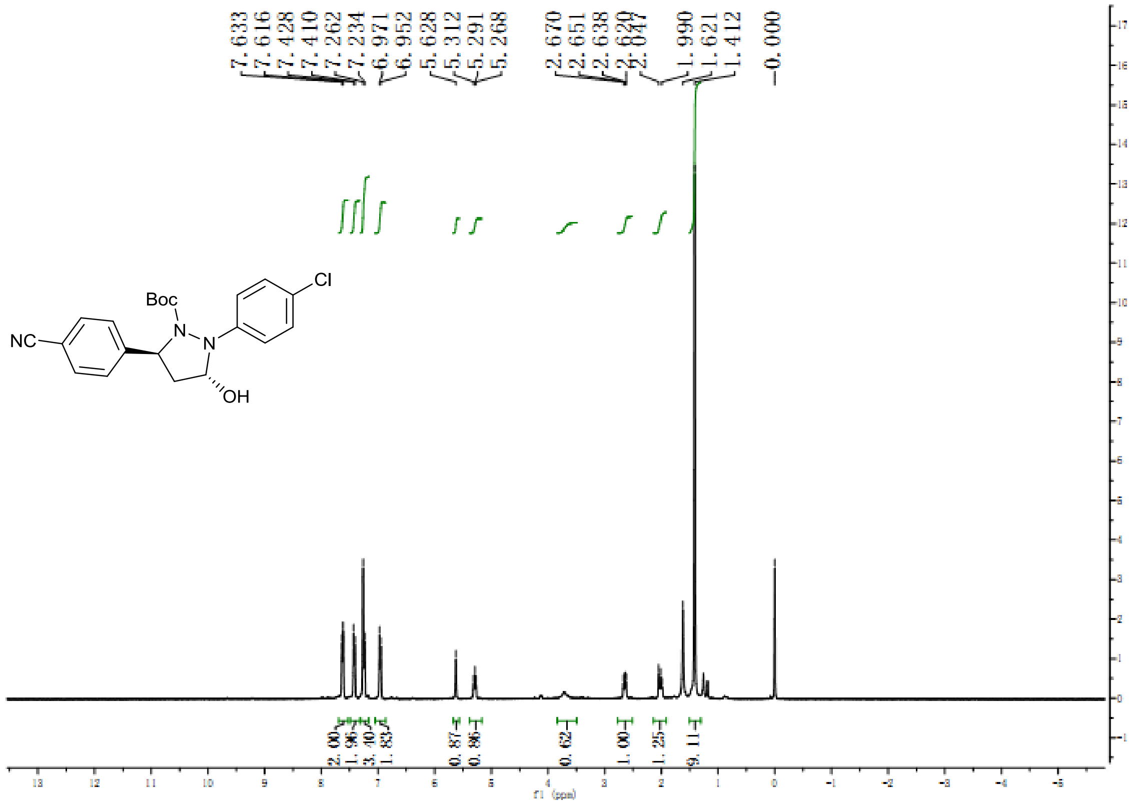

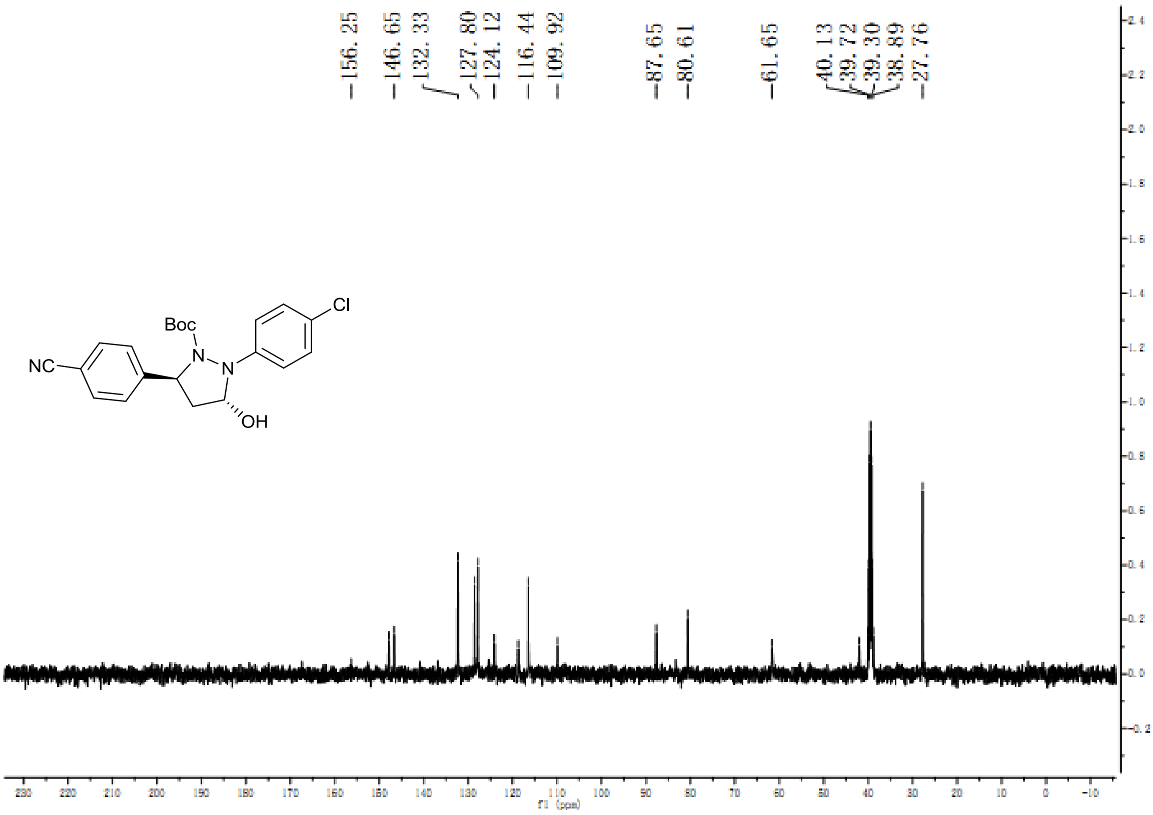

**4r**

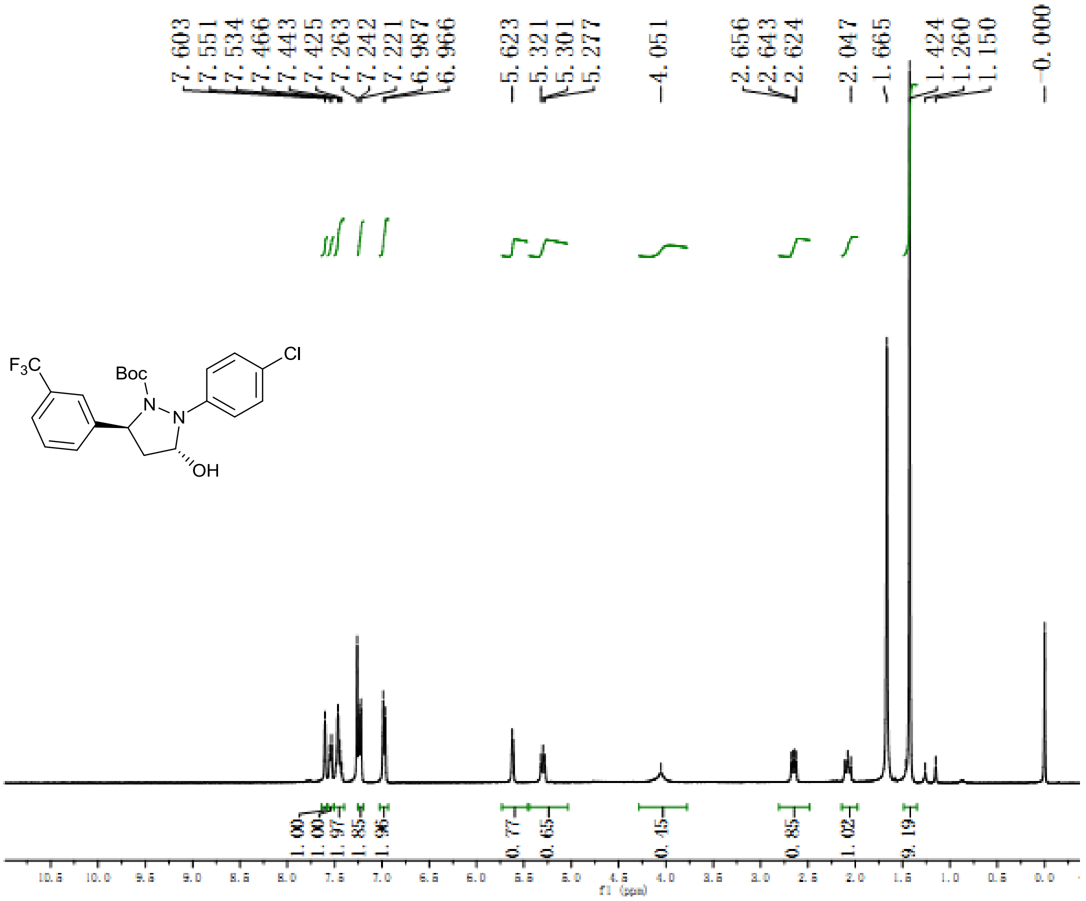

7.603
7.551
7.534
7.466
7.443
7.425
7.263
7.242
7.221
6.987
6.966
5.623
5.321
5.301
5.277
4.051
2.656
2.643
2.624
2.047
1.665
1.424
1.260
1.150
0.000
F3C
Boc
N
N
Cl
OH
f1 (ppm)


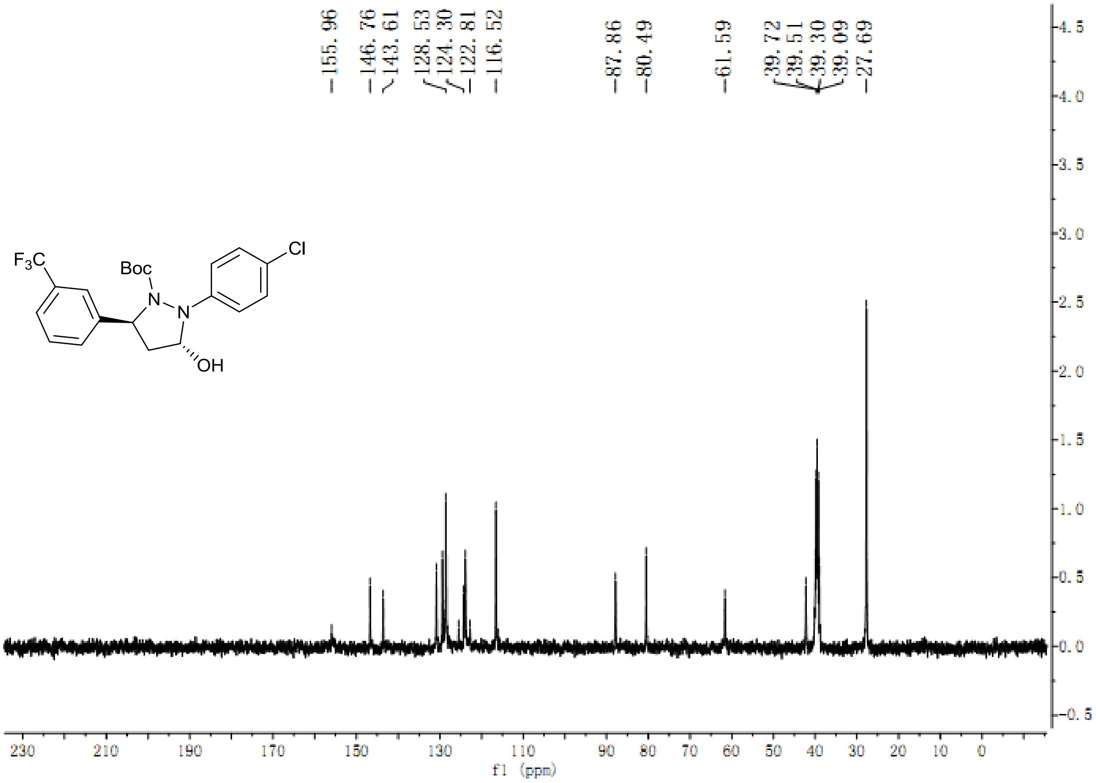

155.96
146.76
143.61
128.53
124.30
122.81
116.52
87.86
80.49
61.59
39.72
39.51
39.30
39.09
27.69
F3C
Boc
N
N
Cl
OH
f1 (ppm)

**4s**

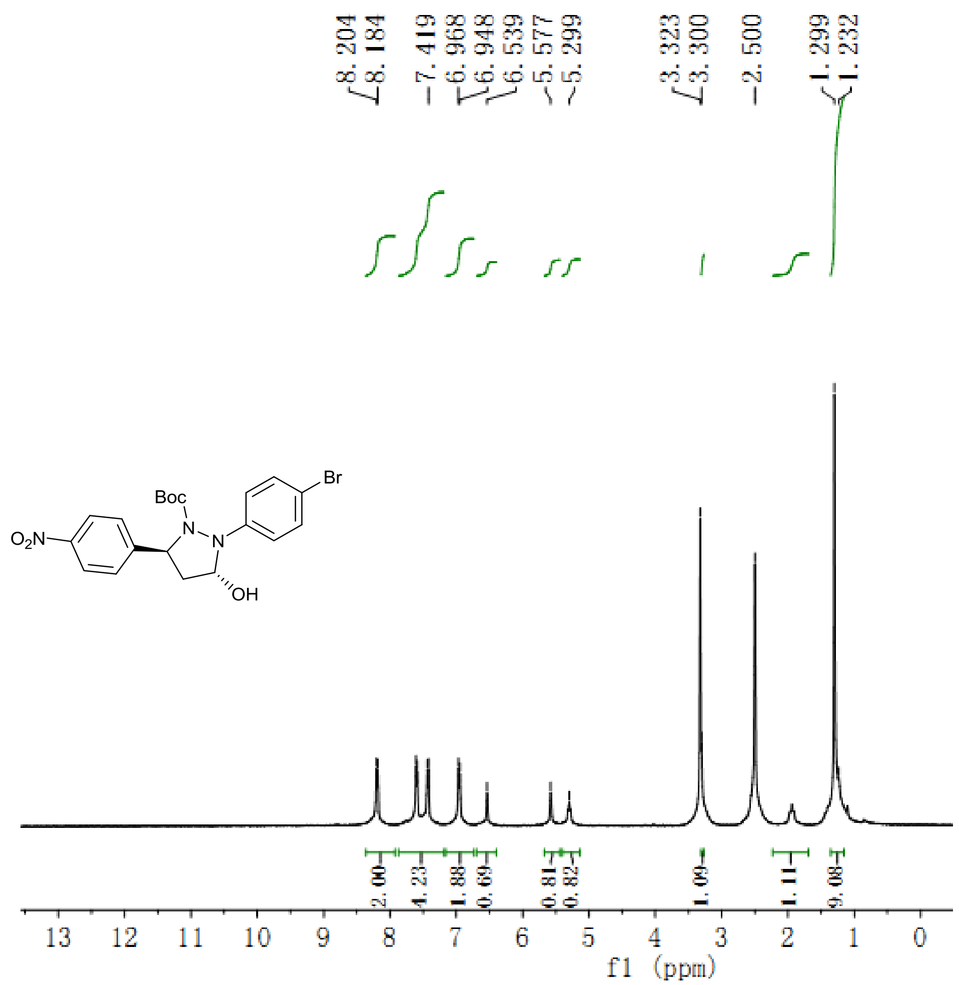

8.204
8.184
7.419
6.968
6.948
6.539
5.577
5.299
3.323
3.300
2.500
1.299
1.232
Br
Boc
N
N
O2N
OH
2.00
4.23
1.88
0.69
0.81
0.82
1.00
1.11
9.08
f1 (ppm)


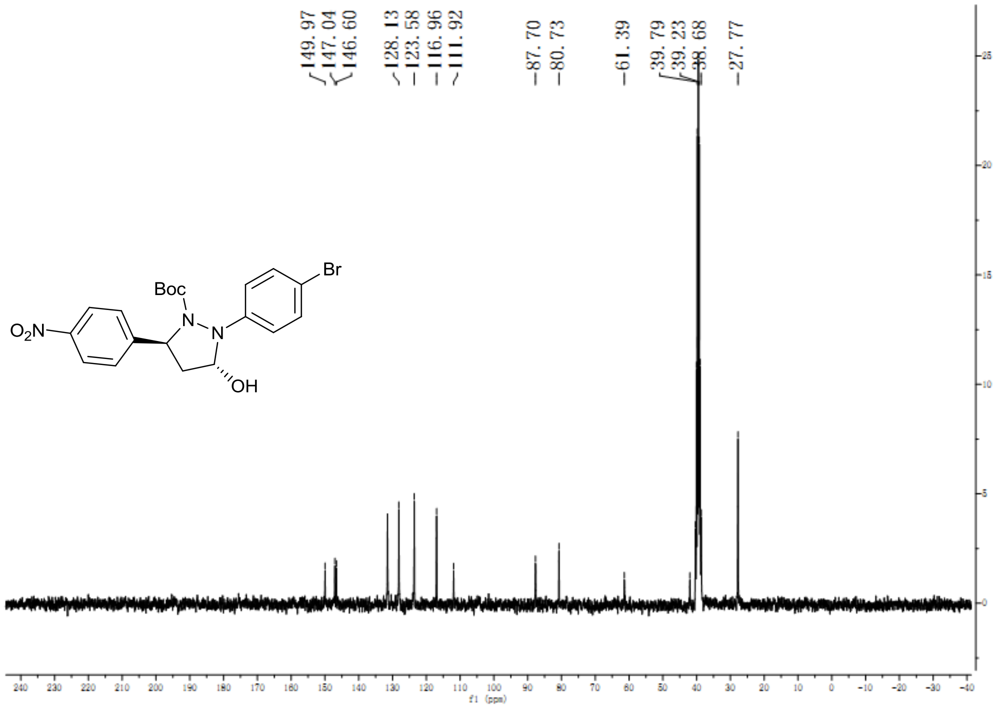

149.97
147.04
146.60
128.13
123.58
116.96
111.92
87.70
80.73
61.39
39.79
39.23
38.68
27.77
Br
Boc
N
N
O2N
OH

**4t**

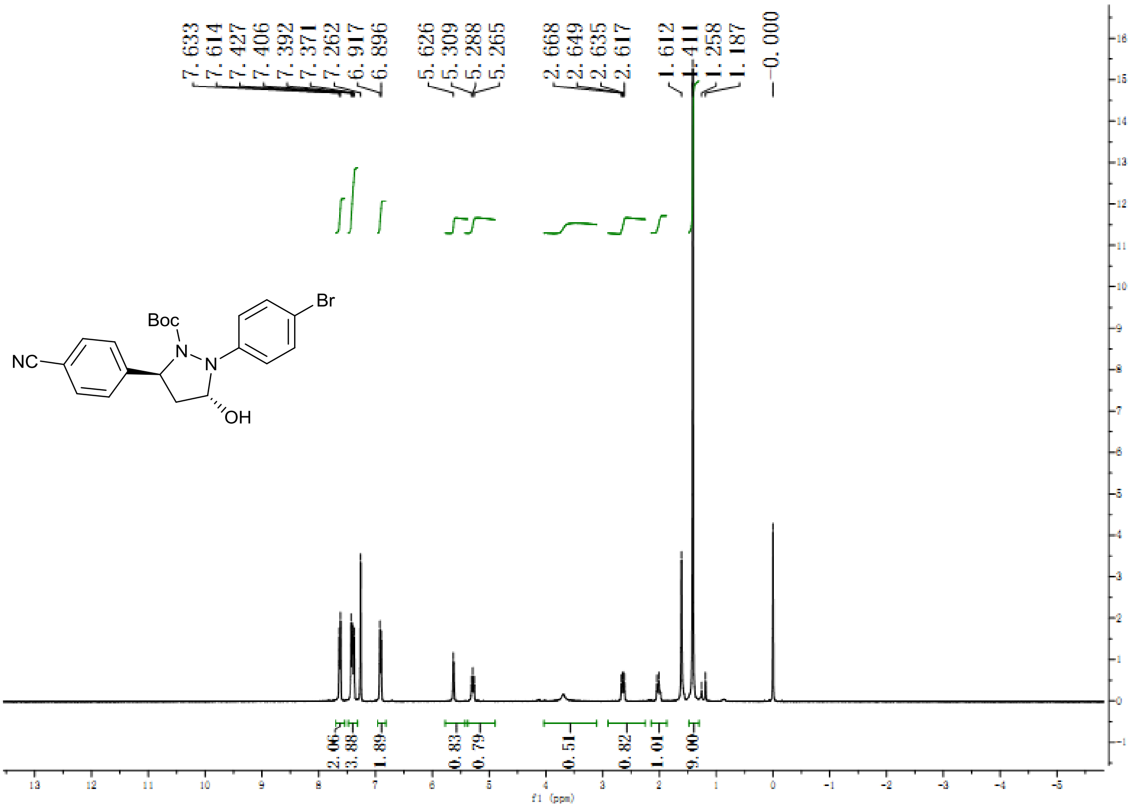

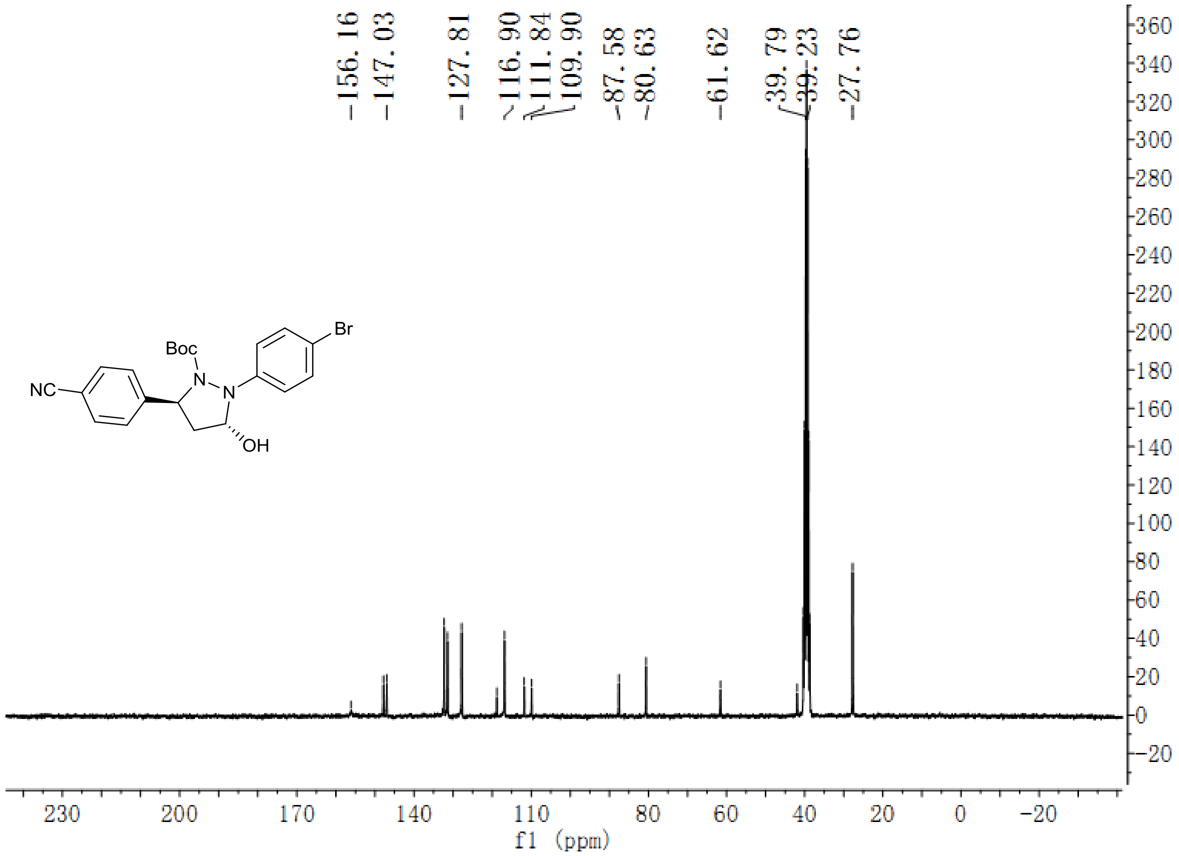

**4u’**

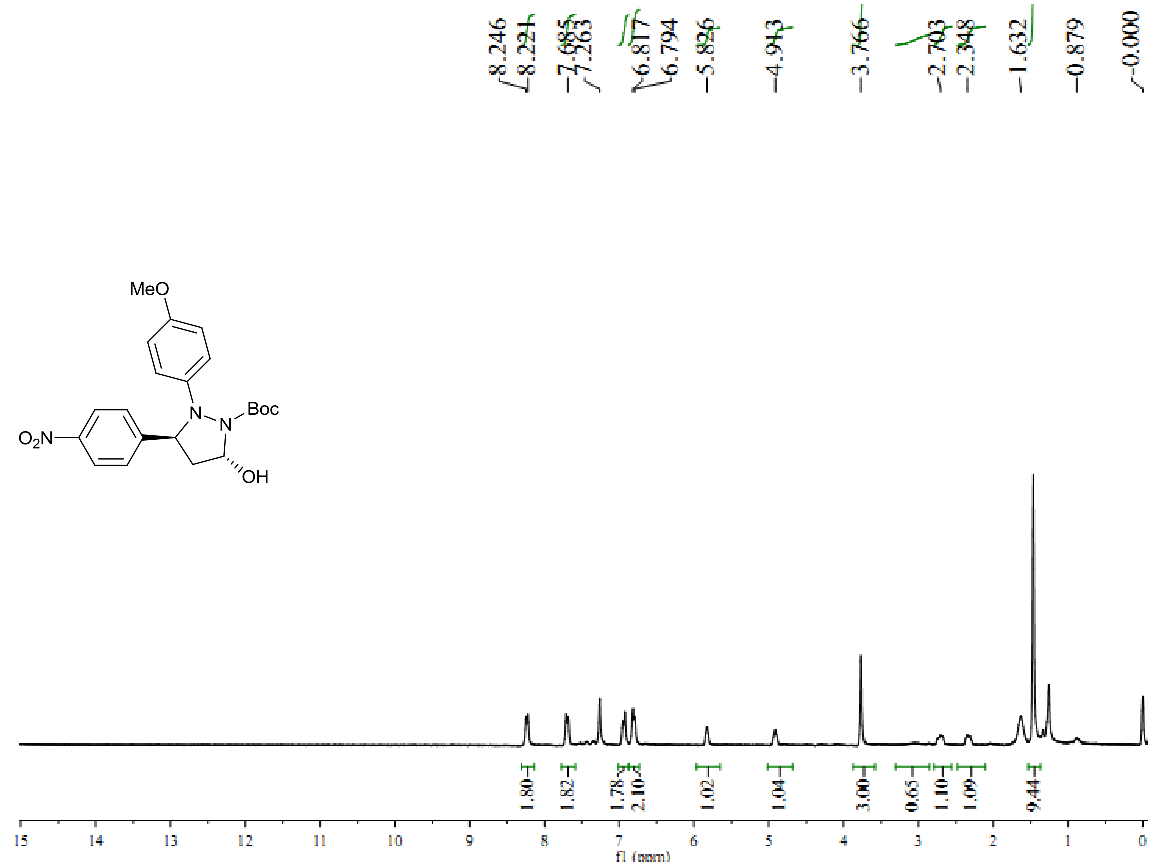
8.246
8.221
7.685
7.263
6.817
6.794
5.826
4.913
3.766
2.703
2.348
1.632
0.879
0.000
MeO
N
N
Boc
O2N
OH
1.80
1.82
1.78
2.10
1.02
1.04
3.00
0.65
1.10
1.09
9.44
f1 (ppm)

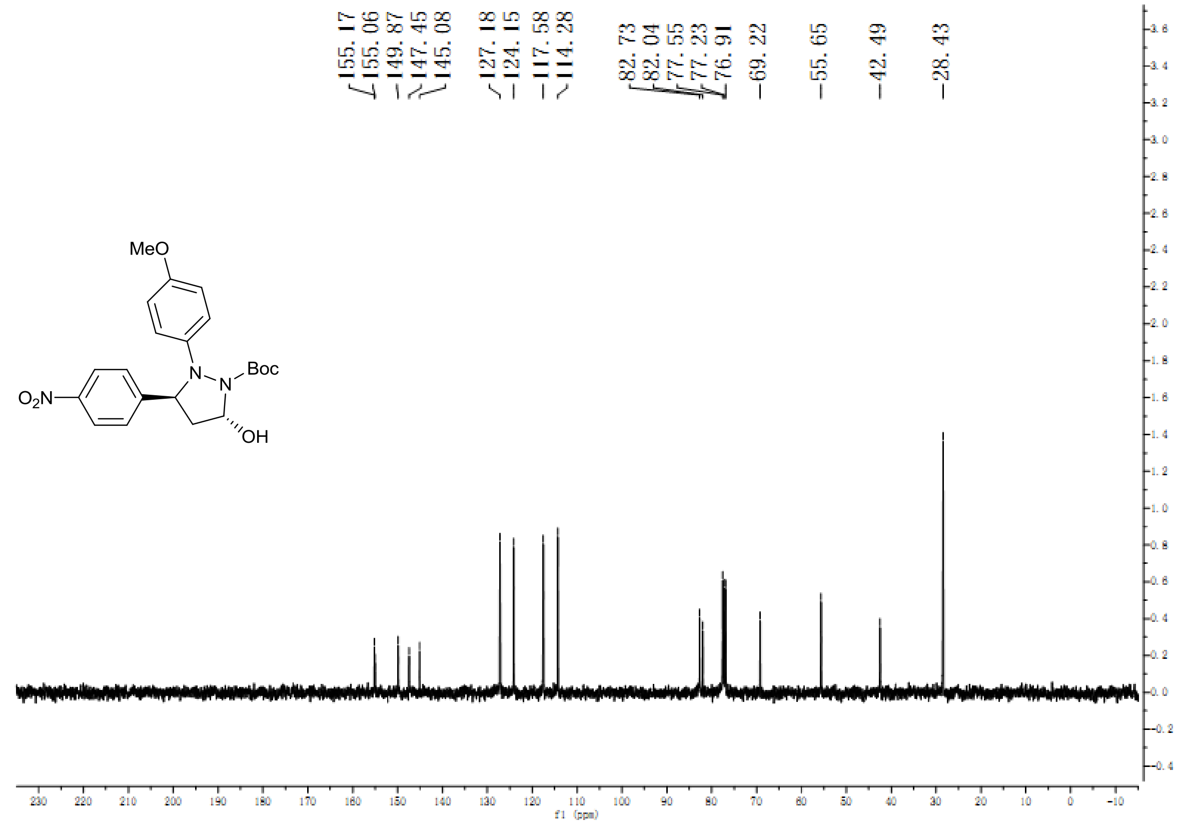
155.17
155.06
149.87
147.45
145.08
127.18
124.15
117.58
114.28
82.73
82.04
77.55
77.23
76.91
69.22
55.65
42.49
28.43
MeO
N
N
Boc
O2N
OH
f1 (ppm)

**4v**

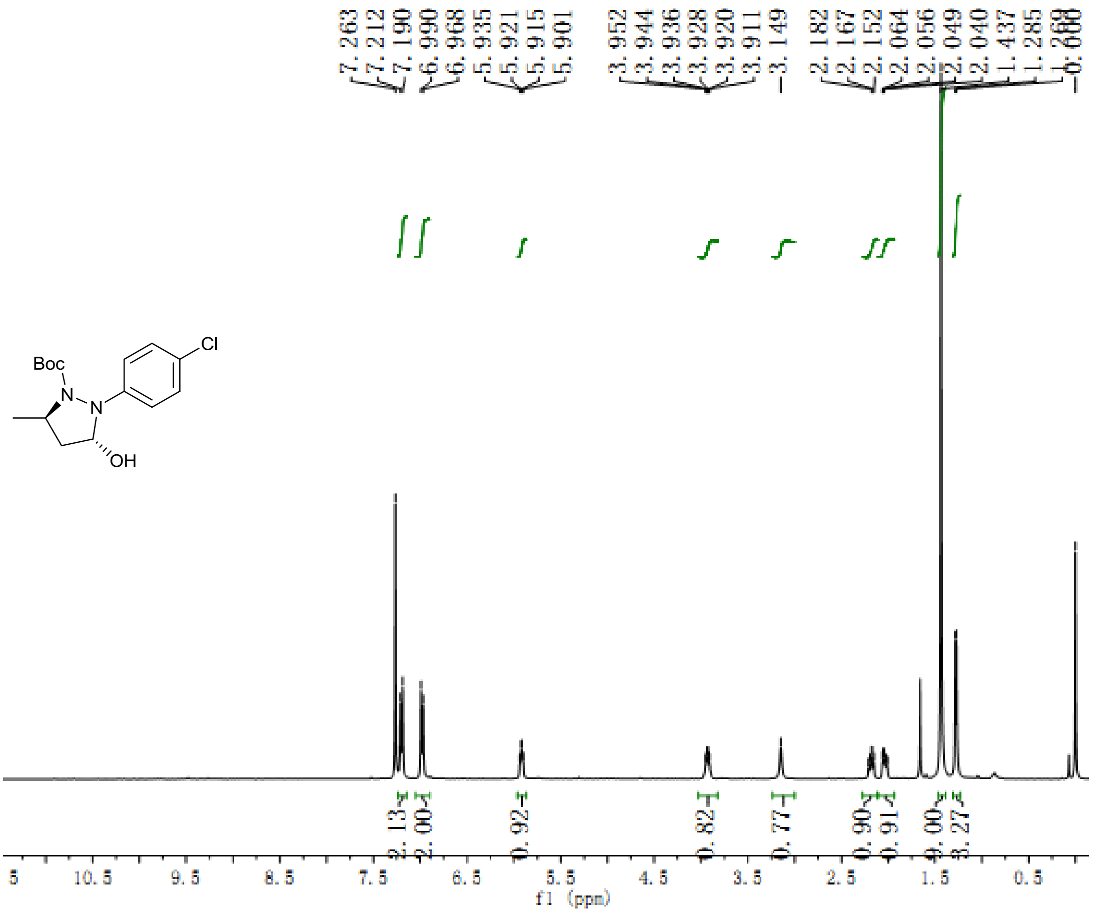
7.263
7.212
7.190
6.990
6.968
5.935
5.921
5.915
5.901
3.952
3.944
3.936
3.928
3.920
3.911
3.149
2.182
2.167
2.152
2.064
2.056
2.049
2.040
1.437
1.285
Boc
N
N
Cl
OH
2.13
2.00
0.92
0.82
0.77
0.90
0.91
9.00
3.27
f1 (ppm)

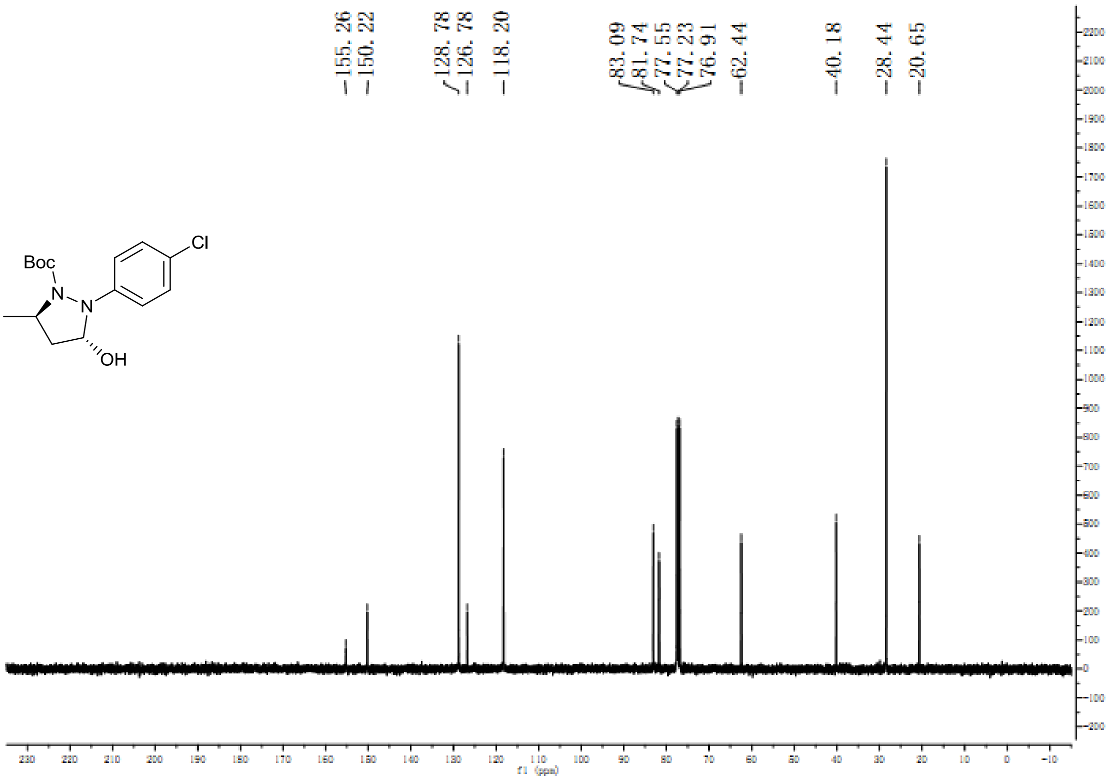
155.26
150.22
128.78
126.78
118.20
83.09
81.74
77.55
77.23
76.91
62.44
40.18
28.44
20.65
Boc
N
N
Cl
OH

**4w**

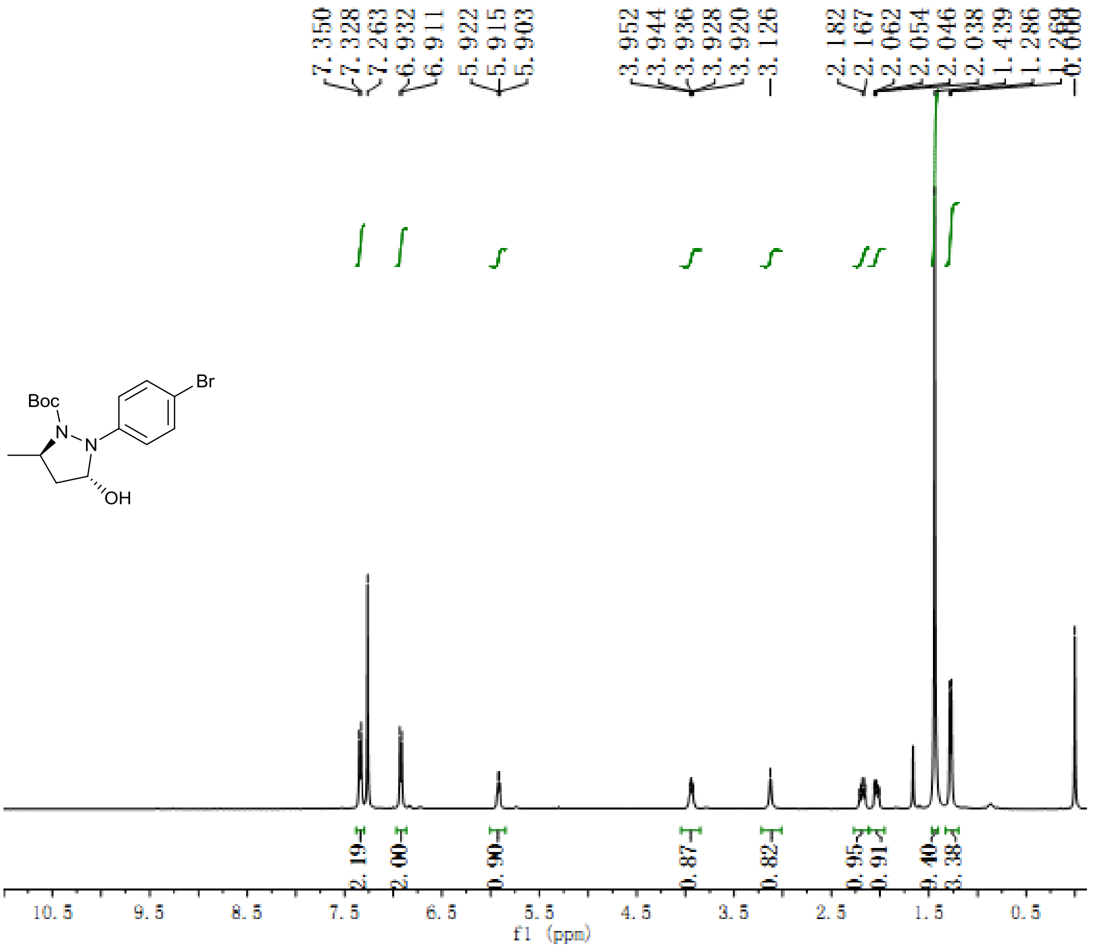

7.350
7.328
7.263
6.932
6.911
5.922
5.915
5.903
3.952
3.944
3.936
3.928
3.920
3.126
2.182
2.167
2.062
2.054
2.046
2.038
1.439
1.286
1.269
0.000
Boc
N
N
Br
OH
2.19
2.00
0.90
0.87
0.82
0.95
0.91
9.40
3.38
f1 (ppm)


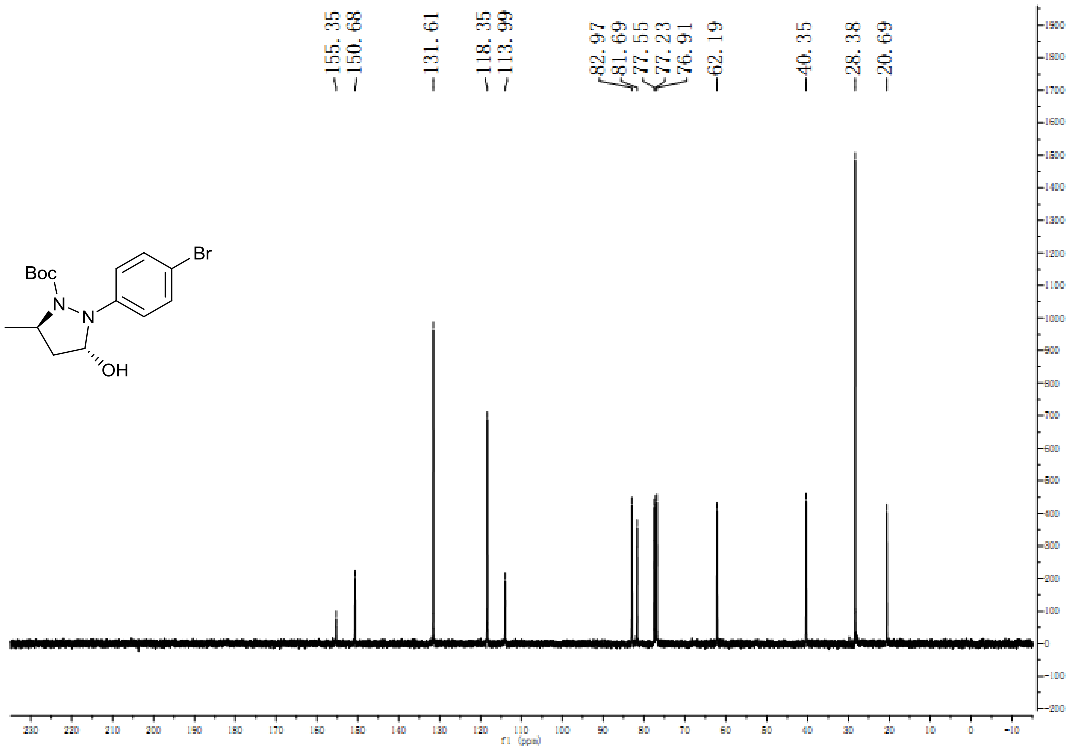

155.35
150.68
131.61
118.35
113.99
82.97
81.69
77.55
77.23
76.91
62.19
40.35
28.38
20.69
Boc
N
N
Br
OH
f1 (ppm)

## 6. HPLC spectra

## 4a

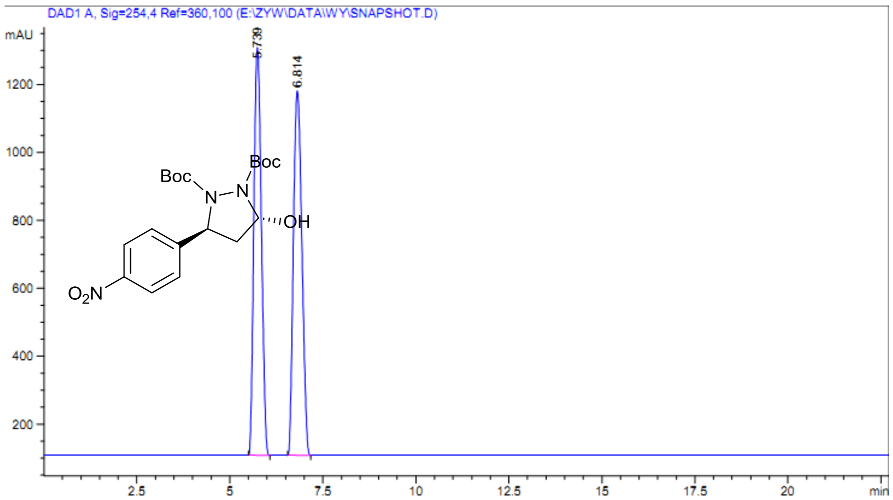


| Peak # | RetTime [min] | Type | Width [min] | Area [mAU*s] | Height [mAU] | Area % |
|---|---|---|---|---|---|---|
| 1 | 5.739 | BV | 0.2230 | 1.67629e4 | 1199.90112 | 49.6917 |
| 2 | 6.814 | BV | 0.2546 | 1.69709e4 | 1072.82996 | 50.3083 |

Totals : 3.37339e4 2272.73108

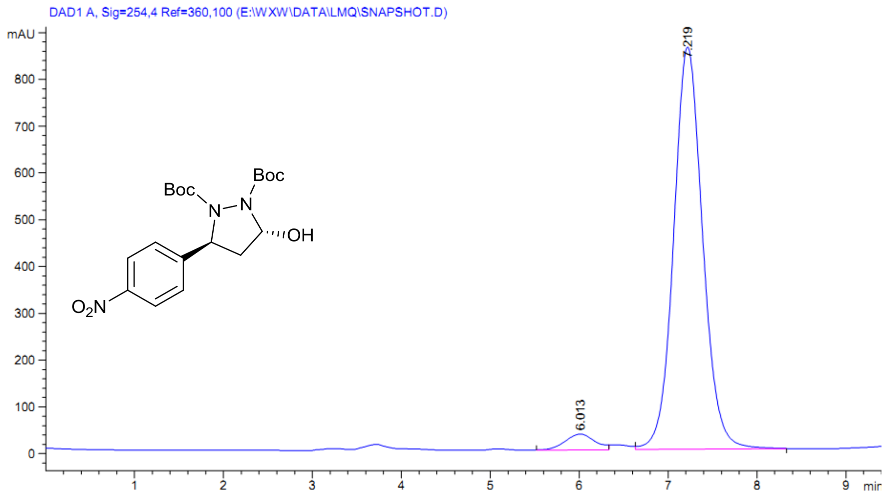


| Peak # | RetTime [min] | Type | Width [min] | Area [mAU*s] | Height [mAU] | Area % |
|---|---|---|---|---|---|---|
| 1 | 6.013 | BV | 0.3515 | 777.24738 | 33.64997 | 3.8339 |
| 2 | 7.219 | VB | 0.3465 | 1.94957e4 | 859.92670 | 96.1661 |

Totals : 2.02730e4 893.57666

## 4b

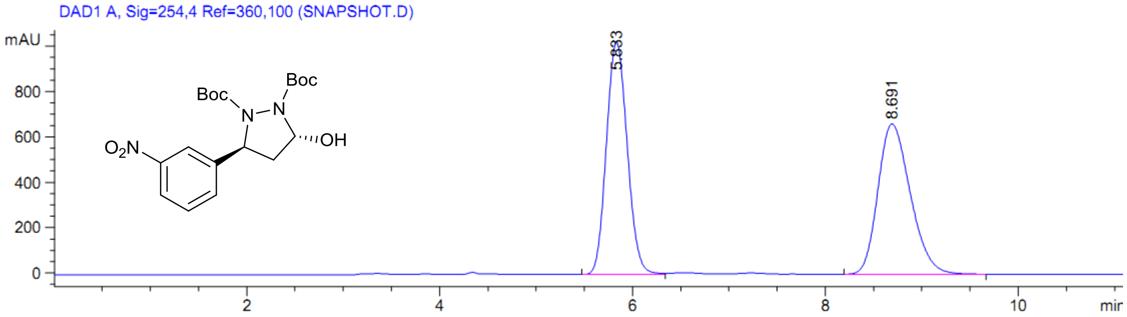


| Peak # | RetTime [min] | Type | Width [min] | Area [mAU*s] | Height [mAU] | Area % |
|---|---|---|---|---|---|---|
| 1 | 5.833 | VV | 0.2370 | 1.53792e4 | 1025.35144 | 49.7774 |
| 2 | 8.691 | BB | 0.3629 | 1.55168e4 | 663.41791 | 50.2226 |

Totals : 3.08960e4 1688.76935

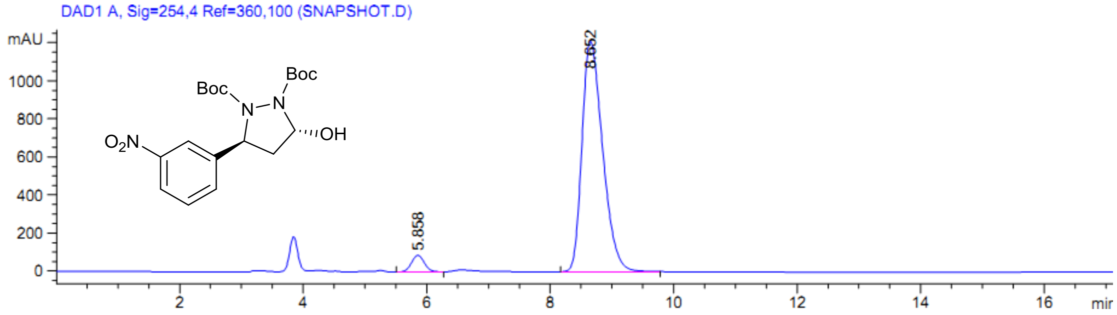


| Peak # | RetTime [min] | Type | Width [min] | Area [mAU*s] | Height [mAU] | Area % |
|---|---|---|---|---|---|---|
| 1 | 5.858 | VB | 0.2265 | 1255.31323 | 86.92343 | 4.2799 |
| 2 | 8.652 | BB | 0.3585 | 2.80755e4 | 1210.80688 | 95.7201 |

Totals : 2.93308e4 1297.73032

**4c**

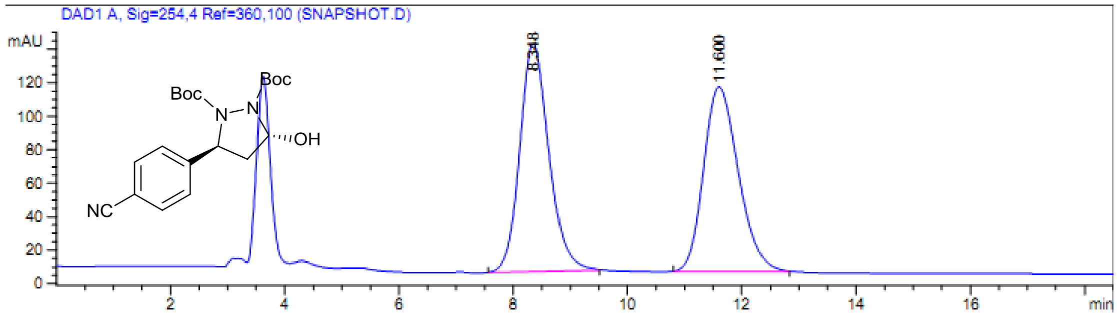


| Peak # | RetTime [min] | Type | Width [min] | Area [mAU*s] | Height [mAU] | Area % |
|---|---|---|---|---|---|---|
| 1 | 8.348 | BB | 0.5441 | 4833.70898 | 136.19106 | 50.6735 |
| 2 | 11.600 | BB | 0.6592 | 4705.22168 | 110.41570 | 49.3265 |

Totals : 9538.93066 246.60675

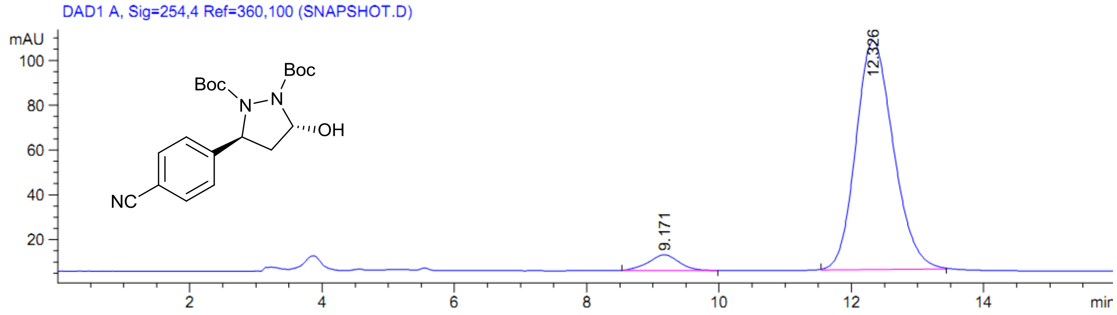


| Peak # | RetTime [min] | Type | Width [min] | Area [mAU*s] | Height [mAU] | Area % |
|---|---|---|---|---|---|---|
| 1 | 9.171 | BB | 0.4806 | 225.81580 | 6.95804 | 5.3203 |
| 2 | 12.326 | BB | 0.6095 | 4018.63599 | 102.02798 | 94.6797 |

Totals : 4244.45178 108.98602

**4d**

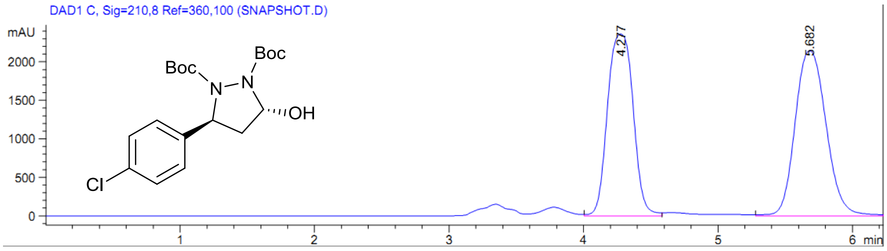


| Peak # | RetTime [min] | Type | Width [min] | Area [mAU*s] | Height [mAU] | Area % |
|---|---|---|---|---|---|---|
| 1 | 4.277 | VV | 0.2146 | 3.13104e4 | 2362.74780 | 47.6812 |
| 2 | 5.682 | VBA | 0.2527 | 3.43558e4 | 2146.62744 | 52.3188 |

Totals : 6.56663e4 4509.37524

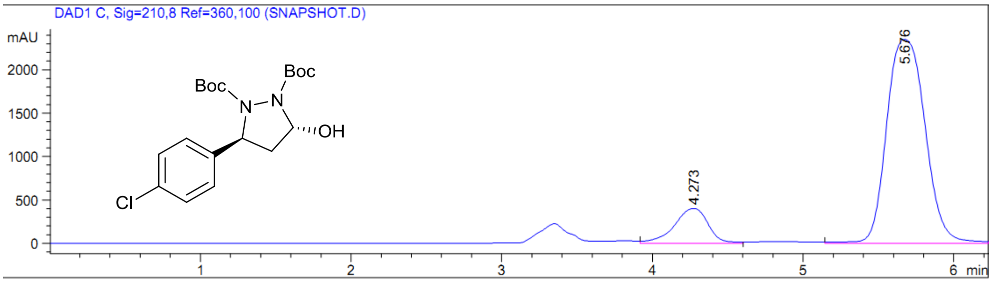


| Peak # | RetTime [min] | Type | Width [min] | Area [mAU*s] | Height [mAU] | Area % |
|---|---|---|---|---|---|---|
| 1 | 4.273 | VV | 0.2430 | 6286.99902 | 400.71469 | 13.0882 |
| 2 | 5.676 | VBA | 0.2831 | 4.17485e4 | 2351.78198 | 86.9118 |

Totals : 4.80355e4 2752.49667

**4e**

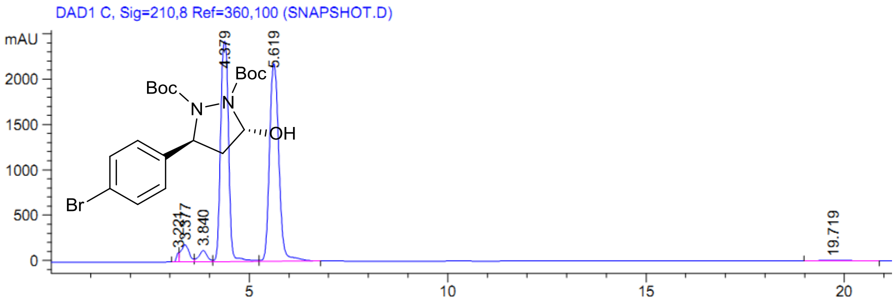


| Peak # | RetTime [min] | Type | Width [min] | Area [mAU*s] | Height [mAU] | Area % |
|---|---|---|---|---|---|---|
| 1 | 3.221 | BV | 0.0665 | 419.58740 | 103.62377 | 0.5602 |
| 2 | 3.377 | VV | 0.1906 | 2664.85400 | 188.93449 | 3.5579 |
| 3 | 3.840 | VV | 0.2150 | 1802.23474 | 124.38641 | 2.4062 |
| 4 | 4.379 | VV | 0.2275 | 3.40490e4 | 2429.74316 | 45.4593 |
| 5 | 5.619 | VB | 0.2596 | 3.57763e4 | 2178.91650 | 47.7654 |
| 6 | 19.719 | BB | 0.6511 | 188.01134 | 3.67339 | 0.2510 |

Totals : 7.49000e4 5029.27773

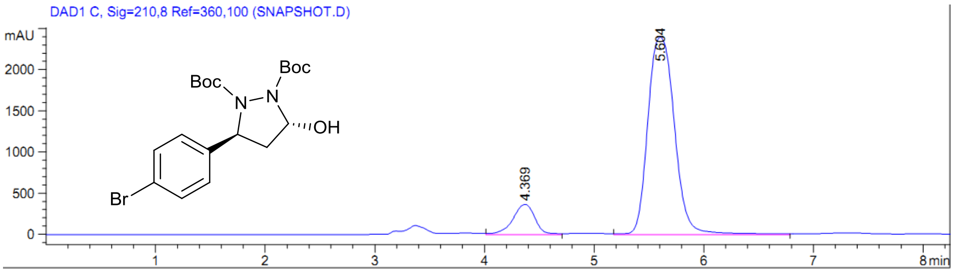


| Peak # | RetTime [min] | Type | Width [min] | Area [mAU*s] | Height [mAU] | Area % |
|---|---|---|---|---|---|---|
| 1 | 4.369 | VV | 0.2187 | 5168.65137 | 366.23688 | 11.3073 |
| 2 | 5.604 | VB | 0.2674 | 4.05419e4 | 2395.49609 | 88.6927 |

Totals : 4.57105e4 2761.73297

**4h**

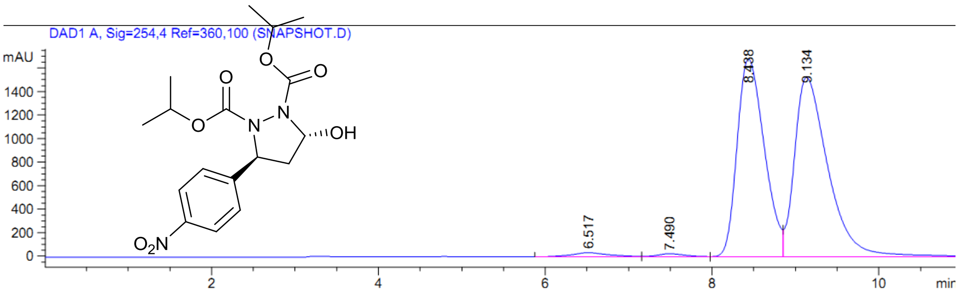


| Peak # | RetTime [min] | Type | Width [min] | Area [mAU*s] | Height [mAU] | Area % |
|---|---|---|---|---|---|---|
| 1 | 6.517 | BV | 0.4349 | 1180.11609 | 37.52028 | 1.4272 |
| 2 | 7.490 | VV | 0.3694 | 646.00006 | 26.22237 | 0.7812 |
| 3 | 8.438 | VV | 0.3516 | 3.80539e4 | 1684.24658 | 46.0201 |
| 4 | 9.134 | VBA | 0.4230 | 4.28097e4 | 1527.50183 | 51.7715 |

Totals : 8.26897e4 3275.49106

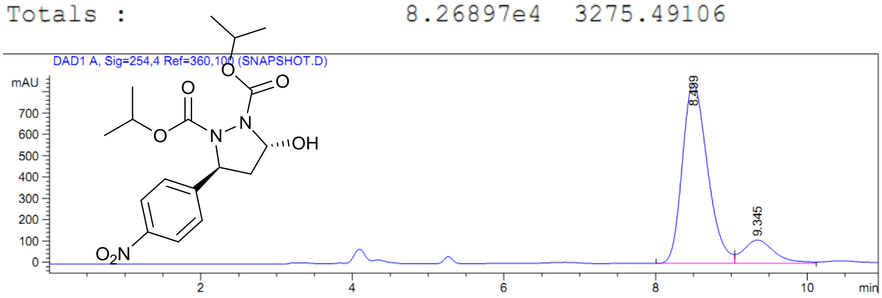


| Peak # | RetTime [min] | Type | Width [min] | Area [mAU*s] | Height [mAU] | Area % |
|---|---|---|---|---|---|---|
| 1 | 8.499 | BV | 0.3601 | 1.94746e4 | 841.03552 | 86.0585 |
| 2 | 9.345 | VV | 0.4301 | 3154.87695 | 109.49943 | 13.9415 |

Totals : 2.26295e4 950.53495

**4n**

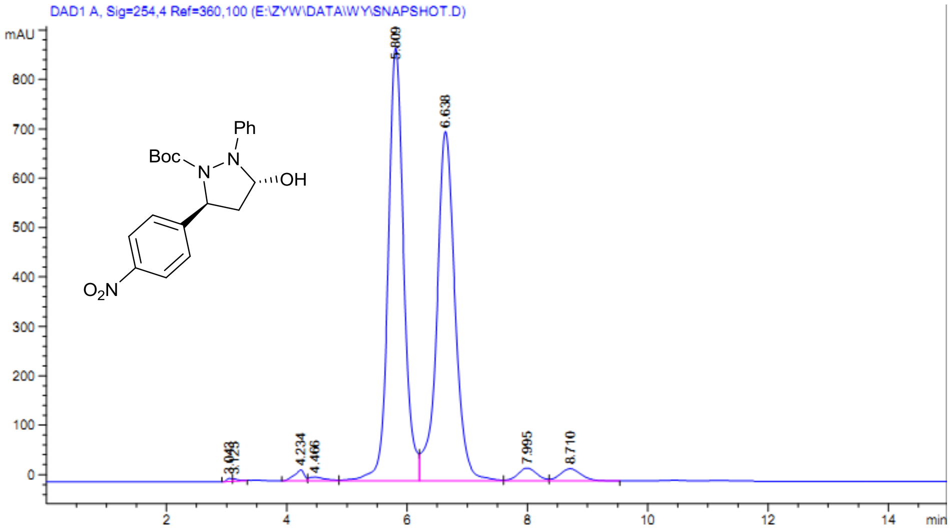


| Peak # | RetTime [min] | Type | Width [min] | Area [mAU*s] | Height [mAU] | Area % |
|---|---|---|---|---|---|---|
| 1 | 3.043 | BV | 0.0847 | 33.97286 | 6.12973 | 0.1053 |
| 2 | 3.125 | VB | 0.0972 | 32.05501 | 4.49603 | 0.0993 |
| 3 | 4.234 | BV | 0.1677 | 264.86191 | 22.76746 | 0.8209 |
| 4 | 4.466 | VV | 0.2567 | 151.46112 | 8.00756 | 0.4694 |
| 5 | 5.809 | VV | 0.2729 | 1.57048e4 | 876.90344 | 48.6745 |
| 6 | 6.638 | VB | 0.3157 | 1.49001e4 | 707.59149 | 46.1802 |
| 7 | 7.995 | BV | 0.3388 | 575.28510 | 26.13980 | 1.7830 |
| 8 | 8.710 | VB | 0.3766 | 602.47772 | 24.69806 | 1.8673 |
| Totals : | | | | 3.22650e4 | 1676.73357 | |

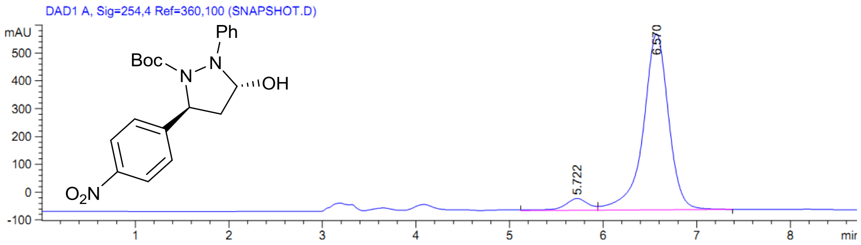


| Peak # | RetTime [min] | Type | Width [min] | Area [mAU*s] | Height [mAU] | Area % |
|---|---|---|---|---|---|---|
| 1 | 5.722 | VV | 0.2544 | 721.86334 | 42.02257 | 5.7000 |
| 2 | 6.570 | VB | 0.2844 | 1.19423e4 | 632.28943 | 94.3000 |
| Totals : | | | | 1.26642e4 | 674.31200 | |

**4n’**

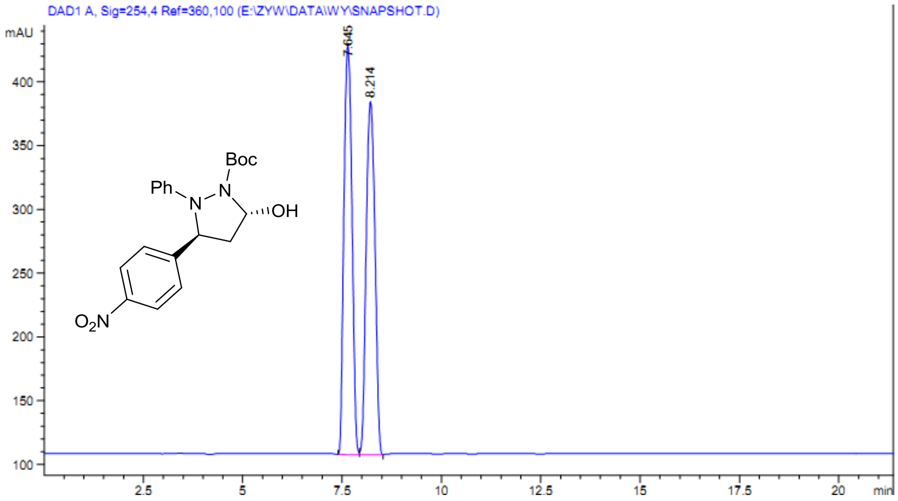


| Peak # | RetTime [min] | Type | Width [min] | Area [mAU*s] | Height [mAU] | Area % |
|---|---|---|---|---|---|---|
| 1 | 7.645 | BV | 0.2379 | 4508.03760 | 320.89066 | 51.4556 |
| 2 | 8.214 | VV | 0.2592 | 4252.98438 | 276.89981 | 48.5444 |
| Totals : | | | | 8761.02197 | 597.79047 | |

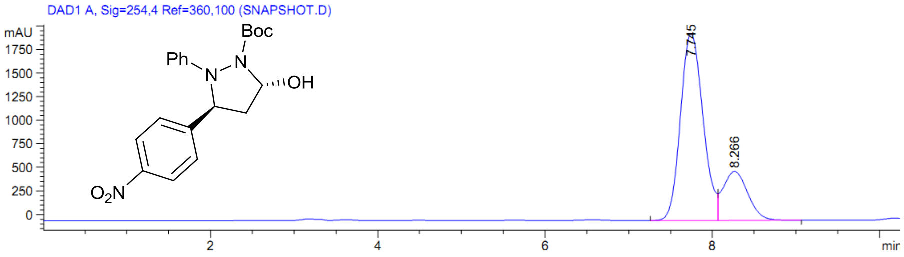


| Peak # | RetTime [min] | Type | Width [min] | Area [mAU*s] | Height [mAU] | Area % |
|---|---|---|---|---|---|---|
| 1 | 7.745 | BV | 0.2949 | 3.71157e4 | 1978.49060 | 77.6262 |
| 2 | 8.266 | VB | 0.3145 | 1.06977e4 | 518.99969 | 22.3738 |
| Totals : | | | | 4.78134e4 | 2497.49030 | |

**4o**

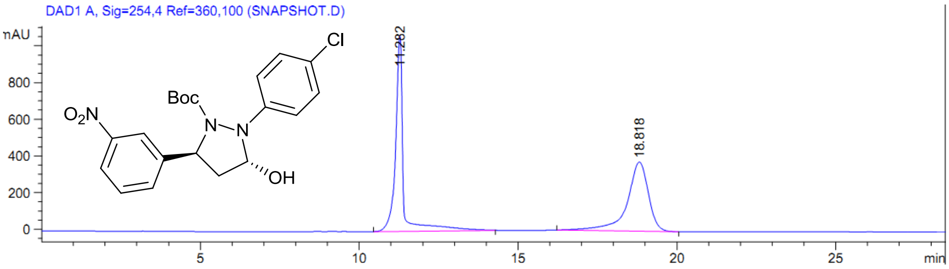


| Peak # | RetTime [min] | Type | Width [min] | Area [mAU*s] | Height [mAU] | Area % |
|---|---|---|---|---|---|---|
| 1 | 11.282 | BB | 0.2425 | 1.79432e4 | 1065.31299 | 50.4654 |
| 2 | 18.818 | BB | 0.6953 | 1.76122e4 | 376.81384 | 49.5346 |

Totals : 3.55554e4 1442.12683

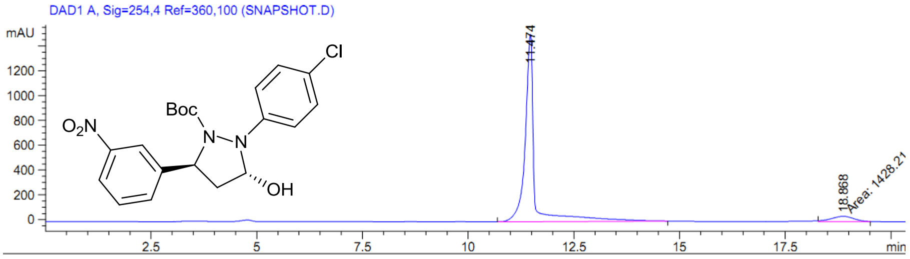


| Peak # | RetTime [min] | Type | Width [min] | Area [mAU*s] | Height [mAU] | Area % |
|---|---|---|---|---|---|---|
| 1 | 11.474 | BB | 0.2168 | 2.29001e4 | 1510.84888 | 94.1294 |
| 2 | 18.868 | MM | 0.5959 | 1428.20569 | 39.94640 | 5.8706 |

Totals : 2.43283e4 1550.79528

**4p**

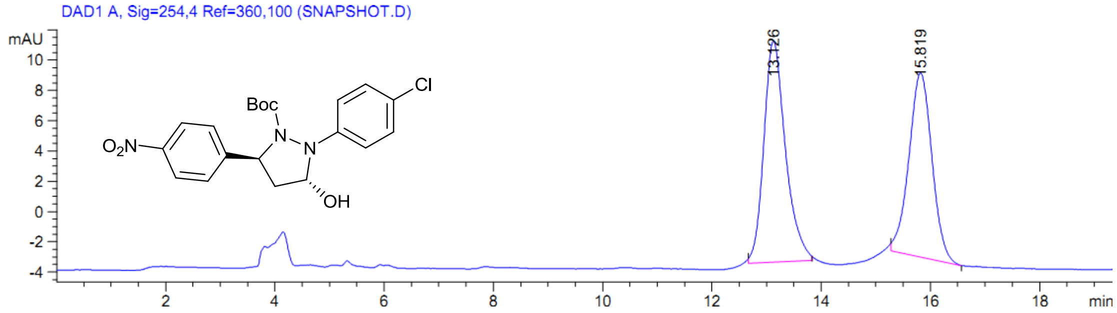


| Peak # | RetTime [min] | Type | Width [min] | Area [mAU*s] | Height [mAU] | Area % |
|---|---|---|---|---|---|---|
| 1 | 13.126 | BB | 0.4110 | 402.69333 | 14.63496 | 53.0819 |
| 2 | 15.819 | BB | 0.4515 | 355.93338 | 12.15998 | 46.9181 |

Totals : 758.62671 26.79494

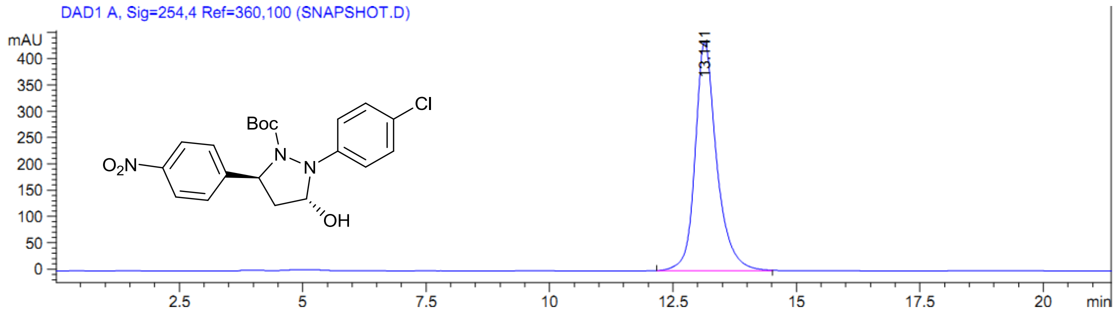


| Peak # | RetTime [min] | Type | Width [min] | Area [mAU*s] | Height [mAU] | Area % |
|---|---|---|---|---|---|---|
| 1 | 13.141 | BB | 0.4503 | 1.32145e4 | 435.12561 | 100.0000 |

Totals : 1.32145e4 435.12561

**4q**

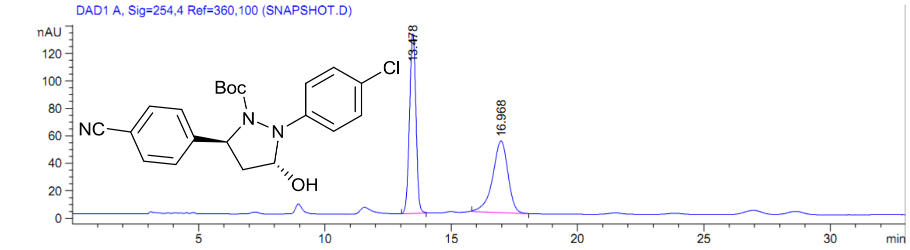


| Peak # | RetTime [min] | Type | Width [min] | Area [mAU*s] | Height [mAU] | Area % |
|---|---|---|---|---|---|---|
| 1 | 13.478 | BB | 0.2803 | 2312.92480 | 130.79883 | 51.2717 |
| 2 | 16.968 | BB | 0.6386 | 2198.19312 | 52.27586 | 48.7283 |
| Totals : | | | | 4511.11792 | 183.07469 | |

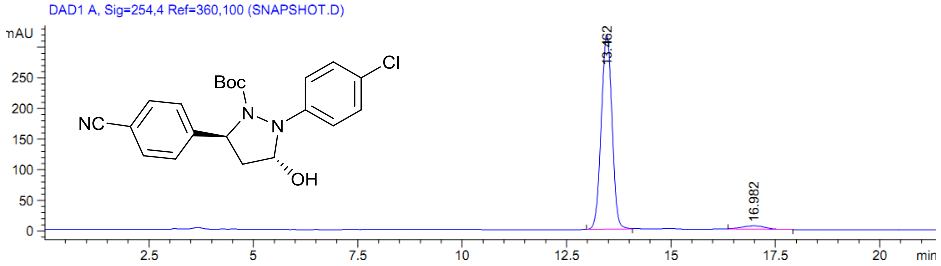


| Peak # | RetTime [min] | Type | Width [min] | Area [mAU*s] | Height [mAU] | Area % |
|---|---|---|---|---|---|---|
| 1 | 13.462 | BB | 0.2832 | 5680.49854 | 316.81180 | 96.6309 |
| 2 | 16.982 | BB | 0.5374 | 198.05397 | 5.45727 | 3.3691 |
| Totals : | | | | 5878.55251 | 322.26907 | |

**4r**

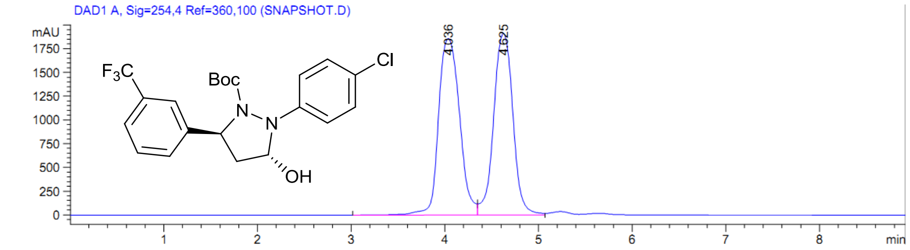


| Peak # | RetTime [min] | Type | Width [min] | Area [mAU*s] | Height [mAU] | Area % |
|---|---|---|---|---|---|---|
| 1 | 4.036 | BV | 0.2557 | 2.89939e4 | 1861.48999 | 51.4682 |
| 2 | 4.625 | VV | 0.2268 | 2.73398e4 | 1911.89856 | 48.5318 |
| Totals : | | | | 5.63337e4 | 3773.38855 | |

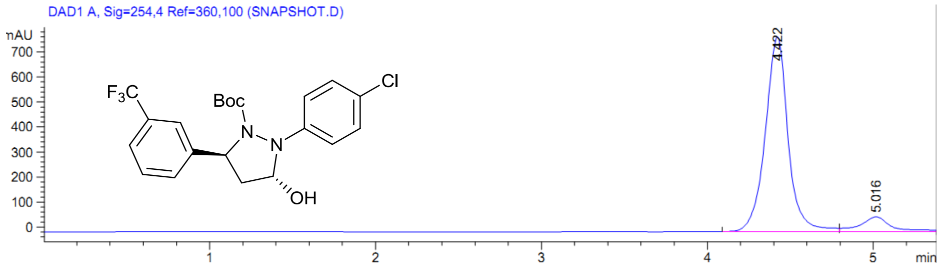


| Peak # | RetTime [min] | Type | Width [min] | Area [mAU*s] | Height [mAU] | Area % |
|---|---|---|---|---|---|---|
| 1 | 4.422 | BV | 0.1442 | 7497.35645 | 779.29291 | 90.5720 |
| 2 | 5.016 | VBA | 0.1852 | 780.42657 | 59.45105 | 9.4280 |
| Totals : | | | | 8277.78302 | 838.74395 | |

**4s**

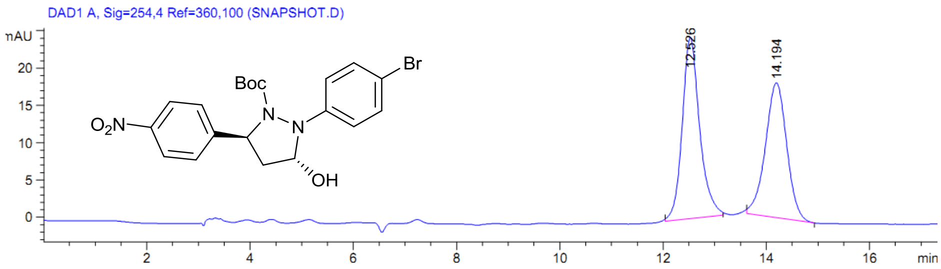


| Peak # | RetTime [min] | Type | Width [min] | Area [mAU*s] | Height [mAU] | Area % |
|---|---|---|---|---|---|---|
| 1 | 12.526 | BB | 0.3511 | 567.27557 | 24.23341 | 52.2384 |
| 2 | 14.194 | BB | 0.4404 | 518.66034 | 18.09674 | 47.7616 |

Totals : 1085.93591 42.33016

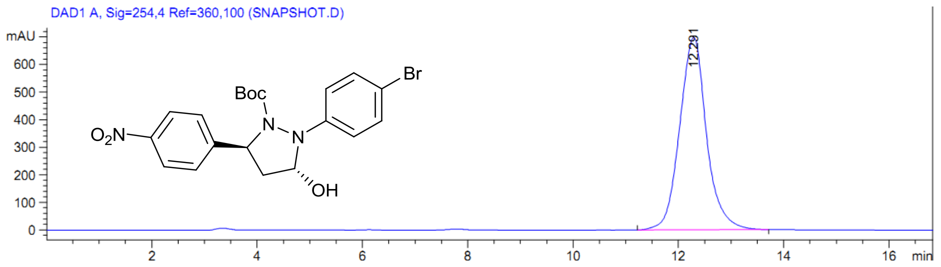


| Peak # | RetTime [min] | Type | Width [min] | Area [mAU*s] | Height [mAU] | Area % |
|---|---|---|---|---|---|---|
| 1 | 12.291 | BB | 0.5421 | 2.47936e4 | 698.52704 | 100.0000 |

Totals : 2.47936e4 698.52704

**4t**

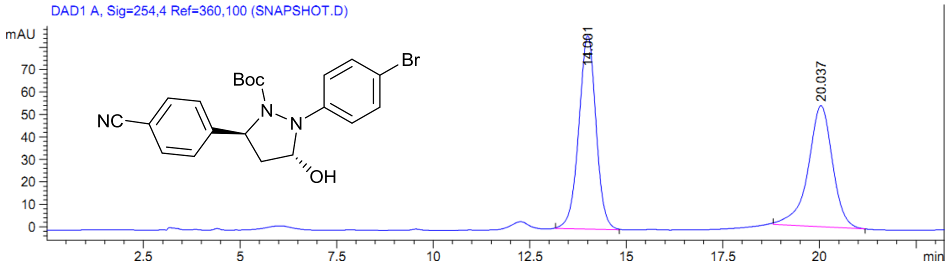


| Peak # | RetTime [min] | Type | Width [min] | Area [mAU*s] | Height [mAU] | Area % |
|---|---|---|---|---|---|---|
| 1 | 14.001 | BB | 0.4759 | 2673.70679 | 86.19019 | 52.9521 |
| 2 | 20.037 | BB | 0.6606 | 2375.58862 | 54.06293 | 47.0479 |

Totals : 5049.29541 140.25311

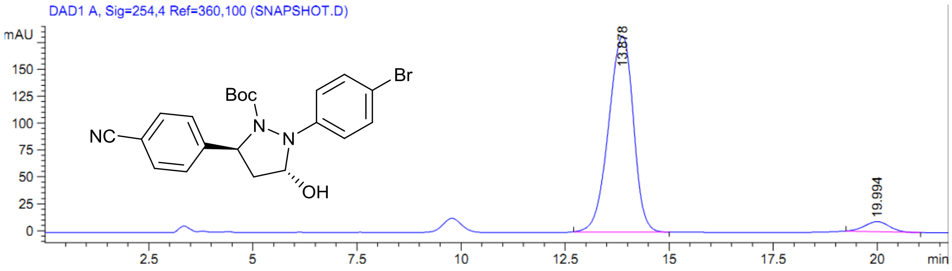


| Peak # | RetTime [min] | Type | Width [min] | Area [mAU*s] | Height [mAU] | Area % |
|---|---|---|---|---|---|---|
| 1 | 13.878 | BB | 0.6524 | 7573.98975 | 182.40045 | 95.1768 |
| 2 | 19.994 | BB | 0.6235 | 383.82449 | 9.37711 | 4.8232 |

Totals : 7957.81424 191.77756

**4u’**

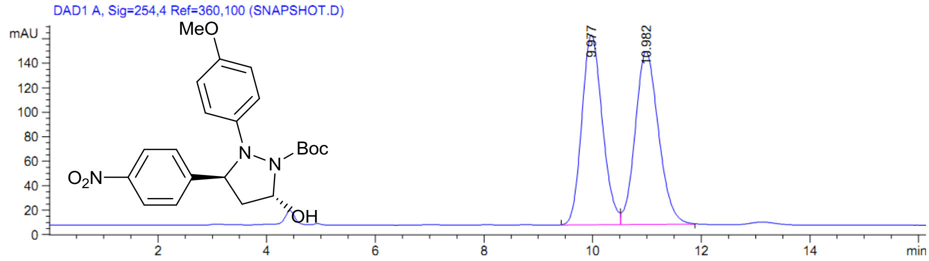


| Peak # | RetTime [min] | Type | Width [min] | Area [mAU*s] | Height [mAU] | Area % |
|---|---|---|---|---|---|---|
| 1 | 9.977 | BV | 0.4087 | 4077.57129 | 155.10695 | 49.1733 |
| 2 | 10.982 | VB | 0.4625 | 4214.68115 | 141.07733 | 50.8267 |

Totals : 8292.25244 296.18428

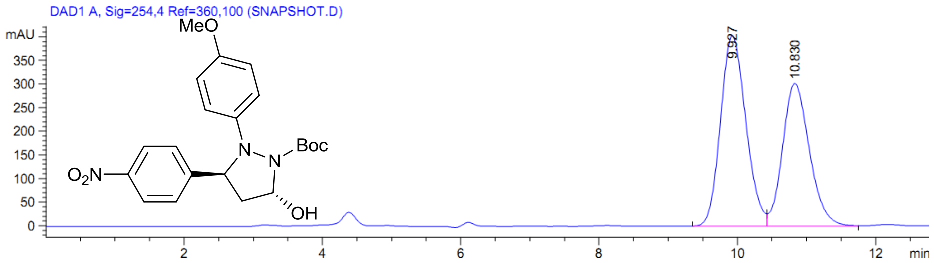


| Peak # | RetTime [min] | Type | Width [min] | Area [mAU*s] | Height [mAU] | Area % |
|---|---|---|---|---|---|---|
| 1 | 9.927 | BV | 0.3969 | 1.02598e4 | 403.09756 | 55.6428 |
| 2 | 10.830 | VB | 0.4176 | 8178.88818 | 302.31866 | 44.3572 |

Totals : 1.84387e4 705.41623

**4v**

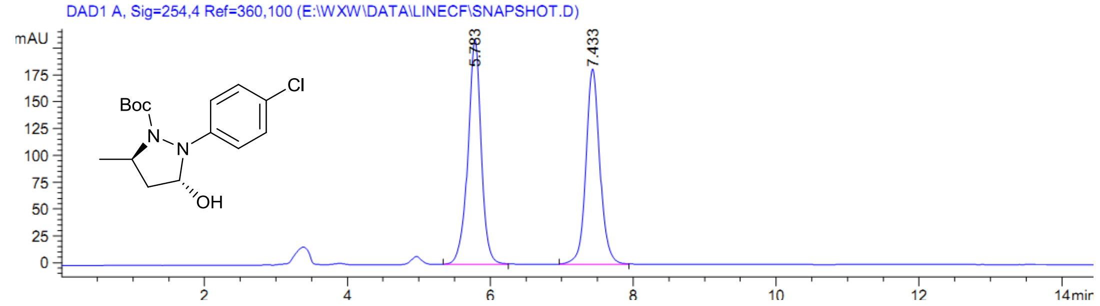


| Peak # | RetTime [min] | Type | Width [min] | Area [mAU*s] | Height [mAU] | Area % |
|---|---|---|---|---|---|---|
| 1 | 5.783 | BB | 0.1927 | 2700.02515 | 209.00623 | 52.2106 |
| 2 | 7.433 | BB | 0.2064 | 2471.38770 | 181.97112 | 47.7894 |

Totals : 5171.41284 390.97734

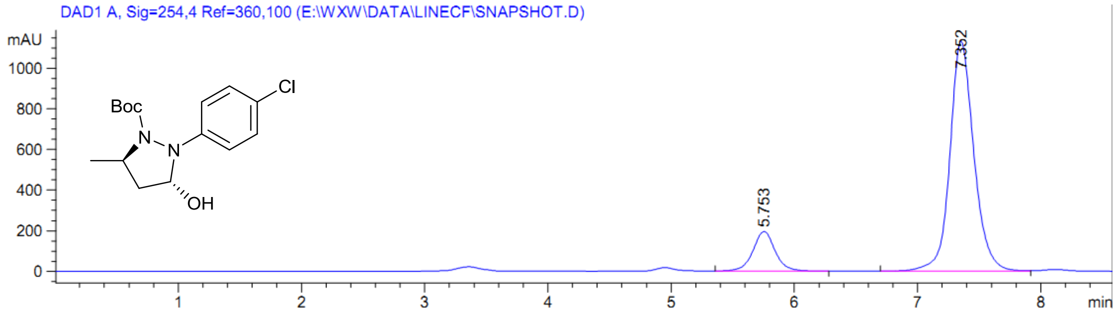


| Peak # | RetTime [min] | Type | Width [min] | Area [mAU*s] | Height [mAU] | Area % |
|---|---|---|---|---|---|---|
| 1 | 5.753 | BB | 0.1898 | 2479.35278 | 195.70689 | 13.9110 |
| 2 | 7.352 | BV | 0.2062 | 1.53436e4 | 1131.24634 | 86.0890 |

Totals : 1.78229e4 1326.95323

**4w**

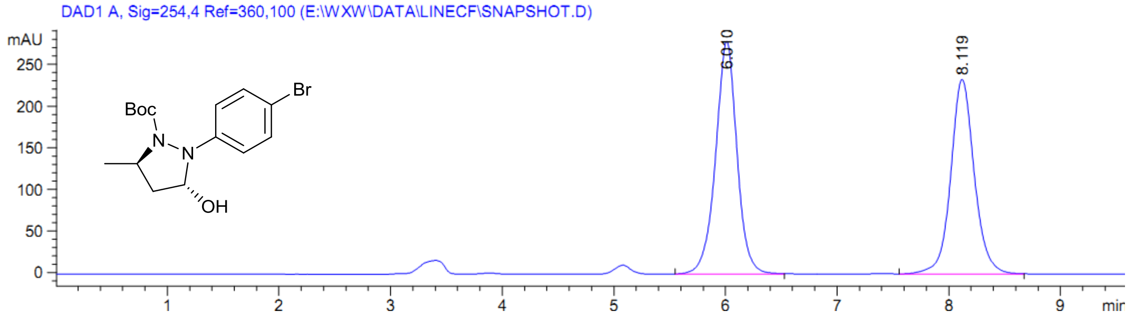


| Peak # | RetTime [min] | Type | Width [min] | Area [mAU*s] | Height [mAU] | Area % |
|---|---|---|---|---|---|---|
| 1 | 6.010 | BB | 0.1994 | 3720.07837 | 279.23526 | 52.0093 |
| 2 | 8.119 | VB | 0.2236 | 3432.63477 | 233.32574 | 47.9907 |

Totals : 7152.71313 512.56100

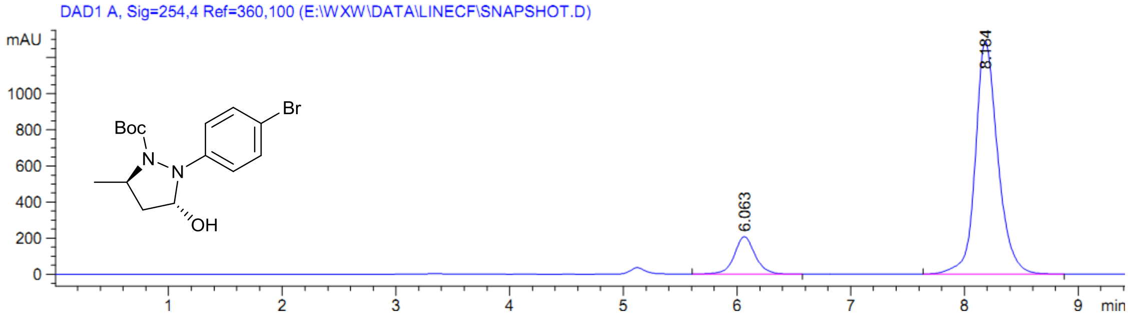


| Peak # | RetTime [min] | Type | Width [min] | Area [mAU*s] | Height [mAU] | Area % |
|---|---|---|---|---|---|---|
| 1 | 6.063 | BB | 0.1944 | 2685.58301 | 208.35497 | 13.1815 |
| 2 | 8.184 | BB | 0.2062 | 1.76884e4 | 1287.95874 | 86.8185 |

Totals : 2.03739e4 1496.31371
